# A Modular Approach to Asymmetric Mg(I) Complexes Using a Unique Cyclopentadienyl Mg(I) Complex

**DOI:** 10.1002/anie.202518538

**Published:** 2025-10-17

**Authors:** Hannah Stecher, Stefan Thum, Johannes Maurer, Jonathan Mai, Marcel A. Schmidt, Jens Langer, Sjoerd Harder

**Affiliations:** ^1^ Inorganic and Organometallic Chemistry Friedrich‐Alexander‐Universität Erlangen‐Nürnberg Egerlandstraße 1 91058 Erlangen Germany

**Keywords:** Calcium, DFT, Ligand exchange, Low‐valent, Magnesium

## Abstract

The first Mg^I^ complexes with non‐N ligands have been isolated and were fully characterized. Reaction of [(BDI*)MgNa]_2_ (**III**) with MgCp*_2_ gave quantitative conversion to (BDI*)MgMgCp* (**1**); BDI* = HC[*t*BuC = N(DIPeP)]_2_, DIPeP = 2,6‐Et_2_CH‐phenyl) and Cp* = pentamethylcyclopentadienyl. A similar reaction with CaCp*_2_ did not give (BDI*)MgCaCp* but led to insertion of the formal Mg° centre in the benzylic C─H bond, an oxidative addition that resulted in a mixed Mg/Ca hydride complex. The Cp* ligand in the unique (BDI*)MgMgCp* complex can be exchanged by addition of LM (M = Li, Na, K) complexes, resulting in formation of (BDI*)MgMgL and insoluble (Cp*M)_n_. This modular approach worked for Cp*/N(SiMe_3_)_2_ exchange but not for the substitution of Cp* by P(SiMe_3_)_2_ or other PR_2_ ligands. Reaction of (BDI*)MgMgCp* with a bulky NaOAr reagent led to quantitative formation of (BDI*)MgMgOAr·THF, a first Mg^I^ complex stabilized with an aryloxide ligand. All products have been fully characterized by X‐ray diffraction and NMR methods. A computational study gives insight in the thermodynamics of formation and electronic structures.

## Graphical abstract

**Like changing the tire of a car**: The synthesis of a unique Mg^I^ complex with a Cp* ligand enabled facile ligand exchange by salt metathesis, giving access to a first aryloxide‐stabilized Mg^I^ complex. This concept paves the way for the syntheses of a large variety of asymmetric Mg^I^ dimers in which one of the Mg^I^ centres is stabilized by a non‐nitrogen ligand.

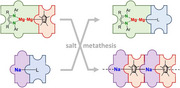

It is now more than two decades ago that Carmona and coworkers discovered a first molecular Zn^I^ complex stabilized by Cp* (Scheme 1, **I**).^[^ ^1^ ^]^ Given the remarkable similarities between Mg and Zn complexes,^[^ ^2^ ^]^ the question was posed whether similar complexes with alkaline‐earth (Ae) metals in the +I oxidation state could exist. A computational study on CpAeAeCp (Ae = Be, Mg, Ca) indicated that these compounds may be stable.^[^ ^3^ ^]^ This triggered world‐wide research activities in Ae^I^ metal chemistry. Jones and coworkers soon found that stabilization of the Mg─Mg bond requires much bulkier bidentate N‐based ligands, such as the β‐diketiminate (BDI) ligand in **II**.^[^ ^4^ ^]^ Only very recently, a first Ae^I^ complex stabilized by Cp ligands could be isolated for the smallest group 2 metal Be in the form of CpBeBeCp.^[^ ^5^ ^]^

**Scheme 1:**
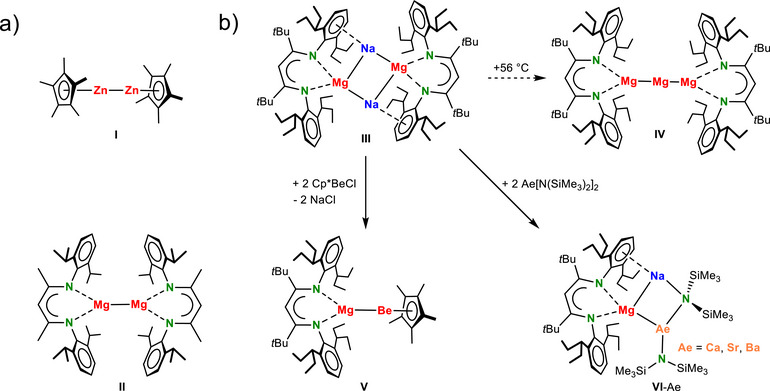
a) The first Zn^I^ (I) and Mg^I^ (**II**) complexes. (b) Further examples of complexes with group 2 metals in low oxidation states (**III**‐**VI**).

Low oxidation state magnesium chemistry has seen a rapid development.^[^ ^6^ ^–‐^ ^11^ ^]^ Nearly 50 different complexes with Mg‐Mg bonds have been reported. Without exception, these are all stabilized with N‐based ligands. Introducing a superbulky β‐diketiminate,^[^ ^12^ ^]^ abbreviated in here as BDI* (BDI* = HC[*t*BuC = N(DIPeP)]_2_, DIPeP = 2,6‐CHEt_2_‐phenyl), we recently isolated first Mg^0^ complexes (**III**, **IV**).^[^ ^13^ ^]^ The sodium magnesyl complex **III** turned out to be the key to heterobimetallic low oxidation state Ae metal complexes, that is, the first formal Be^0^ complex **V** and Mg^0^ complexes **VI**‐Ae.^[^ ^14^, ^15^ ^]^ The superbulky BDI* ligand that has been critical to these developments features long branched alkyl substituents which allows for solution chemistry in most inert alkane solvents. Cp‐type ligands so far only stabilized the low oxidation state Be centres in complexes CpBeBeCp and **V**. Herein, we report a first low oxidation state complex in which one of the considerably larger Mg^I^ centres has been stabilized by a Cp* ligand. We also show that this ligand can be simply exchanged by a salt‐metathesis procedure, potentially giving facile access to a family of asymmetric (BDI*)Mg^I^Mg^I^L complexes. As proof of concept, we herein introduce a unique alkoxide‐stabilized Mg^I^ complex.

Reacting the sodium magnesyl complex [(BDI*)MgNa]_2_ **III** with MgCp*_2_ gave quantitative conversion to (BDI*)MgMgCp* (**1**, Scheme 2). The raw product is essentially pure (yield: 95%, Figure S1). Precipitation of fully insoluble (Cp*Na)_n_ is the driving force in this salt‐metathesis reaction. Due to the very high alkane solubility of **1**, crystals could only be obtained in 42% yield. The crystal structure of **1** shows a rare asymmetric Mg^I^ complex with two different anionic ligands (Figure 1a). Other examples include (BDI*)MgMgN(SiMe_3_)_2_ and a (BDI)MgMg(guanidinate) complex.^[^ ^15^, ^16^ ^]^ In **1**, the Mg‐Mg‐Cp* arrangement is close to linear (Mg‐Mg‐Cp_centroid_ = 174.44(3)° but BDI*‐Mg‐Mg coordination is slightly asymmetric [C2···Mg‐Mg = 169.32(3)°] with unequal N‐Mg‐Mg angles of 122.16(3)/142.62(3)°. This may be caused by crystal packing or attractive dispersive BDI*···Cp* interactions. To date, complex **1** displays the shortest recorded Mg─Mg bond of 2.7748(6) Å. The next shortest Mg─Mg bond has been reported for a (BDI)MgMg(guanidinate) complex (2.7935(6) Å).^[^ ^16^ ^]^ The Mg‐Cp_centroid_ distance of 2.0193(6) Å is significantly larger than that of 1.959(1) Å in the linear magnesocene Mg(*η^5^*‐Cp*)_2_.^[^ ^17^ ^]^ This is not due to steric stress between BDI* and Cp* ligands in **1**. Not only the Mg─Mg bond but also the Mg‐N distances in **1** [2.040(1)/2.043(1) Å] are short. The latter are significantly shorter than the Mg─N bonds for the bidentate ligand in (*η^2^*‐BDI*)MgMg(*η^1^*‐BDI*) [2.061(2)/2.065(2) Å] in which steric stress is an issue.^[^ ^12^ ^]^ The long Mg‐Cp_centroid_ in **1** is the consequence of the lower +I oxidation state of the Mg centre compared to Mg^II^ in Mg(*η^5^*‐Cp*)_2_. Being the first of its kind, the Mg‐Cp_centroid_ distance of 2.0193(6) Å is now the bench‐mark for Cp*‐Mg^I^ coordination.

**Scheme 2:**
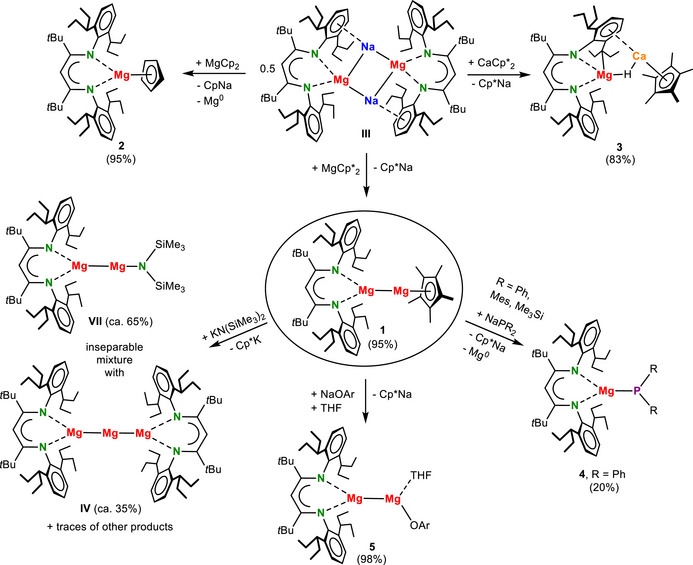
Synthesis and reactivity of (BDI*)MgMgCp* (**1**).

**Figure 1:**
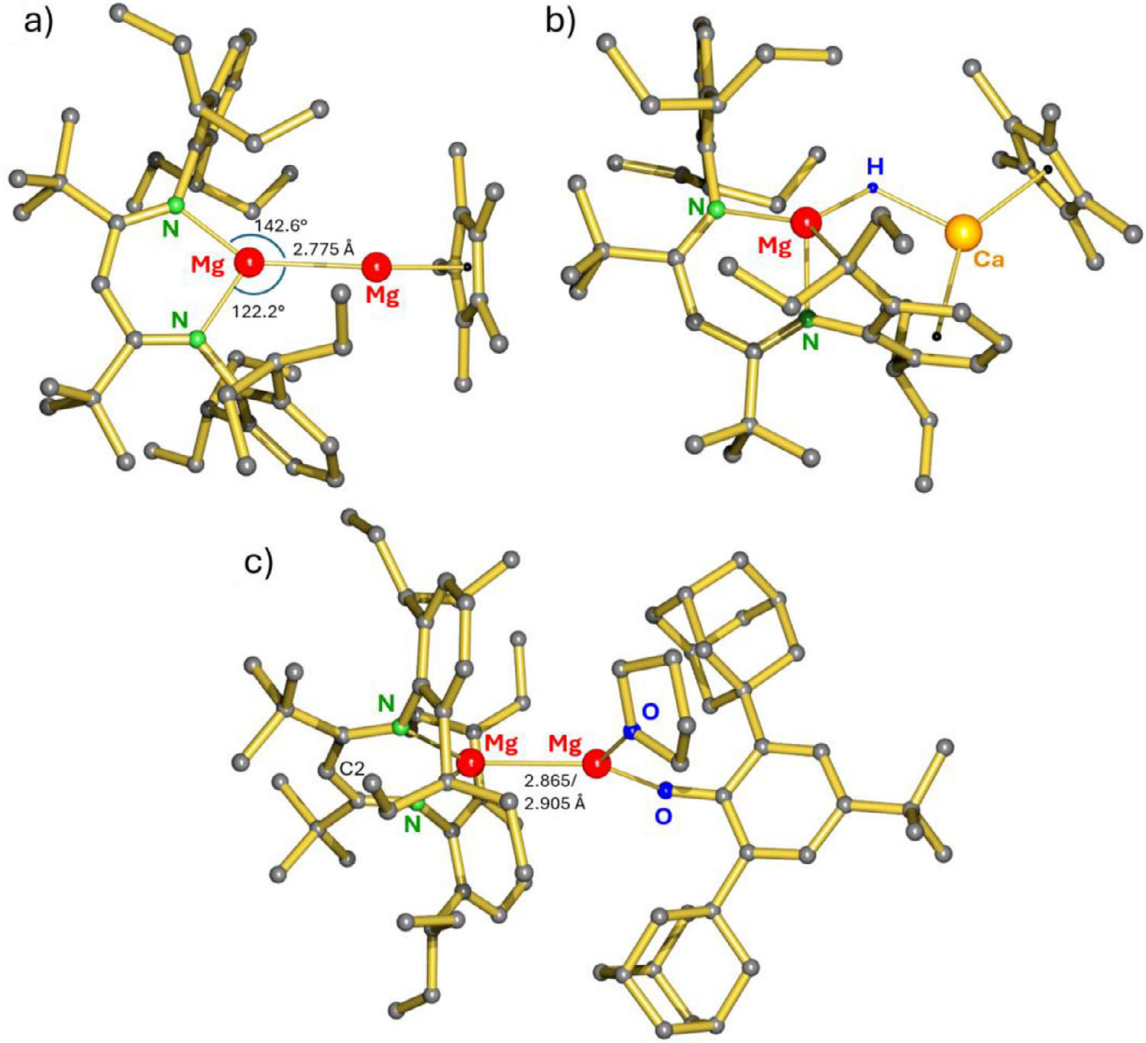
Crystal structures of a) (BDI*)MgMgCp* (**1**), b) a mixed Mg/Ca hydride complex (**3**) and c) (BDI*)MgMgOAr·THF (**5**). H atoms omitted for clarity.

Complex **1** represents the first Mg^I^ complex in which one of the Mg^I^ centres is not stabilized by a N‐ligand. Its stability originates from the superbulky BDI* ligand which allows use of a much smaller ligand at the second Mg^I^ centre. However, attempts to isolate the even smaller Cp‐analogue of **1** failed. Reaction of **III** with MgCp_2_ (Scheme 2) led to quantitative formation of (BDI*)MgCp (**2**) which is presumably formed by decomposition of an insufficiently sterically stabilized (BDI*)MgMgCp intermediate that disproportionated to **2** and atomic Mg^0^ (see Figure S37 for a crystal structure of **2**). Repeating the procedure at low temperature (−30 °C) retarded the conversion considerably and eventually also led to **2** and Mg^0^. This decomposition was calculated to be endothermic: (BDI*)MgMgCp → (BDI*)MgCp + Mg^0^, ΔH = +18.0 kcal mol^−1^ (Figure S46). However, if one considers the condensation enthalpy, Mg^0^(g) → Mg^0^(s) (−35.3 kcal mol^−1^),^[^ ^18^ ^]^ disproportionation to **2** and metallic Mg is exothermic by ΔH = −17.3 kcal mol^−1^. In contrast, the same decomposition of **1**, (BDI*)MgMgCp* → (BDI*)MgCp* + Mg^0^(s), is endothermic by ΔH = +6.8 kcal mol^−1^. This shows that use of the bulkier Cp* ligand is essential.

Wondering whether this approach also could be the key to the synthesis of an unsupported Mg─Ca bond, **III** was reacted with CaCp*_2_ (Scheme 2). This led to the selective formation of an orange‐red Mg/Ca hydride complex (**3**) in 85% yield. Formation of **3** likely proceeds through intermediate (BDI*)MgCaCp*, our target complex, which is formally a Mg^0^‐Ca^II^ species. This means that the Mg─Ca bond is strongly polarized (Mg^δ−^‐Ca^δ+^) which, as previously reported,^[^ ^19^ ^]^ results in high instability. In this particular case, the Mg^0^ centre inserts in a Et_2_C─H bond. Such C─H bond activation by oxidative addition is generally observed for Pd^0^ or Pt^0^ but only one example has been reported for zerovalent *s*‐block metals like Mg^0^.^[^ ^19^ ^]^ The crystal structure of **3** is the second example of a mixed Mg/Ca hydride complex (Figure 1b).^[^ ^20^ ^]^ The hydride atom in **3** was found and refined and Mg─H [1.80(3) Å] and Ca─H [2.15(3) Å] distances fall within the expected range for Mg and Ca hydride complexes.^[^ ^21^, ^22^ ^]^ However, the chemical shift for the hydride ligand (+2.04 ppm) is at unusually high field. Ca hydride complexes display lower field signals in the 3–5 ppm range.^[^ ^22^ ^]^ Although Mg hydride signals are generally found at higher field, there is only one example of a Mg hydride complex featuring lower ppm values of +1.72 and +0.55 ppm.^[^ ^23^ ^]^ A recently reported Ca hydride complex shows a hydride chemical shift of +2.0 ppm which compares well to that in **3**.^[^ ^24^ ^]^ This unusually low chemical shift was explained with a ring current effect of a benzene ligand. Similar ring currents of either the Cp* or the BDI* aryl substituent may explain the low chemical shift for the hydride ligand in **3**.

DFT calculations are in agreement with the difference in stability of (BDI*)MgMgCp*(**1**) and (BDI*)MgCaCp* (Figures 2a and S44). The reaction, 0.5 **III** + MgCp*_2_ → (BDI*)MgMgCp* + Cp*Na, is endothermic (ΔH = +22.0 kcal mol^−1^). However, if one considers the formation of insoluble coordination polymers, (Cp*Na)_n_, the reaction gradually becomes exothermic with increasing aggregation (*n* = 6, ΔH = −4.9 kcal mol^−1^); Figure S44. The same reaction with heavier metallocenes AeCp*_2_ (Ae = Ca, Sr, Ba) remains endothermic (Figure 2a). Failure to isolate (BDI*)MgCaCp* can also be explained with thermodynamically favourable decomposition by C─H activation (Figure 2b) which has an activation enthalpy of only +20.4 kcal mol^−1^ and is exothermic. In contrast, the activation enthalpy for decomposition of (BDI*)MgMgCp* (**1**) is much higher (ΔH = +37.6 kcal mol^−1^) and the reaction is essentially thermoneutral, explaining its thermal stability up to 120 °C. Decomposition of the heavier (BDI*)MgAeCp* complexes (Ae = Ca, Sr, Ba) follow similar transition states and energy profiles (Figure S45), indicating that also the Sr and Ba complexes will decompose by C─H activation.

**Figure 2:**
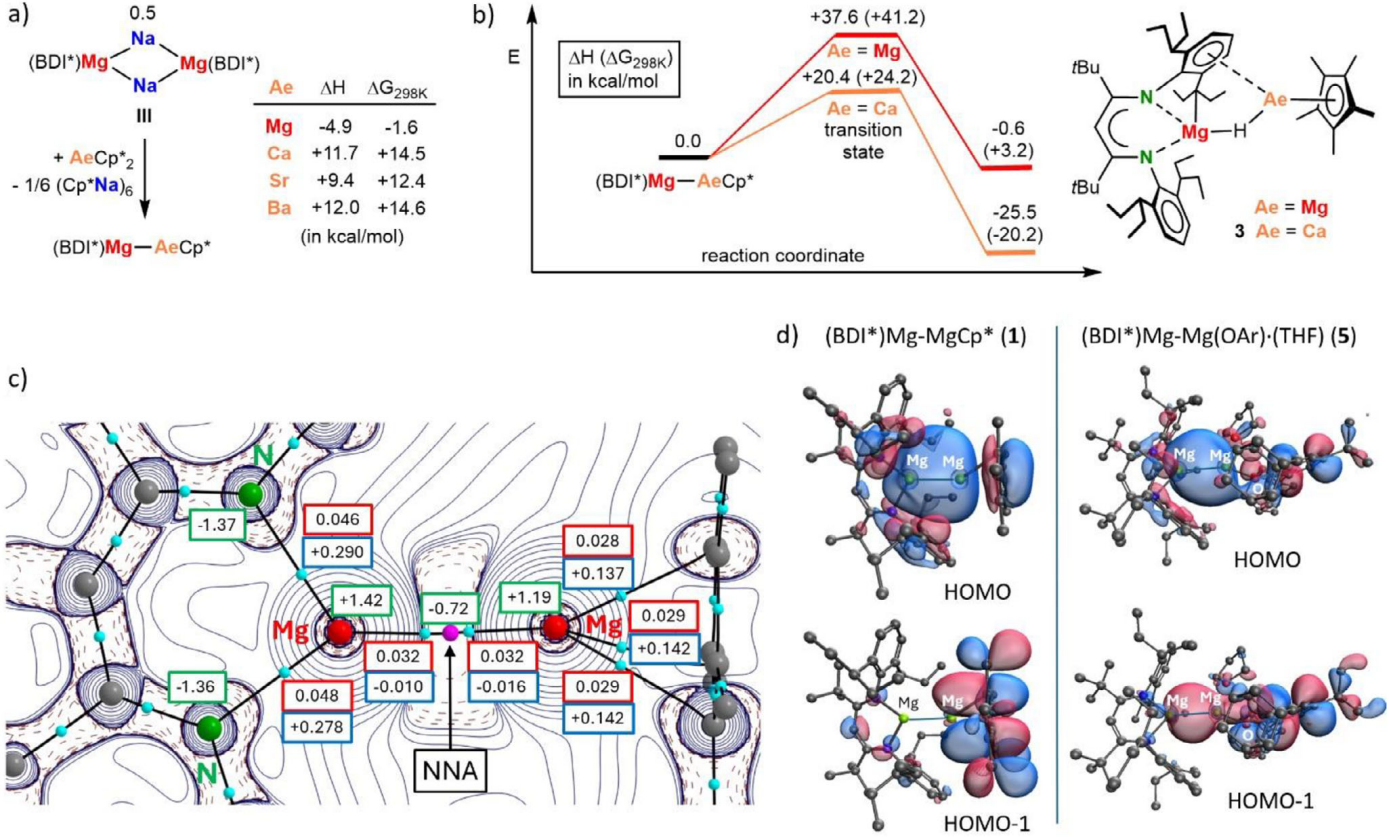
a) Thermodynamic evaluation for formation of (BDI*)MgAeCp* complexes. b) Energy profile for decomposition of (BDI*)MgAeCp* (Ae = Mg, Ca); ΔH (ΔG_298K_) in kcal mol^−1^. c) Atoms‐In‐Molecules analysis for (BDI*)MgMgCp* (**1**) showing the Laplacian distribution with bcp's as light‐blue dots and AIM charges in green boxes. The electron density *ρ*(r) in *e* Bˉ^3^ and Laplacian ∇^2^ *ρ*(r) in *e* Bˉ^5^ are shown in red and blue boxes, respectively. d) The HOMO and HOMO‐1 for (BDI*)MgMgCp* (**1**) and (BDI*)MgMg(OAr)·(THF) (**5**).

Natural‐Population‐Analysis (NPA) of **1** shows negative charges for the ligands (BDI*: ‐0.99, Cp*: ‐0.95) and positive charges at the Mg nuclei (BDI*‐Mg: +1.04, Cp*‐Mg: +0.89); Figure S48. This confirms its essentially ionic nature and indicates a slightly polarized (BDI*)Mg^δ+^─Mg^δ^¯(Cp*) bond. These charges also reflect the different electronic character of the two dissimilar ligands. Whereas the N‐based BDI* ligand is electronegative, the Cp* ligand is more electron donating. Atoms‐In‐Molecules (AIM) analysis shows a Non‐Nuclear‐Attractor (NNA) with a basin of 0.72*e* on the Mg‐Mg axis (Figure 2c). Hence, AIM calculated somewhat more positive charges on both Mg centres (BDI*‐Mg: +1.42, Cp*‐Mg: +1.19). Such NNAs are a common feature for the Mg^I^‐Mg^I^ bond^[^ ^25^ ^]^ and have recently also been found in a Be‐Al bond.^[^ ^26^ ^]^ The HOMO in **1** is essentially the bonding Mg(3*s*)─Mg(3*s*) interaction while the HOMO‐1 shows bonding Mg(3*p*)─Cp*(π*) interaction (Figure 2d).

A benzene solution of the asymmetric Mg^I^ complex (BDI*)MgMgCp* (**1**) is thermally highly stable. Even at temperatures up to at least 120 °C, no decomposition was observed. This high thermal stability is quite remarkable. Crimmin and coworkers found that a mixture of (BDI)MgMg(BDI) and (BDI’)MgMg(BDI’) complexes can exchange ligands giving rise to a statistical mixture with asymmetric (BDI)MgMg(BDI’).^[^ ^27^ ^]^ Comprehensive investigations by Stasch and coworkers^[^ ^28^ ^]^ are in favour of a BDI/BDI’ anion exchange mechanism rather than homolytic Mg‐Mg bond cleavage which has been shown to need UV or blue light.^[^ ^29^ ^]^ This first cyclopentadienyl Mg^I^ complex unlocks the potential to access a range of asymmetric (BDI*)MgMgL complexes by the salt‐metathesis route. Driven by the complete insolubility of (Cp*M)_n_ (M = Li, Na, K), it may be feasible to exchange the Cp* ligand simply by addition of LM reagents. Salt‐metathesis between (BDI*)MgMgCp* (**1**) and KN(SiMe_3_)_2_ led to formation of literature‐known (BDI*)MgMgN(SiMe_3_)_2_ (**VII**) and precipitation of Cp*K. ^1^H NMR analysis confirmed the Cp*K side‐product but also showed that the exchange reaction was only circa 65% selective. ^1^H and ^13^C NMR analysis indicate circa 35% of the side‐product (BDI*)MgMgMg(BDI*) (I**V**) (Figures S19–S22), which could not be separated and must have been formed during ligand exchange by disproportionation (2 Mg^I^ → Mg^0^ + Mg^II^) and Mg^0^ capture.^[^ ^13^ ^]^

Despite not being fully selective, the experiment demonstrated the Cp*/L exchange concept. Questioning whether the prototypical N‐ligands in Mg^I^ complexes may be substituted by P‐ligands, a solution of **1** in benzene/THF was reacted with NaPPh_2_. There was no clear colour change but a grey precipitate started to separate from the yellow solution. A THF extract of this precipitate contains Cp*Na while the remaining insoluble grey fraction is likely Mg^0^. The remaining yellow solution was fully dried and crystallization of the raw product from Et_2_O gave crystals of (BDI*)MgPPh_2_·Et_2_O (**4**‐Et_2_O) which was fully characterized (crystal structure: Figure S39; also the THF adduct **4**‐THF was structurally characterized: Figure S40). These observations indicate the initial formation of (BDI*)MgMgPPh_2_ which disproportionated to **4** and Mg^0^(s). This is in agreement with calculations indicating that this decomposition is highly exothermic (ΔH = ‐33.1 kcal mol^−1^, Figure S47). Similar experiments with bulkier phosphides, like NaPMes_2_ or LiP(SiMe_3_)_2_, also led to Mg^0^ precipitation. In the latter case, (BDI*)MgP(SiMe_3_)_2_ was identified by X‐ray diffraction (Figure S41). Reaction of **1** with LiP(SiMe_3_)_2_ in benzene led to isolation of (BDI*)Li as a side‐product which crystallized in presence of THF as (BDI*)Li·(THF) (Figure S42), showing that exchange of the BDI* ligand can be an issue.

As softer P‐ligands may not be suitable to stabilize Mg^I^ complexes, a solution of **1** in benzene/THF was reacted with a NaOAr reagent (Scheme 2). For complex stability we chose the very bulky 2,5‐adamantyl‐3‐*t*Bu‐phenoxide anion. After separation of the Cp*Na precipitate and all solvents the essentially pure raw product (BDI*)MgMgOAr·THF (**5**) could be isolated in 98% yield (Figure S30). As reactions in pure benzene were much less selective, the THF ligand has a stabilizing function. In an attempt to synthesize **5** by an alternative route, complex **1** was reacted with one equivalent of ArOH. However, instead of Cp* protonation and aryloxide formation, the Mg^I^ complex reduced the acidic reagent to ArO¯ and H_2_, the latter being visible by gas evolution. A similar reactivity was observed in the reaction of a (BDI)MgMg(BDI) reagent with acidic NH_3_BH_3_.^[^ ^30^ ^]^ This stands in strong contrast with the redox stability of Cp*ZnZnCp* (**I**) in which the Cp* ligands can be exchanged by acidic reagents under retention of the Zn─Zn bond.^[^ ^31^, ^32^ ^]^

The crystal structure of (BDI*)MgMgOAr·THF (**5**) shows two independent molecules with similar structures in the asymmetric unit. The Mg─Mg bonds of 2.865(2)/2.905(2) Å are in the normal range for less sterically congested Mg^I^ complexes. In both independent molecules, the Mg‐Mg‐O(Ar) angles [143.7(1)/146.0(1)°] indicate significant bending which is imposed by Mg‐THF coordination. The calculated structure without THF is strictly linear. More striking is the bending of the Mg‐O‐C(Ar) units [131.5(2)/139.6(2)°]. Terminal Mg─O─Ar′ bonds (Ar′ = 2,6‐*t*Bu‐phenolate) are in general near to linear. This arrangement may be reinforced by bulky *ortho*‐substituents. *Cf*. Mg‐O‐C angles of 171.0(1)° in (Ar'O)_2_Mg·(THF)_2_ or 178.4(3)° in the Mg(OAr′)_3_¯ anion have been published (Ar′ = bulky aryl).^[^ ^33^, ^34^ ^]^ However, a report of a Mg‐O‐C(Ar′) angle of 156.0(2)° shows that deviations from this preferred linear Mg‐O‐Ar′ arrangement are possible.^[^ ^35^ ^]^ The small Mg‐O‐C(Ar) angles in **5** may have its origin in Mg(3*s*)‐OAr(π*) overlap in the HOMO‐1 (*vide infra*).

NPA shows negative charges for the ligands (BDI*: ‐1.00, OAr: ‐0.96) and positive charges at the Mg nuclei (BDI*‐Mg: +0.94, ArO‐Mg: +1.02); Figure S48. Therefore, the Mg‐Mg bond in **5** is less polarized than that in **1**. AIM analysis (Figure S50) also shows very similar charges on both Mg nuclei which are substantially more positive due to the location of an NNA with a basin of 0.78*e* on the middle of the Mg‐Mg axis. The HOMO in **5** represents the usual Mg(3*s*)‐Mg(3*s*) orbital overlap but also shows some bonding Mg(3 *s*)‐OAr(π*) interaction (Figure 2d). The latter Mg(3*s*)‐OAr(π*) overlap is even more pronounced in the HOMO‐1. It seems that the electron‐rich Mg^I^ centres are stabilized by transferring some of its electron density to the OAr(π*)‐orbital.

After nearly two decades of Mg^I^ chemistry, we here describe the first Mg^I^ centres with non‐N ligands. This chemistry is enabled by a recently reported Na magnesyl reagent (**III**), allowing the creation of new Mg─metal bonds by salt‐metathesis. Thus, the unique (BDI*)MgMgCp* (**1**) was isolated in near quantitative yield. Access to a Mg^I^ complex with a Cp‐type ligand is the key to the synthesis of a first Mg^I^ aryloxide complex. Salt‐metathesis between **1** and NaOAr led to precipitation of Cp*Na and quantitative formation of (BDI*)MgMgOAr·THF (**5**). This stands in stark contrast with anionic ligand exchange reactions between different (BDI)MgMg(BDI) complexes which generally result in statistical product mixtures.^[^ ^27^, ^28^ ^]^

Addition of an alkali metal complex to a cyclopentadienyl magnesium(I) complex represents a simple modular approach to access a large diversity of asymmetric (BDI*)MgMgL complexes. As polarization of the Mg‐Mg bond results in much higher reactivity,^[^ ^36^ ^]^ this concept is a desired extension in Mg^I^ chemistry. Whether asymmetric Mg^I^ dimers can be isolated depends on product stability and the selectivity of the exchange reaction. While there are several methods to exchange the ligands in Zn^I^ dimers like **I**,^[^ ^31^, ^32^, ^37^ ^]^ this is the first synthetic method for facile ligand exchange at Mg^I^ dimers. We currently investigate the extended scope and limitations of this concept and aim to expand this chemistry to symmetric Mg^I^ cyclopentadienyl complexes.

## Supporting Information

Complex syntheses and analyses, selected NMR spectra, crystallographic details, including ORTEP representations, details for the DFT calculations including XYZ‐files. Deposition Numbers  2477181 (for (BDI*)MgMgCp*, **1**), 2477182 (for (BDI*‐H)Mg(H)CaCp*, **3**), 2477183 (for (BDI*)MgCp, **2**), 2477184 (for [(BDI*)MgPPh_2_·Et_2_O, **4**‐Et_2_O), 2477185 (for (BDI*)MgPPh_2_·THF, **4**‐THF), 2477186 (for (BDI*)MgP(SiMe_3_)_2_), 2477187 (for (BDI*)MgMgOAr·THF, **5**) and 2477188 (for (BDI*)Li·THF) contain the supplementary crystallographic data for this study. These data are provided free of charge by the joint Cambridge Crystallographic Data Centre and Fachinformationszentrum Karlsruhe http://www.ccdc.cam.ac.uk/structures Access Structures service.

## Conflict of Interests

The authors declare no conflict of interest.

## Supporting information



Supporting Information

## Data Availability

The data that support the findings of this study are available in the supplementary material of this article.
