# Supplementary material for: A Modular Approach to Asymmetric Mg(I) Complexes Using a Unique Cyclopentadienyl Mg(I) Complex
Source: Angew Chem Int Ed Engl. 2025 Oct 17;64(50):e202518538. doi: 10.1002/anie.202518538 (PMC12684354; doi:10.1002/anie.202518538)
Supplement: Supplementary file 1 — Supporting Information [file ANIE-64-e202518538-s001.pdf]

Supporting Information

Table of Contents

1. Materials and methods S2
2. Complex synthesis S3
3. NMR Characterization S8
4. Crystal structure determinations S28
5. DFT Calculation S38
6. References S82

1. Materials and methods

All experiments were conducted in dry glassware under an inert nitrogen atmosphere by applying standard Schlenk techniques or gloveboxes (MBraun) using freshly dried and degassed solvents. All solvents were degassed with nitrogen, dried over activated aluminum oxide (Innovative Technology, Pure Solv 400-4-MD, Solvent Purification System), and then stored under inert atmosphere over molecular sieves (3 Å) unless noted otherwise. Deuterated benzene (C_6D_6), deuterated THF (THF-d_8), methylcyclohexane-d_{14} and cylcohexane-d_{12} were purchased from Sigma Aldrich or Deutero GmbH, degassed and dried over molecular sieves (3 Å). 2,6-($CHEt_2$)-aniline,[S1] $MgCp^*_2$[S2] and $(Me_3Si)_2PLi(THF)_2$[S3] were synthesized according to slightly modified literature procedures. The following compounds were synthesized according to literature procedures: (^{DIPeP}BDI*)H,[S4] (^{DIPeP}BDI*)MgI,[S4] [(^{DIPeP}BDI*)MgNa]$_2$,[S5] $CaCp^*_2$,[S6] $MgCp_2$,[S7] HOAr^{Ad2tBu},[S8] NaOAr^{Ad2tBu},[S9] $NaPPh_2$.[S10] $KN(SiMe_3)_2$ was purchased from Sigma-Aldrich (95%) and was used without further purification; HOAr^{Ad2tBu} = 2,5-adamantyl-3-*t*Bu-phenol.

NMR spectra were measured on Bruker Avance III HD 400 MHz and Bruker Avance III HD 600 MHz spectrometers. Chemical shifts (δ) are denoted in ppm (parts per million), coupling constants in Hz (Hertz). For describing signal multiplicities common abbreviations are used: s (singlet), d (doublet), t (triplet), q (quartet), p (quintet), sept (septet), m (multiplet) and br (broad). Spectra were referenced to the solvent residual signal. Assignments of resonance signals in the ^1H and $^{13}C\{^1H\}$ NMR spectra were made based on two-dimensional NMR correlation (HSQC, HMBC, COSY) experiments. Elemental analysis was performed with an Hekatech Eurovector EA3000 analyzer.

2. Complex synthesis

Synthesis of (BDI*)MgMgCp*(1)

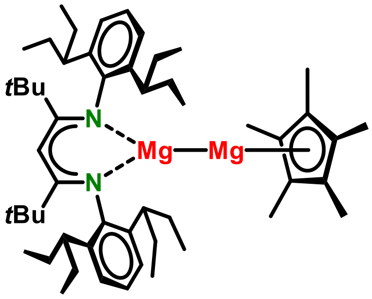

[(BDI*)MgNa]$_2$ (32.6 mg, 24.6 μmol) was suspended in C_6D_6 (400 μL) and THF-d_8 (200 μL) and MgCp*$_2$ (14.5 mg, 49.2 μmol) was added. The resulting brown suspension was stirred for 2 hours at room temperature, filtered and all volatiles were removed *in vacuo*. The light brown solid was stripped with pentane (1 mL) to obtain essentially pure **1** (37.1 mg, 46.5 μmol, 95%) as orange-brown powder. Yellow crystals, suitable for single crystal X-ray diffraction, were obtained by storing a concentrated solution of **1** in hexanes at -35 °C.

^{1}H NMR (600.13 MHz, C_6D_6, 298 K): δ = 7.03–6.99 (m, 2H, para-Ar**H**), 6.96–6.93 (m, 4H, meta-Ar**H**), 5.26 (s, 1H, C**H**-backbone), 2.93 (m, 4H, C**H**Et$_2$), 2.01 (s, 15H, Cp*C**H**$_3$), 1.83–1.65 (m, 16H, C**H**$_2$CH$_3$), 1.18 (s, 18H, *t*Bu**H**), 0.99–0.92 (m, 24H, CH$_2$C**H**$_3$) ppm.

^{13}C{^{1}H}-NMR (152.92 MHz, C_6D_6, 298 K): δ = 175.2 (*t*Bu-**C**), 147.1,138.5 (2 signals, Ar**C**), 128.4, 125.7, 123.2 (3 signals, ArCH), 109.7 (Cp***C**), 95.8 (**C**H-backbone), 43.7 (*t*Bu**C**), 40.8 (**C**HEt$_2$), 33.1 (*t*Bu**C**H$_3$), 25.7, 24.8 (2 signals, **C**H$_2$CH$_3$), 11.9 (CH$_2$**C**H$_3$), 10.7, 10.4 (2 signals, Cp***C**H$_3$) ppm.

Elemental analysis. Calculated for $C_{53}H_{84}Mg_2N_2$ (M = 797.88 g/mol): C 79.78, H 10.61, N 3.51 %. Found: C 79.48, H 10.58, N 3.59 %.

Synthesis of (BDI*)MgCp (2)

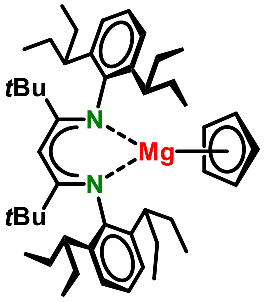

[(BDI*)MgNa]$_2$ (84.0 mg, 63.5 µmol) was suspended in C_6D_6 (500 µL) and $MgCp_2$ (19.6 mg, 127 µmol) was added. During stirring, the color changed from dark brown to reddish brown. After one hour, the suspension was filtered and all volatiles were removed in vacuo. The red-brown solid was stripped with pentane (1 mL) to obtain essentially pure **2** (85.0 mg, 121 µmol, 95%) as red-brown powder. Pale brown crystals, suitable for single crystal X-ray diffraction, were obtained by storing a concentrated solution of **2** in pentane at -35 °C.

^{1}H NMR (600.13 MHz, C_6D_6, 298.0 K): δ = 7.04–7.00 (m, 2H, para-Ar**H**), 6.97–6.94 (m, 4H, meta-Ar**H**), 5.96 (s, 5H, Cp**H**), 5.30 (s, 1H, backbone C**H**), 2.94-2.89 (m, 4H, C**H**Et$_2$), 1.82–1.65 (m, 16H, C**H$_2$**CH$_3$), 1.17 (s, 18H, *t*Bu**H**), 0.97 (t, $^3J_{HH}$ = 7.4 Hz, 12H, CH$_2$C**H$_3$**), 0.93 (t, $^3J_{HH}$ = 7.4 Hz, 12H, CH$_2$C**H$_3$**) ppm.

^{13}C{^{1}H}-NMR (150.92 MHz, C_6D_6, 298.0 K): δ = 175.6(*t*Bu-**C**), 147.1,138.4 (2 signals, Ar**C**), 125.7 (meta-Ar**C**H), 123.3 (para-Ar**C**H), 105.0 (Cp**C**H), 96.1 (**C**H-backbone), 43.8 (*t*Bu**C**), 41.0 (**C**HEt$_2$), 33.1 (*t*Bu**C**H$_3$), 26.0, 24.8 (2 signals, **C**H$_2$CH$_3$), 11.9, 10.6 (2 signals, CH$_2$**C**H$_3$) ppm.

Elemental analysis. Calculated for $C_{48}H_{74}MgN_2$ (M = 702.57 g/mol): C 81.96, H 10.60, N 3.98 %. Found: C 80.01, H 10.37, N 3.77 %.

Synthesis of (BDI*-H)Mg(H)CaCp* (3)

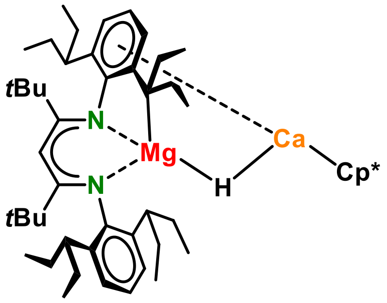

[(BDI*)MgNa]$_2$ (25.0 mg, 18.9 µmol) and CaCp*$_2$ (11.7 mg, 37.7 µmol) were dissolved in cyclohexane-d$_{12}$ (600 µL) and the black reaction mixture was stirred for 2 d at room temperature. The dark red suspension was filtered and the filtrate was evaporated to dryness and stripped with pentane (3x5mL) to obtain essentially pure (**3**) as orange-red powder (30.4 mg, 31.3 µmol, 83%). Bright red crystals suitable for single crystal X-ray diffraction were obtained by storing a saturated solution of **3** in hexanes at –30 °C.

^{1}H-NMR (600.13 MHz, cyclohexane-d$_{12}$, 298 K): δ = 7.05–7.02 (m, 1H, Ar**H**), 6.91–6.85 (m, 3H, Ar**H**), 6.55–6.51 (m, 2H, Ar**H**), 5.06 (s, 1H, C**H**-backbone), 2.97–2.86 (m, 3H, C**H**Et$_2$), 2.07–1.92 (m, 5H, C**H**$_2$CH$_3$), 2.04 (s, 1H, Ca-**H**-Mg), 1.90–1.84 (m, 2H, C**H**$_2$CH$_3$), 1.82 (s, 15H, Cp*C**H**$_3$), 1.80–1.64 (m, 6H, C**H**$_2$CH$_3$), 1.63–1.53 (m, 3H, C**H**$_2$CH$_3$), 1.14 (s, 9H, tBu**H**), 1.09 (t, $^3J_{H,H}$ = 7.2 Hz, 3H, CH$_2$C**H**$_3$), 1.05 (t, superimposed, 3H, CH$_2$C**H**$_3$), 1.03 (s, 9H, *t*Bu**H**), 1.00–0.96 (m, 6H, CH$_2$C**H**$_3$), 0.89 (t, $^3J_{H,H}$ = 7.2 Hz, 3H, CH$_2$C**H**$_3$), 0.81–0.77 (m, 6H, CH$_2$C**H**$_3$), 0.70 (t, $^3J_{H,H}$ = 7.4 Hz, 3H, CH$_2$C**H**$_3$) ppm.

^{13}C{^{1}H}-NMR (150.92 MHz, cyclohexane-d$_{12}$, 298 K): δ = 177.6, 175.2 (2 signals: tBu-**C**), 152.0, 151.4, 148.2, 140.5, 139.2, 138.0 (6 signals, Ar**C**), 126.1, 125.3, 123.7, 123.2, 122.2 (5 signals, ArCH), 114.9 (Cp***C**), 113.3 (1C, ArC**H**), 95.5 (**C**H-backbone), 56.9 (Et$_2$**C**-Mg), 44.3, 43.9 (2 signals, *t*Bu**C**), 41.94, 41.91, 39.9 (3 signals, **C**HEt$_2$), 33.5, 32.1 (2 signals, *t*Bu**C**H$_3$), 31.4, 31.3, 29.6, 27.5, 27.4, 25.1, 24.5, 23.5 (8 signals, **C**H$_2$CH$_3$), 15.2, 14.1, 13.4, 12.9, 12.5, 12.3 (6 signals, CH$_2$**C**H$_3$), 11.2 (Cp***C**H$_3$), 10.9, 8.8 (2 signals, CH$_2$**C**H$_3$) ppm.

Elemental analysis. Calculated for $C_{53}H_{84}CaMgN_2$ (M = 813.65 g/mol): N 3.44, C 78.24, H 10.41 %. Found: N 3.89, C 78.26, H 10.91 %.

Synthesis of (BDI*)MgPPh$_2$ (4)

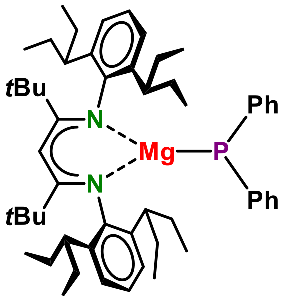

(BDI*)MgMgCp* (96.8 mg, 121 µmol) was suspended in benzene (500 µL) and NaPPh$_2$(THF) (34.0 mg, 121 µmol) in THF (200 µL) was added. The yellow solution was stirred for 7 days at room temperature. During the reaction a grey precipitate was formed, which was filtered off. After all volatiles were removed in vacuo, the yellow solid was dissolved in Et$_2$O, filtered and stored at - 35 °C. After two weeks, yellow crystals of **4** were isolated by decanting, washed with cold pentane (−20 °C, 2 × 0.5 ml) and dried under vacuum (20 mg, 24.3 µmol, 20 %).

^{1}H NMR (600.13 MHz, C_6D_6, 298.0 K): δ = 7.10–7.07 (m, 2H, para-Ar**H**), 7.03–6.99 (m, 2H, meta-Ar**H**), 6.89–6.87 (m, 6H, PPh**H**), 6.67–6.63 (m, 4H, PPh**H**), 5.47 (s, 1H, C**H**-backbone), 3.06-3.02 (m, 4H, C**H**Et$_2$), 1.86–1.75 (m, 8H, C**H**Et$_2$), 1.68–1.61 (m, 4H, C**H**Et$_2$), 1.58–1.50 (m, 4H, C**H**Et$_2$), 1.13 (s, 18H, *t*Bu**H**), 0.95 (t, $^{3}J_{HH}$ = 7.4 Hz, 12H, CH$_2$C**H**$_3$), 0.87 (t, $^{3}J_{HH}$ = 7.3 Hz, 12H, CH$_2$C**H**$_3$) ppm.

^{13}C{^{1}H}-NMR (150.92 MHz, C_6D_6, 298.0 K): δ = 177.8 (tBu-**C**), 146.3 (Ar**C**), 143.0 (d, $^{1}J_{CP}$ = 20.3 Hz, PPh**C**), 140.0 (Ar**C**), 134.0 (d, $^{2}J_{CP}$ = 16.9 Hz, PPh**C**H), 126.3 (meta-Ar**C**H), 124.7 (para-Ar**C**H), 123.9 (PPh**C**H), 97.1 (C**H**-backbone), 44.3 (tBu**C**), 41.2 (**C**HEt$_2$), 33.0 (tBu**C**H$_3$), 27.11, 24.0 (2 signals, **C**H$_2$CH$_3$), 11.6, 10.5 (2 signals, CH$_2$**C**H$_3$) ppm.

^{31}P NMR (242.92 MHz, C6D6, 298.0 K): δ = -46.1 ppm.

Elemental analysis. Calculated for $C_{55}H_{79}MgN_2P$ (M = 822.53 g/mol): C 80.22, H 9.62, N 3.40 %. Found: C 80.08, H 9.70 , N 3.55 %.

Reaction of (BDI*)MgMgCp* with KN(SiMe$_3$)$_2$

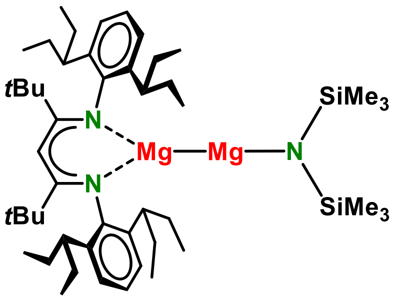

(BDI*)MgMgCp* (20.0 mg, 25.1 µmol) was dissolved in methylcyclohexane-d$_{14}$ (500 µL) and KN'' (5.00 mg, 25.1 µmol) was added. The orange suspension was stirred for 4 days at room temperature. The resulting brown suspension was filtered and the brown solid was isolated and dried under high vacuum. NMR analysis showed that the precipitate is Cp*K. The mother liquor contained circa 65% (BDI*)MgMgN'' and 35% of the side-product (BDI*)MgMgMg(BDI*), two complexes which both have been fully characterized.[S5] This was confirmed by comparison of ^{1}H and ^{13}C NMR signals.

Reaction of (BDI*)MgMgCp* with LiP(SiMe$_3$)$_2$

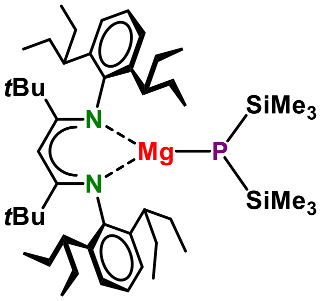

(BDI*)MgMgCp* (45 mg, 56.4 µmol) was suspended in benzene (500 µL) and LiP(SiMe$_3$)$_2$(2x THF) (18.5 mg, 56.4 µmol) was added. The yellow solution was stirred for 4 days at room temperature. During the reaction a grey precipitate was formed, which was filtered off. After all volatiles were removed in vacuo, the yellow solid was dissolved in hexane, filtered and a drop of THF was added. Storing the solution at -35 °C gave after

three weeks a mixture of yellow crystals which were identified by X-ray diffraction analysis as (BDI*)MgP($SiMe_3$)$_2$ and (BDI*)Li(THF).

Synthesis of (BDI*)MgMgOAr·THF (5)

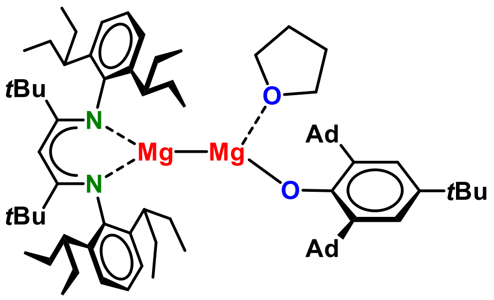

(BDI*)MgMgCp* (58.0 mg, 60.2 μmol) was dissolved in benzene (500 μL) and NaOAr (32.0 mg, 72.7 μmol) in THF (200 μL) was added. The yellow solution was stirred for 5 days at room temperature. During the reaction a white precipitate of NaCp* was formed, which was filtered off. All volatiles were removed in vacuo, and the yellow solid was stripped with pentane (1 mL) to obtain essentially pure **5** (63.6 mg, 59.0 μmol, 98 %) as yellow powder. Yellow crystals, suitable for single crystal X-ray diffraction, were obtained by storing a concentrated solution of **5** in methylcyclohexane with one drop of THF at -35 °C.

^{1}H NMR (600.13 MHz, C_6D_6, 298 K): δ = 7.39 (s, 2H, OAr**H**), 6.93–6.90 (m, 4H, meta-Ar**H**), 6.86–6.82 (m, 2H, para-Ar**H**), 5.39 (s, 1H, C**H**-backbone),3.26–3.17 (m, 4H, THF), 3.04–2.99 (m, 4H, C**H**Et$_2$), 2.36–2.32 (m, 12H, AdC**H**$_2$), 2.16–2.13 (m, 6H, AdC**H**), 2.03–1.98 (m, 4H, C**H**$_2$CH$_3$), 1.89–1.82 (m, 20H, AdC**H**$_2$/ C**H**$_2$CH$_3$), 1.73–1.68 (m, 8H, C**H**$_2$CH$_3$/THF), 1.50 (s, 9H, Ar*t*Bu**H**), 1.21 (s, 18H, *t*Bu**H**), 1.02–0.98 (m, 24H, CH$_2$C**H**$_3$) ppm.

^{13}C{^{1}H}-NMR (152.92 MHz, C_6D_6, 298 K): δ = 175.1 (*t*Bu-**C**), 161.4 (OAr**C**), 148.2, 139.5 (2 signals, Ar**C**), 136.0 (OAr**C**) 134.0 (*t*Bu-**C**Ar), 125.3 (meta-Ar**C**H), 123.3 (para-Ar**C**H), 121.3 (OAr**C**H), 96.3 (**C**H-backbone), 86.5 (THF), 44.0 (*t*Bu**C**), 42.3 (Ad**C**H$_2$), 40.8 (**C**HEt$_2$), 38.4 (Ad**C**H$_2$), 38.1 (Ad**C**), 34.7 (OAr*t*Bu**C**) 33.2 (*t*Bu**C**H$_3$), 32.6 (OAr*t*Bu**C**H$_3$), 30.1 (Ad**C**H), 27.5 (**C**H$_2$CH$_3$), 25.3 (THF), 25.2 (**C**H$_2$CH$_3$), 12.2, 11,2 (2 signals, CH$_2$**C**H$_3$) ppm.

Elemental analysis. Calculated for $C_{77}H_{118}Mg_2N_2O_2$ (M = 1152.41 g/mol): C 80.25, H 10.32, N 2.43 %. Found: C 80.99, H 10.49, N 2.06 %.

3. NMR Characterization

The residual solvent signals have been labeled as C6D6 for deuterated benzene and Cy for deuterated cyclohexane.

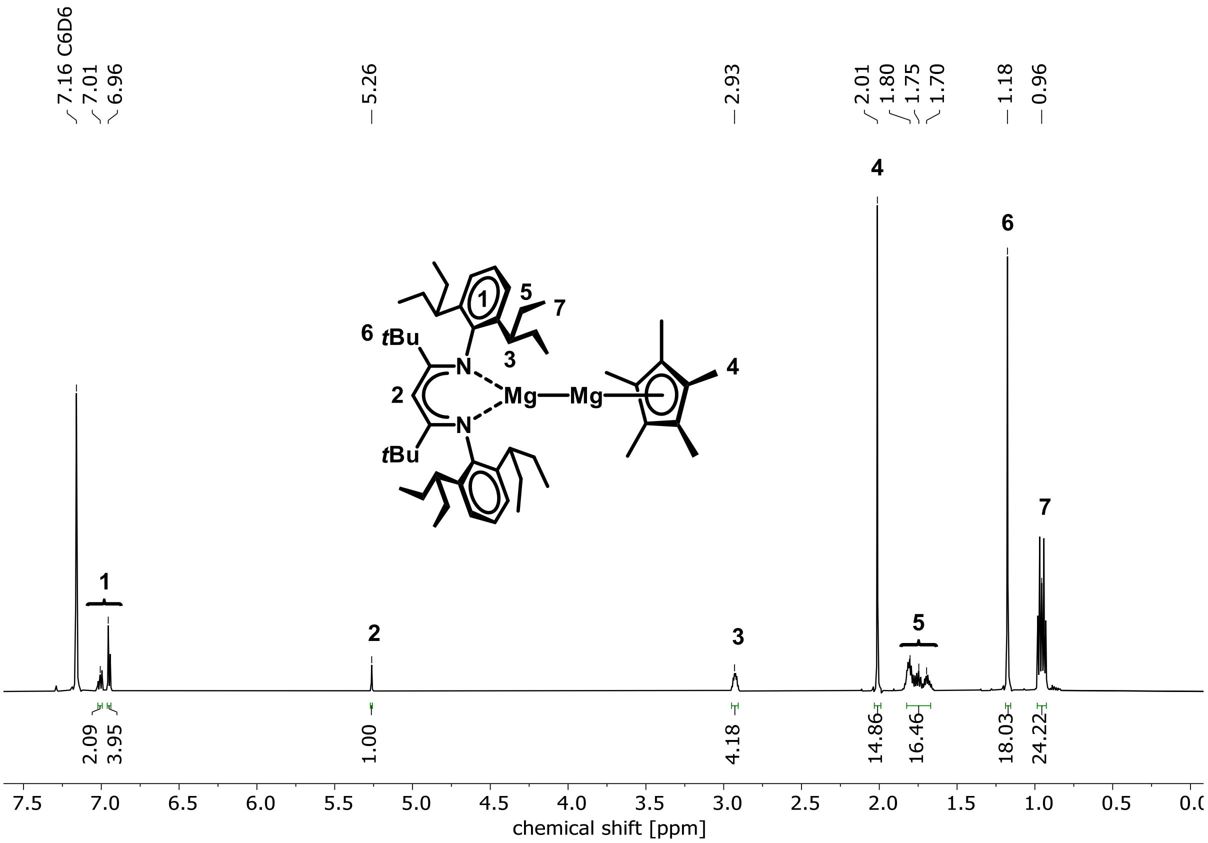

Figure S1. ^{1}H NMR spectrum (600.13 MHz, 298 K, C_6D_6) of the raw product of (BDI*)MgMgCp* (**1**).

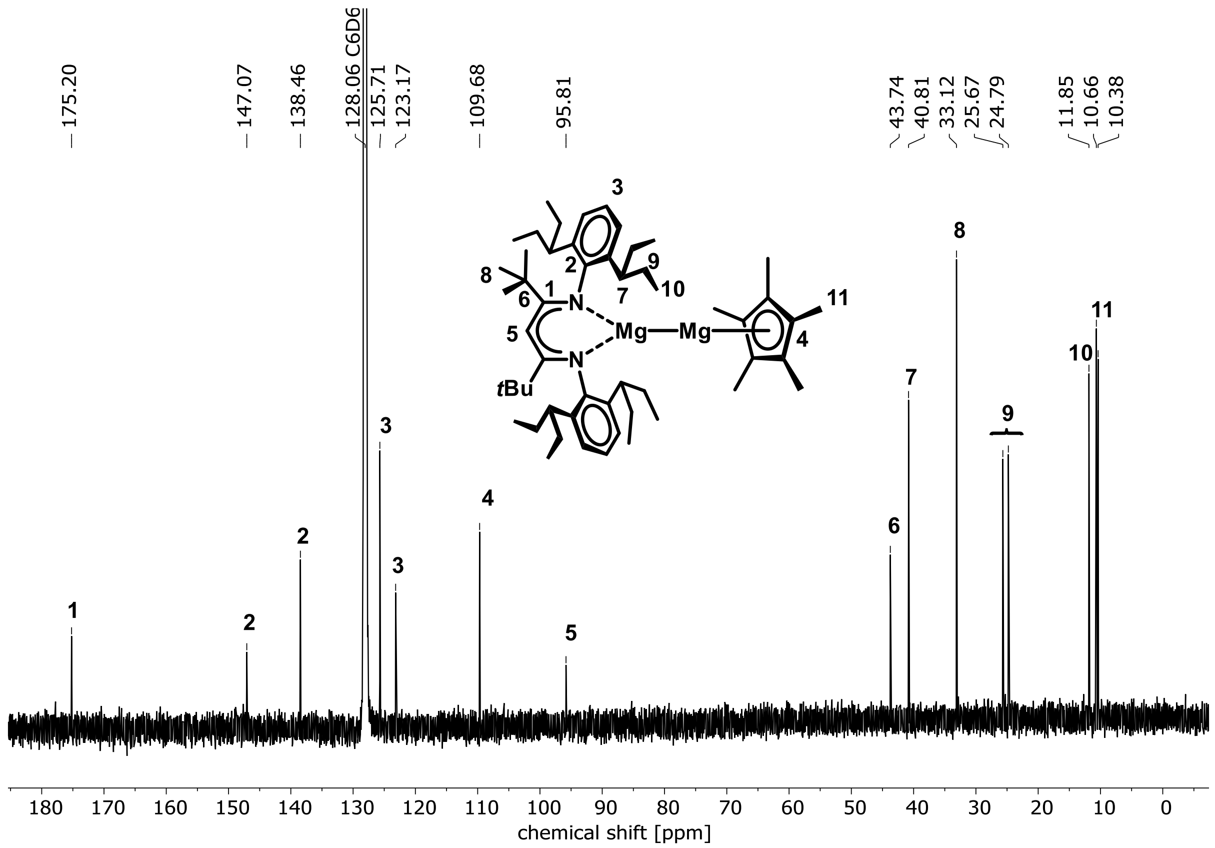

Figure S2. ^{13}C {^{1}H} NMR spectrum (151 MHz, 298 K, C_6D_6) of (BDI*)MgMgCp*(**1**).

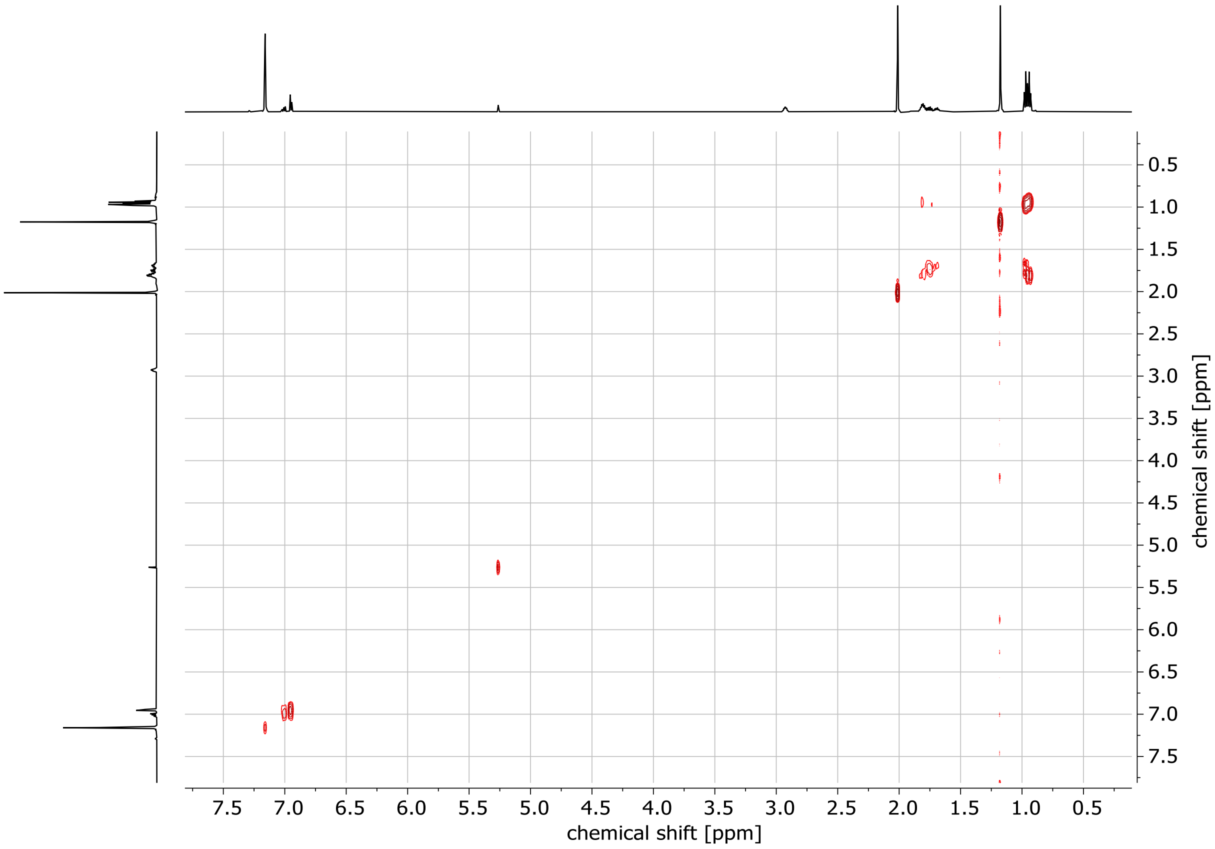

Figure S3. 2D-COSY NMR spectrum (600.13 MHz, 298 K, C_6D_6) of (BDI*)MgMgCp*(**1**).

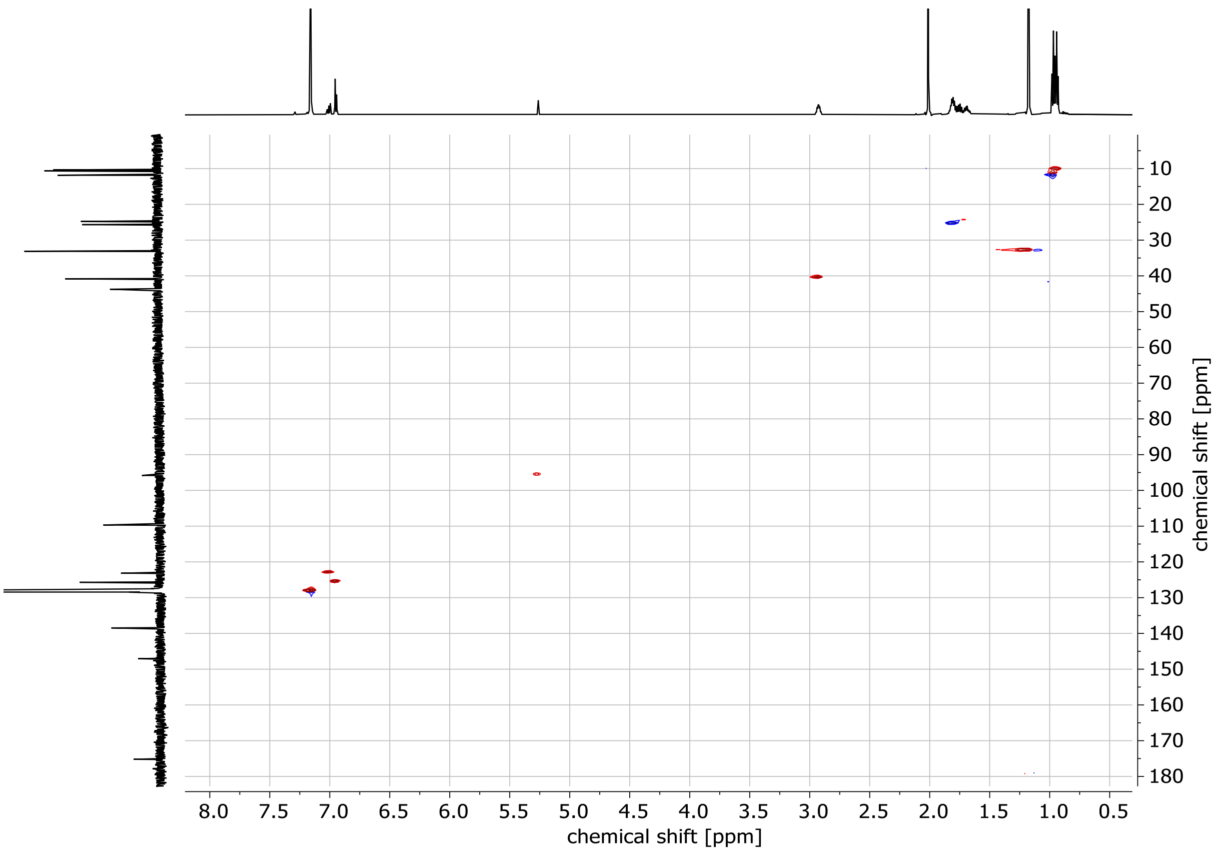

Figure S4. 2D-HSQC NMR spectrum (298 K, C_6D_6) of (BDI*)MgMgCp*(**1**).

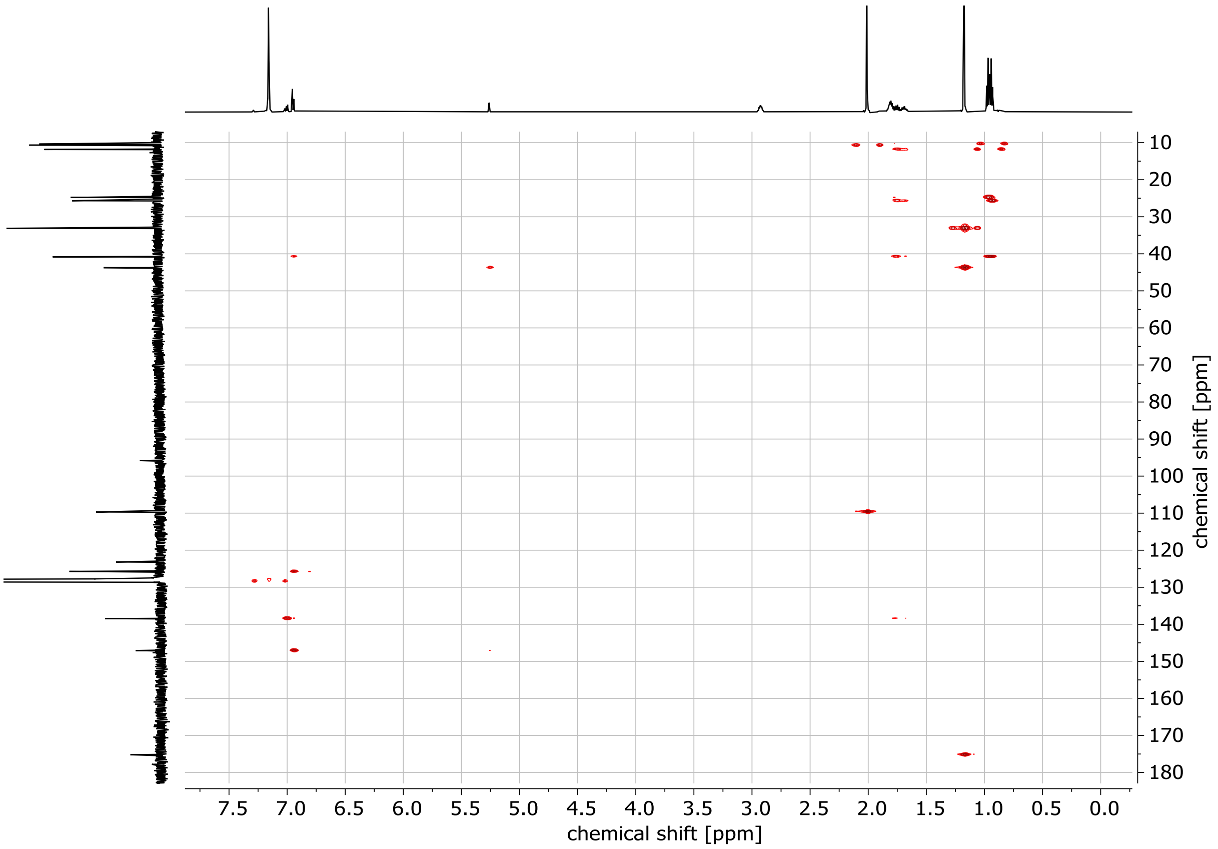

Figure S5. 2D-HMBC NMR spectrum (298 K, C_6D_6) of (BDI*)MgMgCp* (**1**).

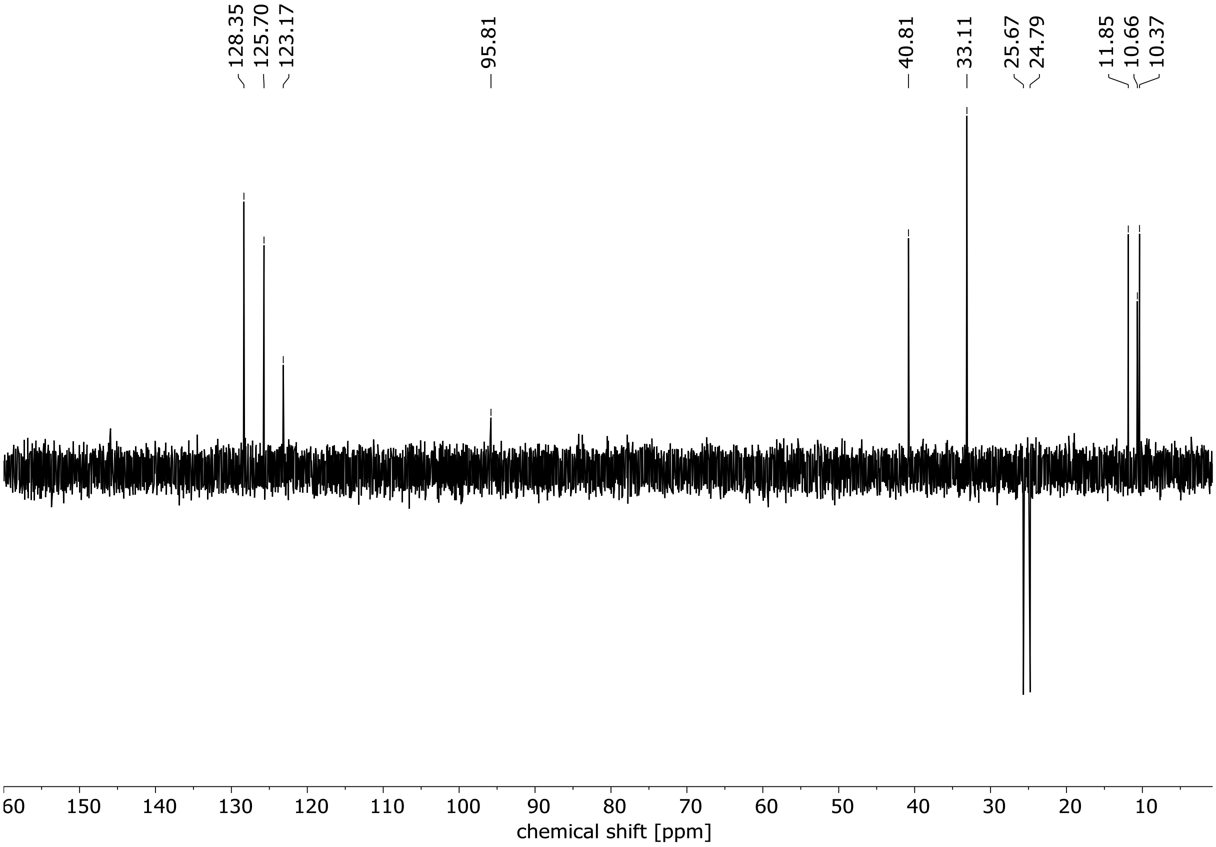

Figure S6. ^{13}C (DEPT 135) NMR spectrum (150.91 MHz, 298 K, C_6D_6) of (BDI*)MgMgCp* (**1**).

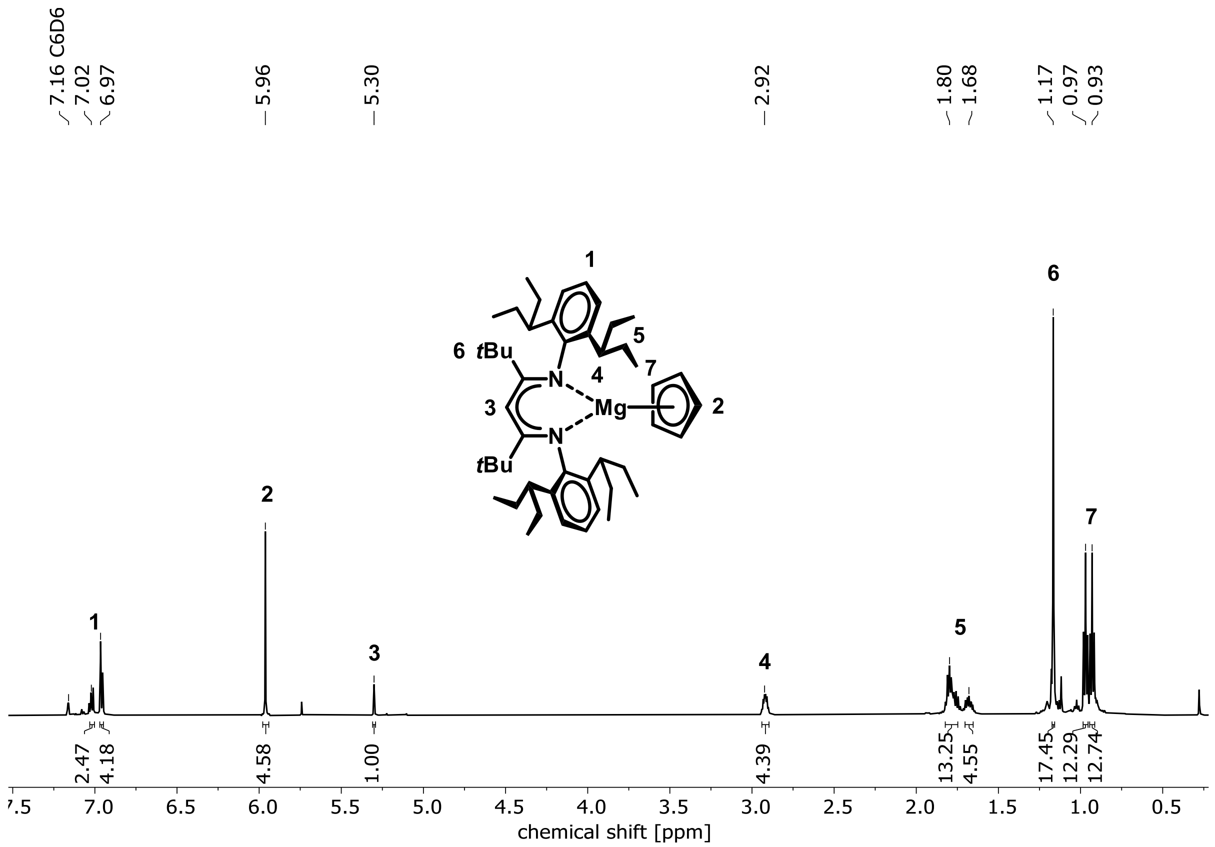

Figure S7. ^{1}H NMR spectrum (600.13 MHz, 298 K, C_6D_6) of (BDI*)MgCp (**2**).

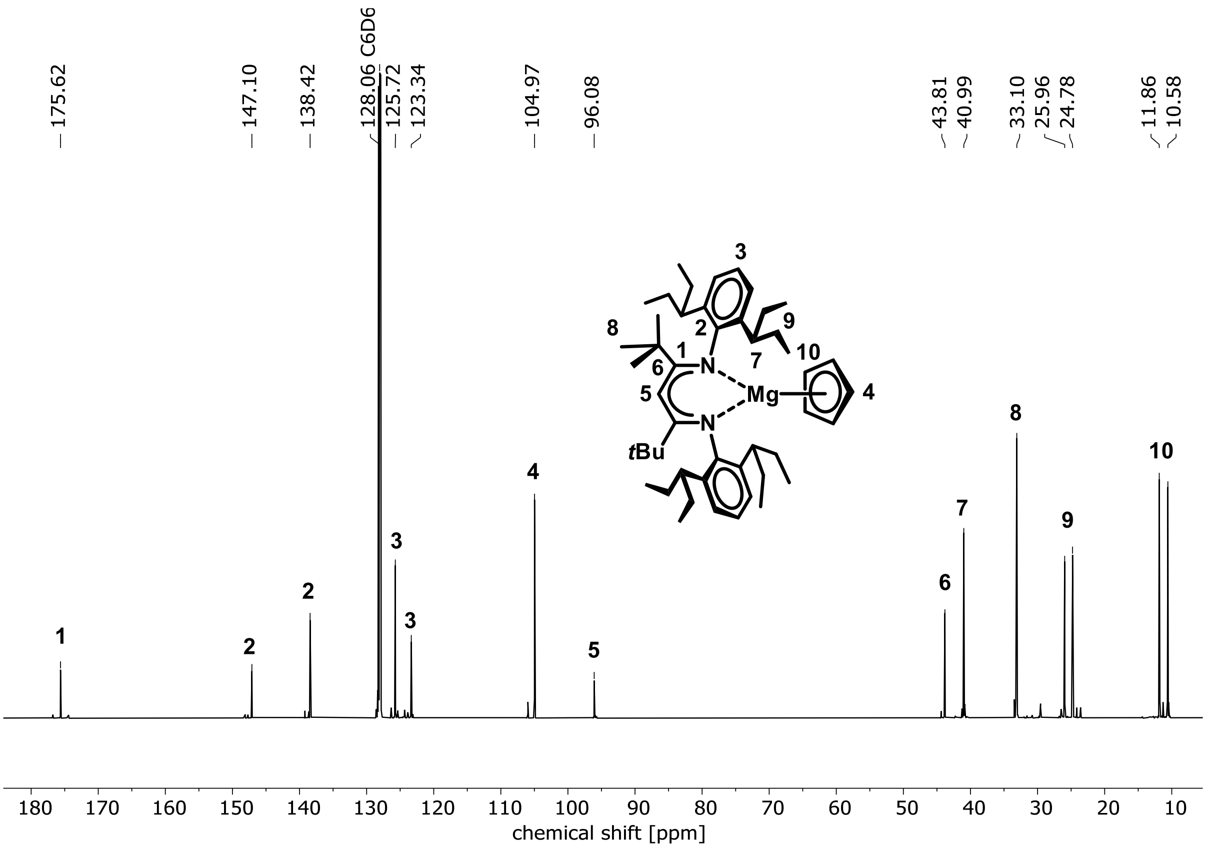

Figure S8. $^{13}C\{^{1}H\}$ NMR spectrum (151 MHz, 298 K, C_6D_6) of (BDI*)MgCp (**2**).

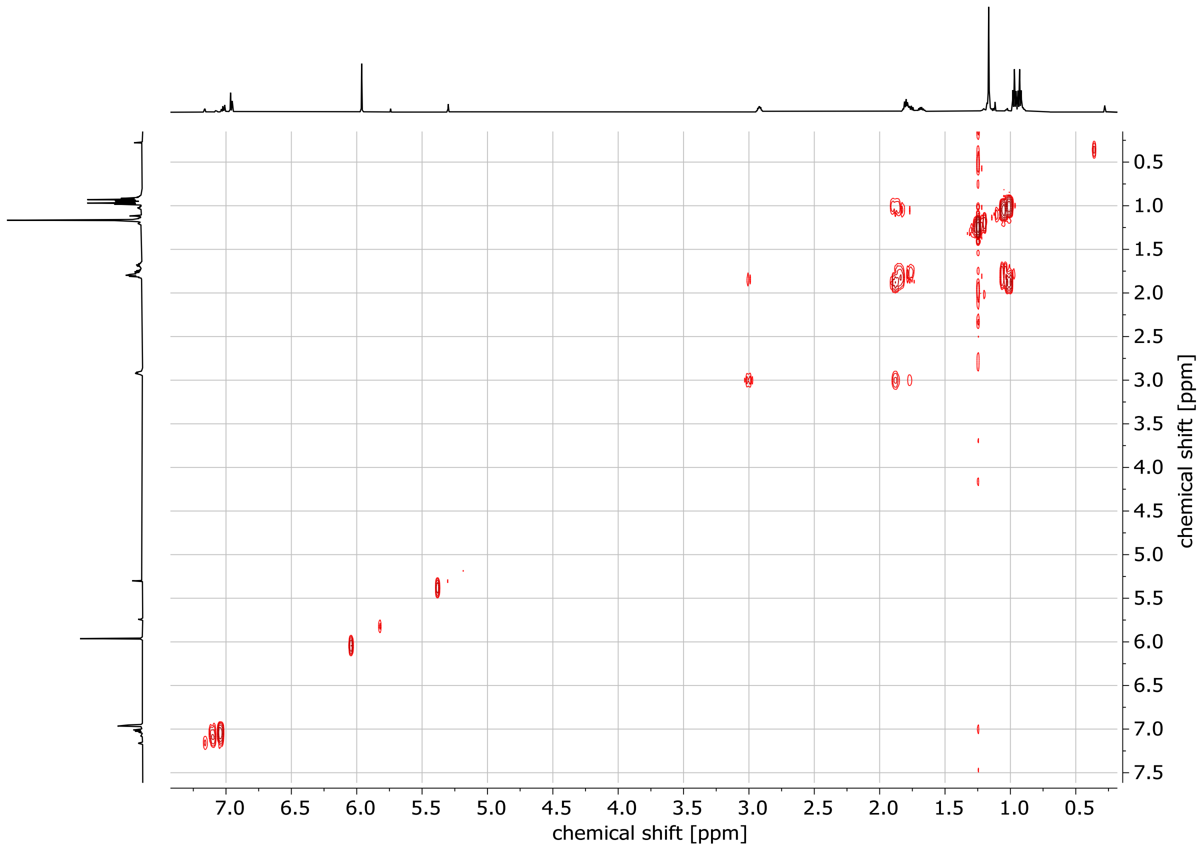

Figure S9. 2D-COSY NMR spectrum (600.13 MHz, 298 K, C_6D_6) of (BDI*)MgCp (**2**).

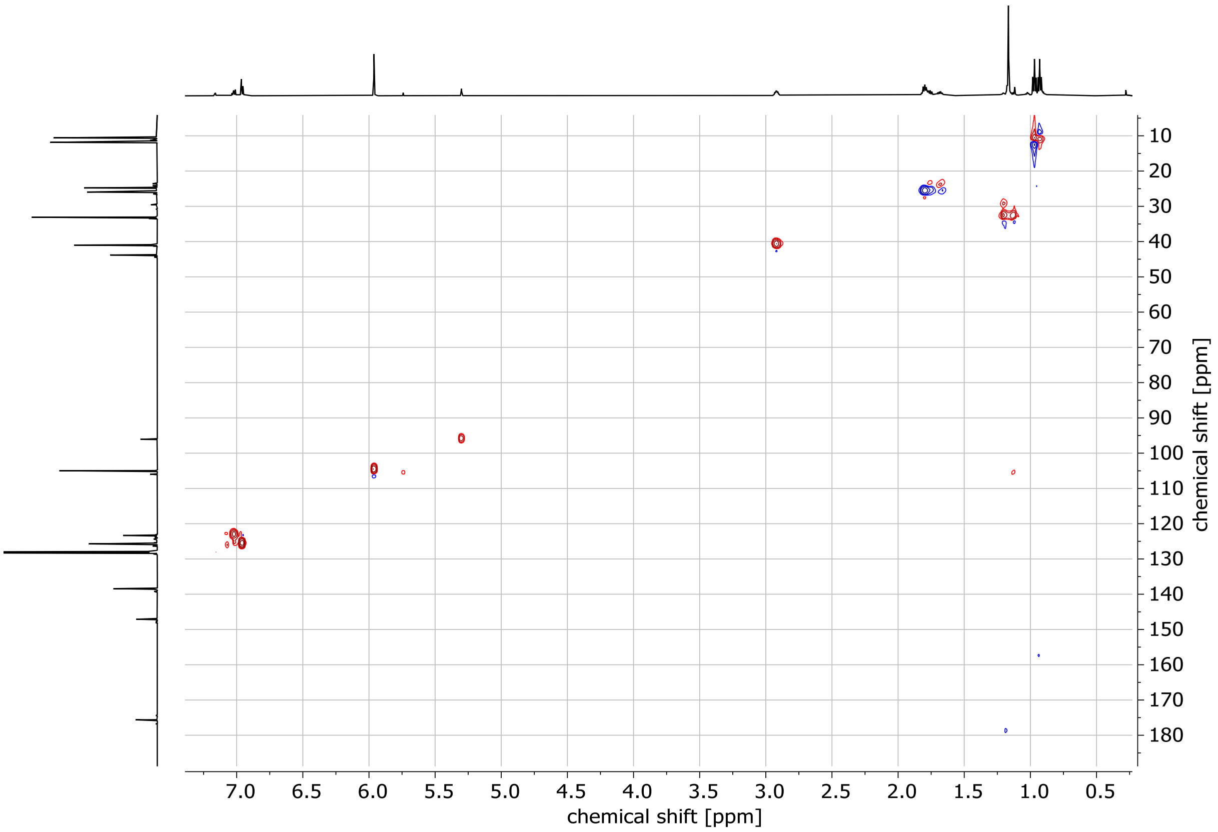

Figure S10. 2D-HSQC NMR spectrum (298 K, C_6D_6) of (BDI*)MgCp (**2**).

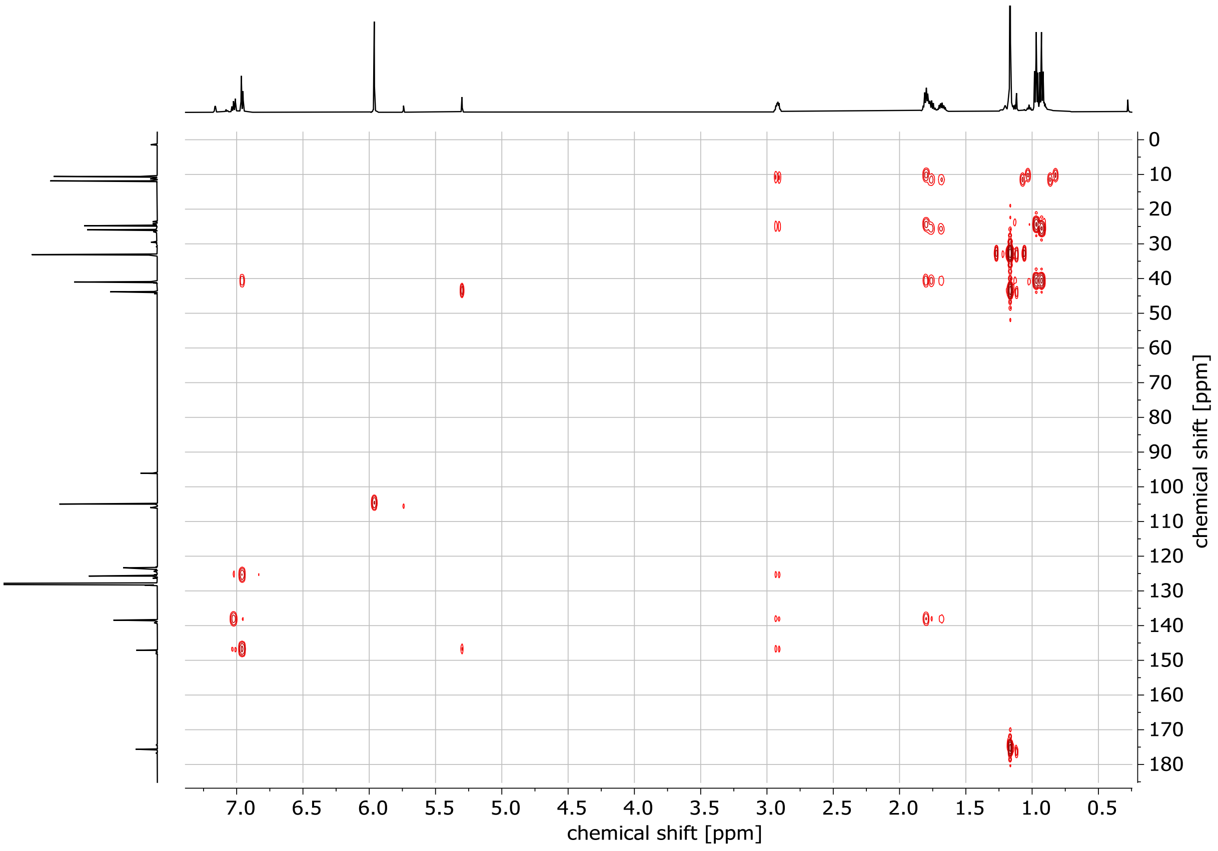

Figure S11. 2D-HMBC NMR spectrum (298 K, C_6D_6) of (BDI*)MgCp (**2**).

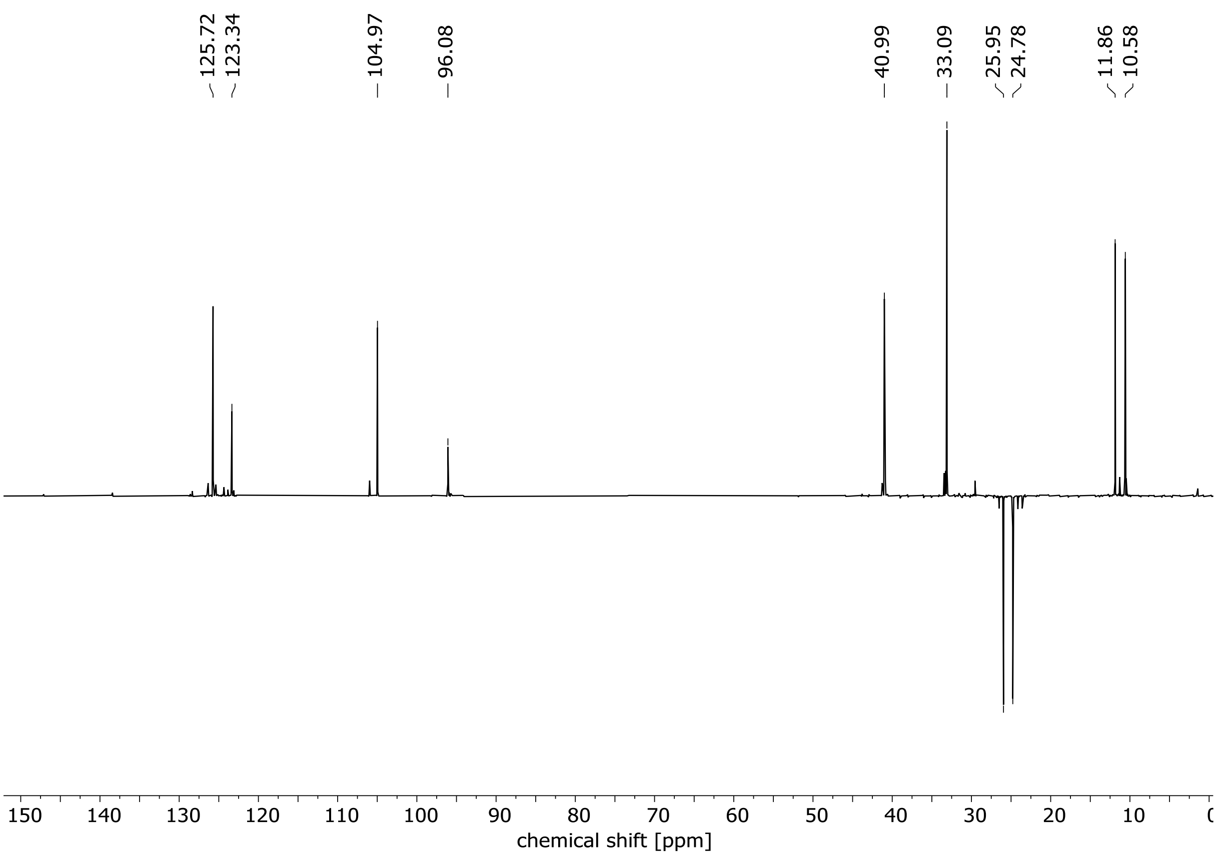

Figure S12. ^{13}C (DEPT 135) NMR spectrum (150.92 MHz, 298 K, C_6D_6) of (BDI*)MgCp (**2**).

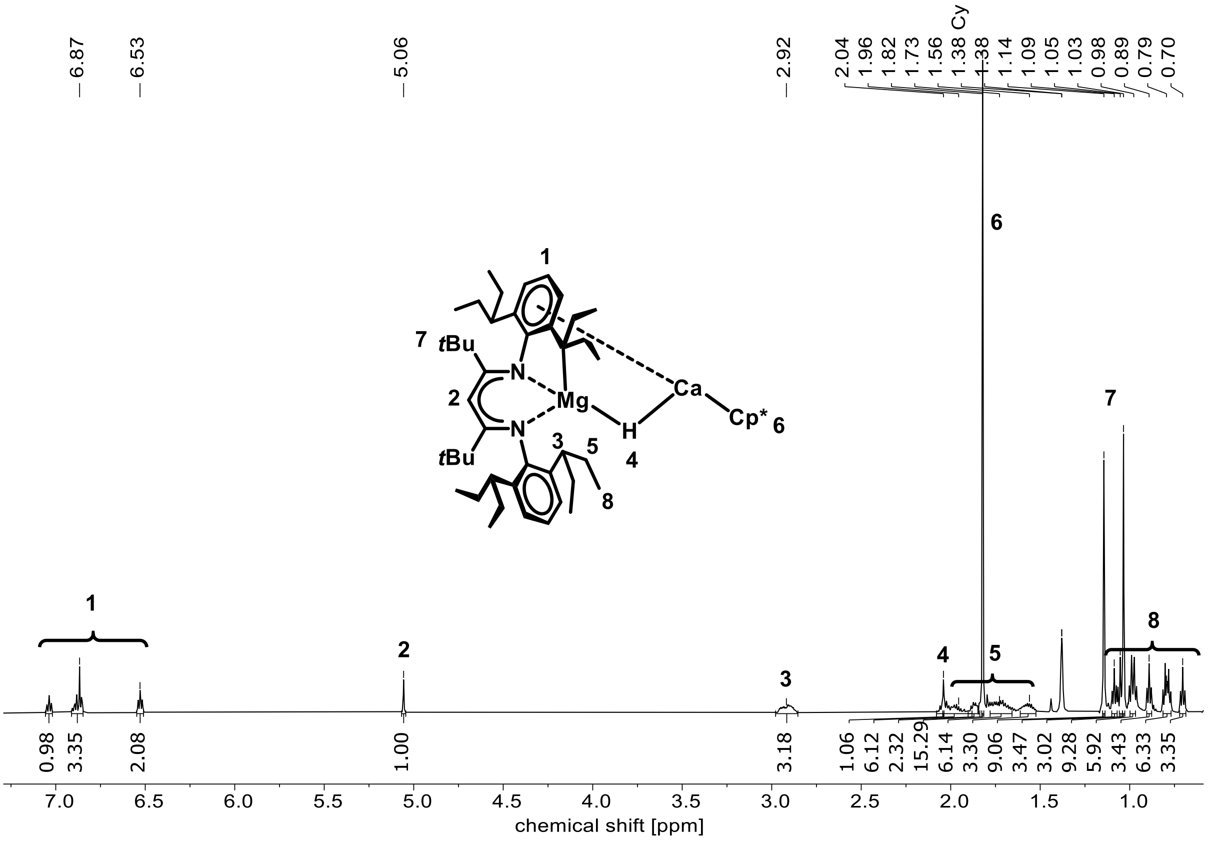

Figure S13. ^1H NMR spectrum (600.13 MHz, 298 K, cyclohecane-d_{12}) of (BDI*-H)Mg(H)CaCp* (**3**).

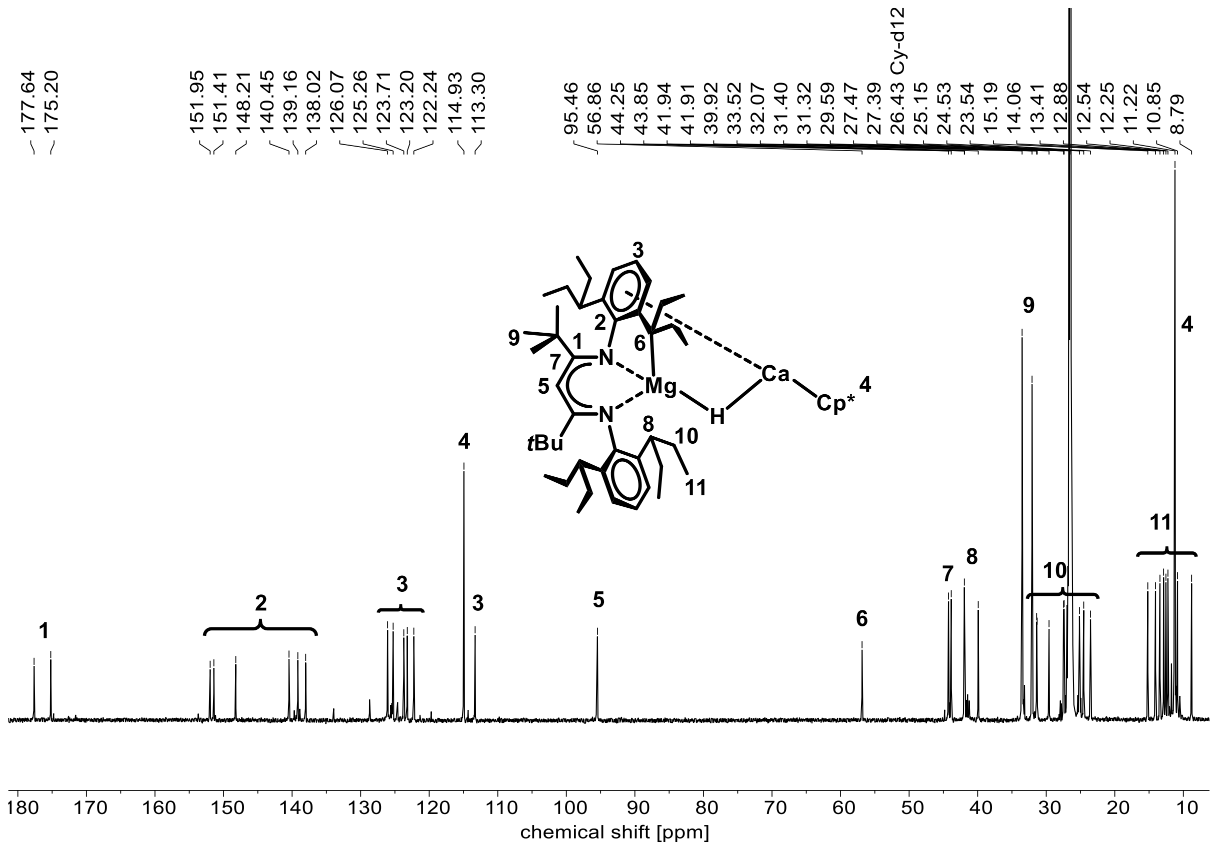

Figure S14. $^{13}C\ \{^{1}H\}$ NMR spectrum (151 MHz, 298 K, cyclohecane-d$_{12}$) of (BDI*-H)Mg(H)CaCp* (**3**).

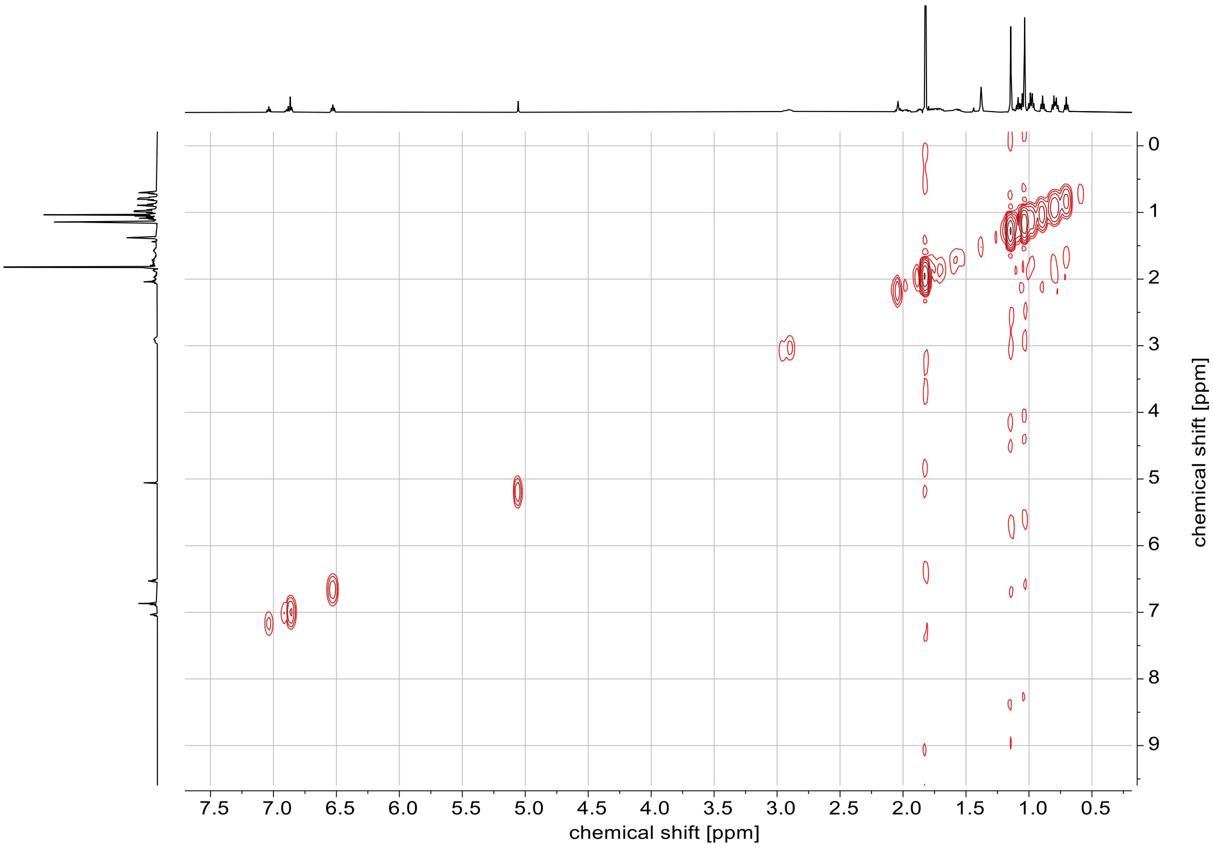

Figure S15. 2D-COSY NMR spectrum (600.13 MHz, 298 K, cyclohecane-d$_{12}$) of (BDI*-H)Mg(H)CaCp* (**3**).

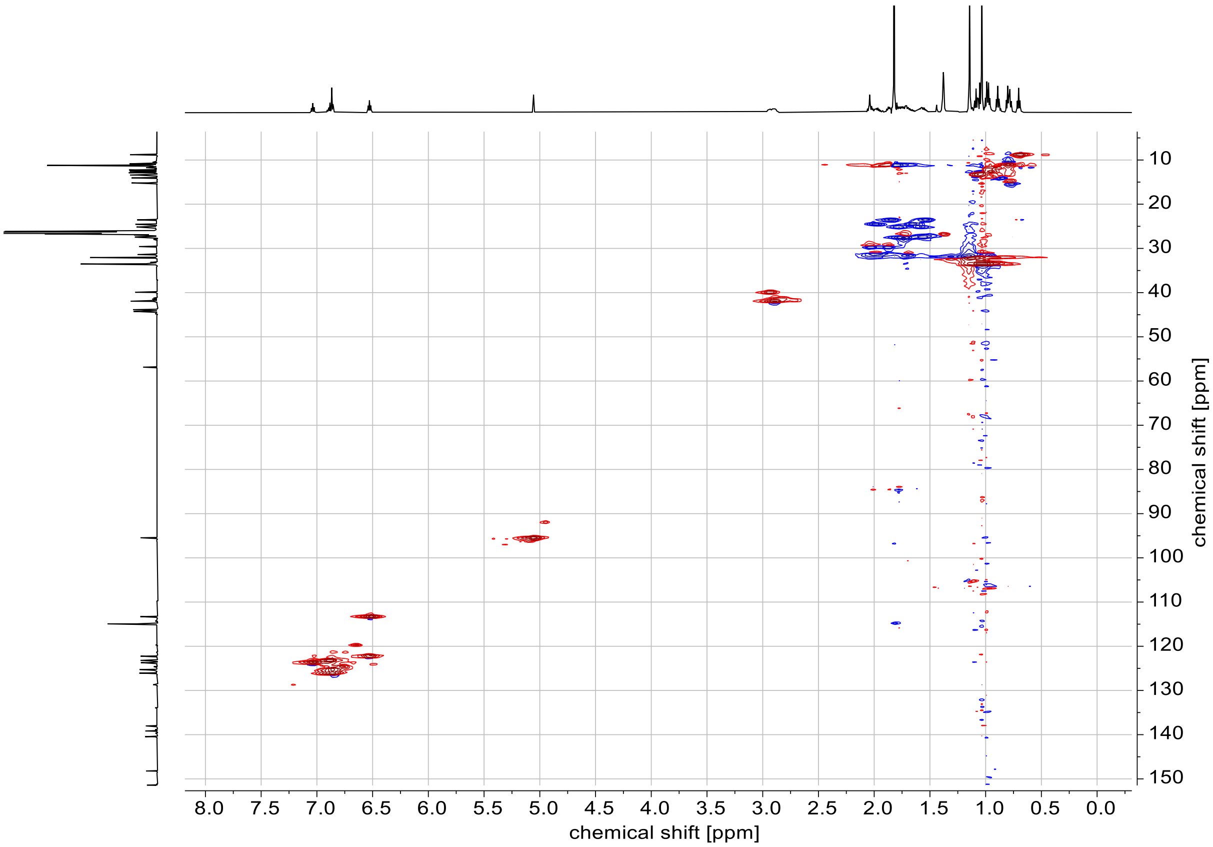

Figure S16. 2D-HSQC NMR spectrum (298 K, cyclohecane-d12) of (BDI*-H)Mg(H)CaCp* (**3**).

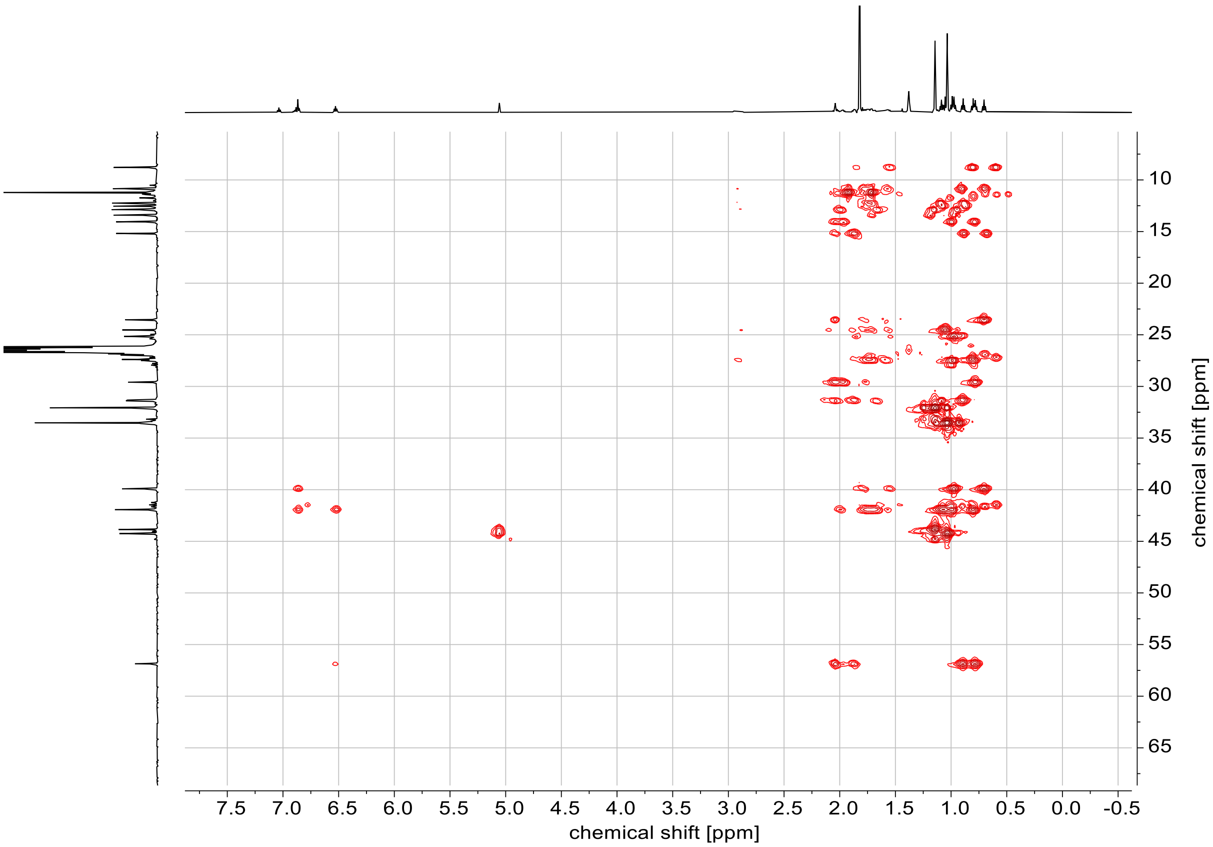

Figure S17. 2D-HMBC NMR spectrum (298 K, cyclohecane-d12) of (BDI*-H)Mg(H)CaCp* (**3**).

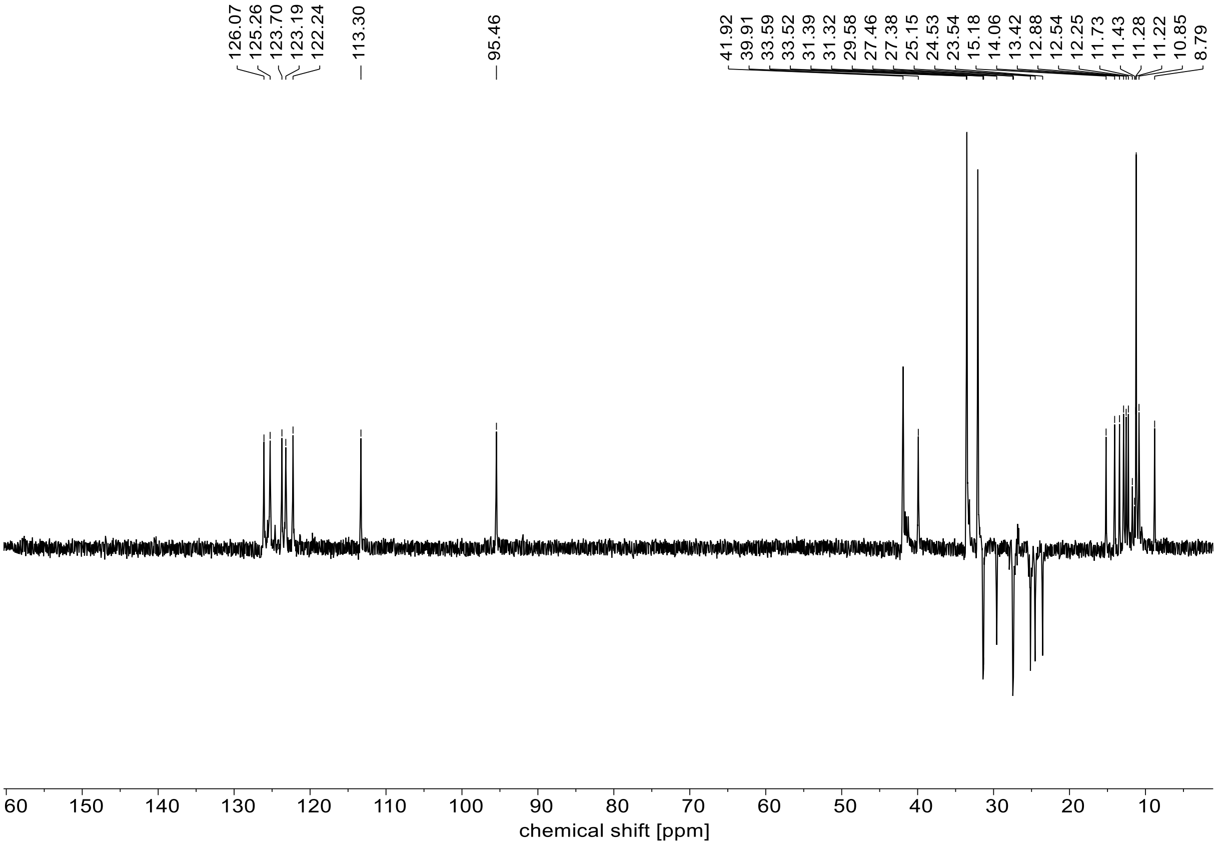

Figure S18. ^{13}C (DEPT 135) NMR spectrum (150.91 MHz, 298 K, cyclohecane-d_{12}) of (BDI*-H)Mg(H)CaCp* (**3**).

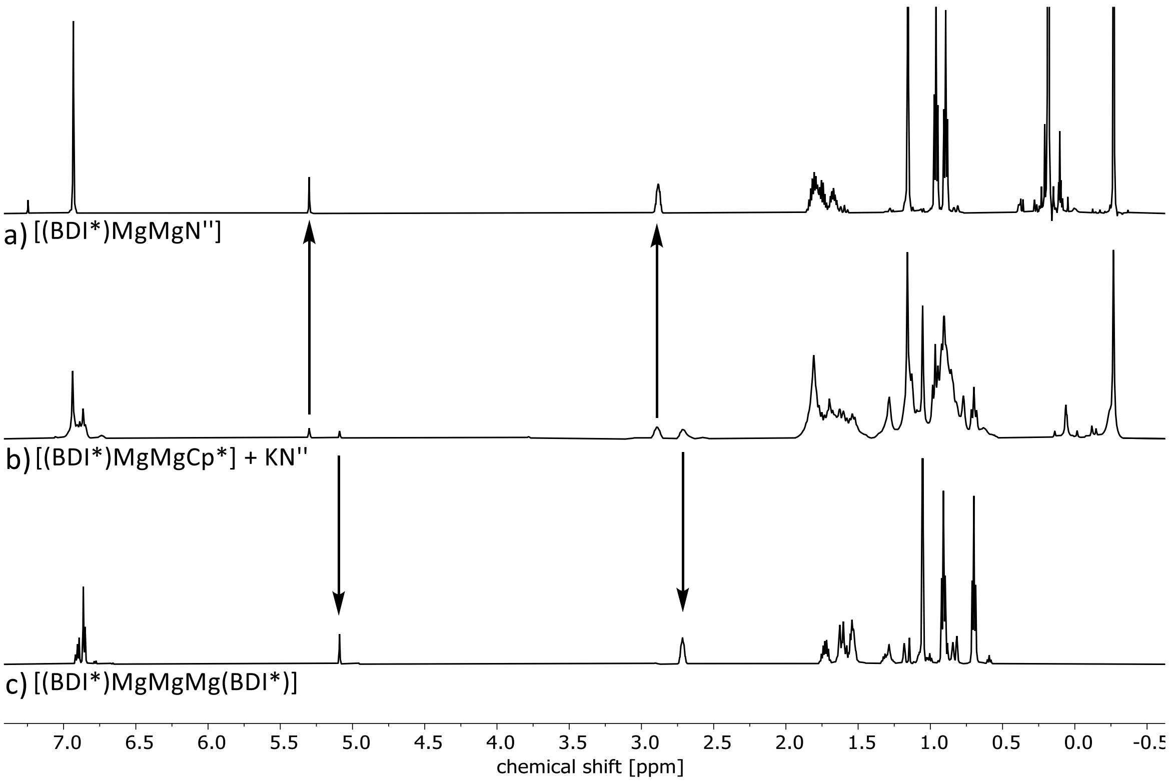

Figure S19. ^{1}H NMR spectrum (600.13 MHz, 298 K, methylcyclohexane-d_{14}) of a) (BDI*)MgMgN($SiMe_3$)$_2$, b) the reaction of **1** with KN($SiMe_3$), c) (BDI*)MgMgMg(BDI*)

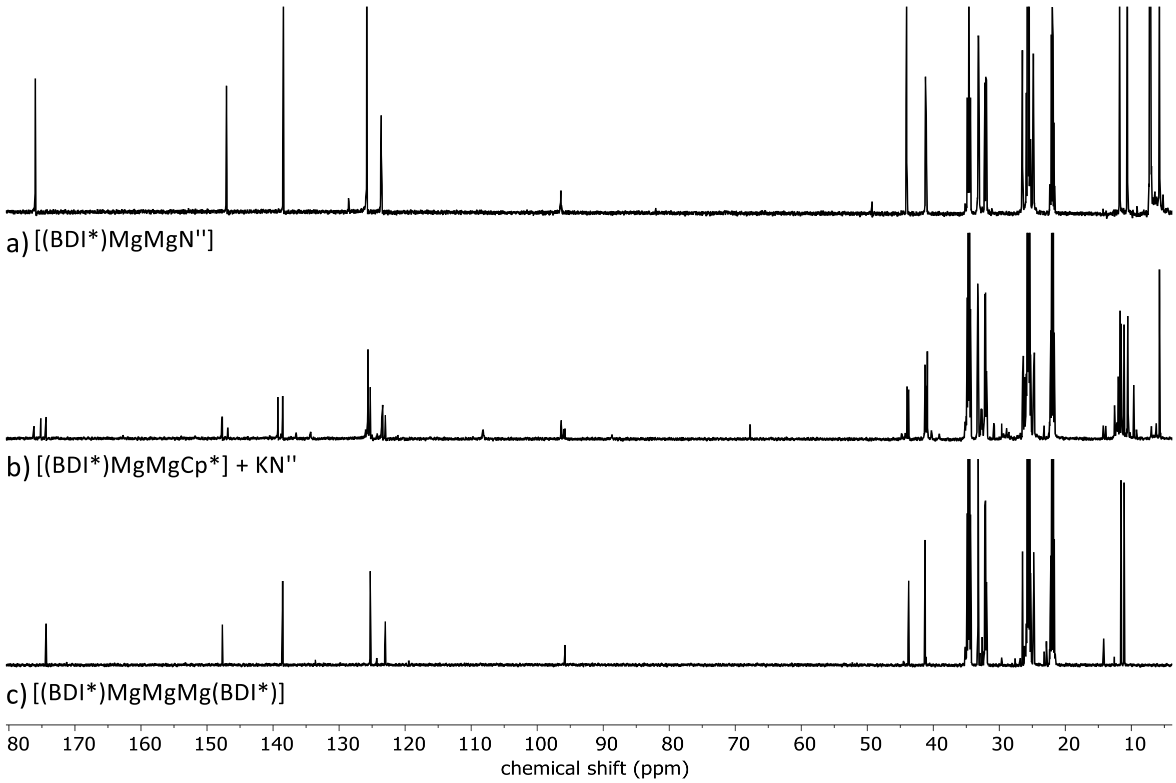

Figure S20. $^{13}C\{^1H\}$ NMR spectrum in the range between 0 ppm and 180 ppm (151 MHz, 298 K, methylcyclohexane-d_{14}) of a) (BDI*)MgMgN($SiMe_3$)$_2$, b) the reaction of **1** with KN($SiMe_3$), c) (BDI*)MgMgMg(BDI*).

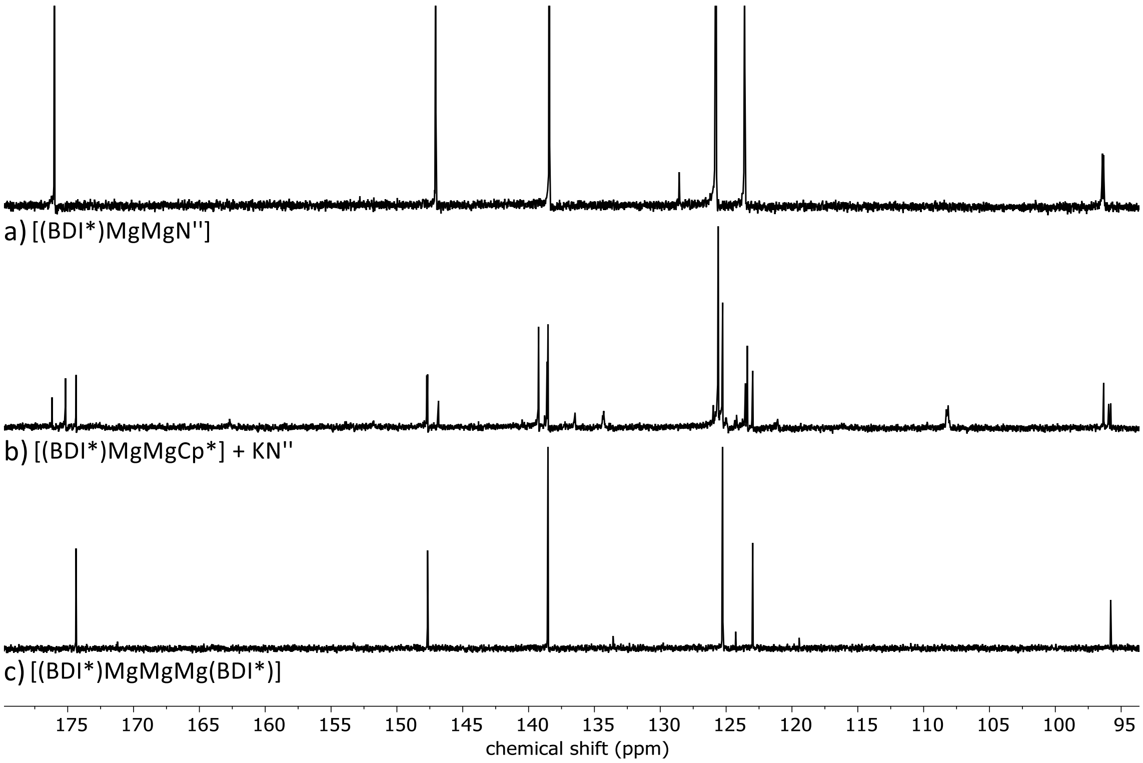

Figure S21. $^{13}C\{^{1}H\}$ NMR spectrum in the range between 180 ppm and 95 ppm (151 MHz, 298 K, methylcyclohexane-d_{14}) of a) (BDI*)MgMgN$(SiMe_3)_2$, b) the reaction of **1** with KN$(SiMe_3)$, c) (BDI*)MgMgMg(BDI*).

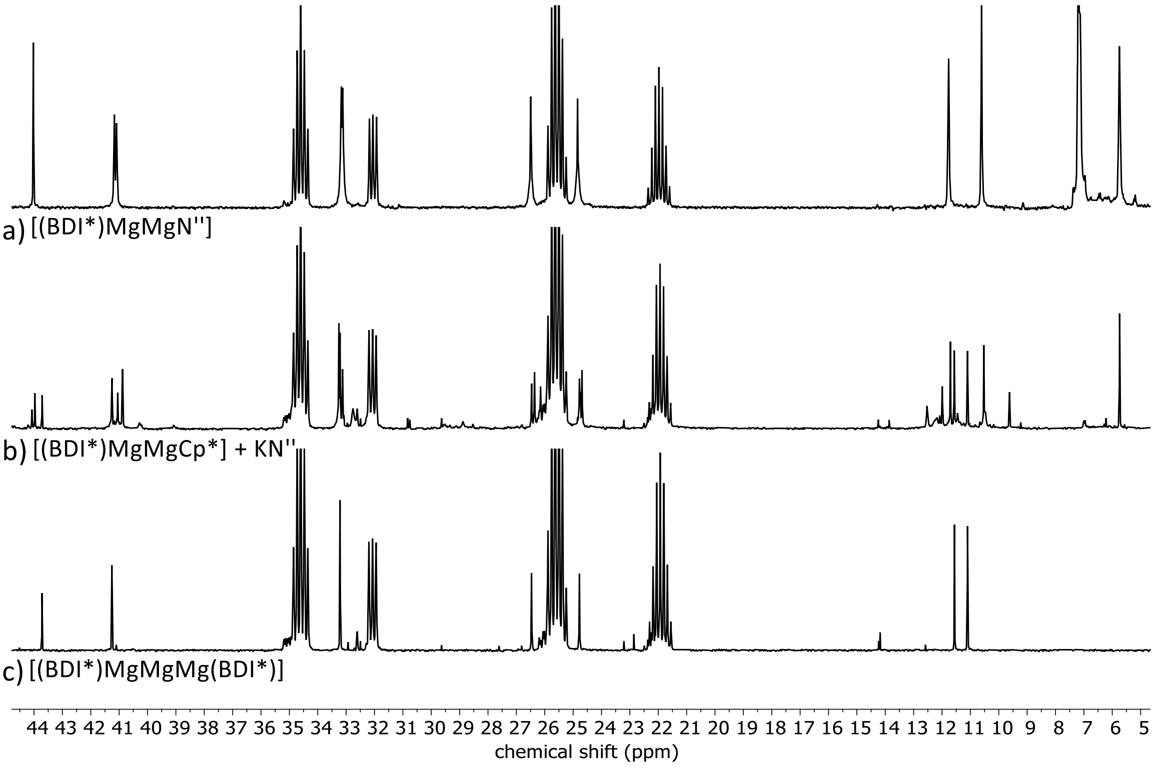

Figure S22. $^{13}C\{^{1}H\}$ NMR spectrum in the range between 45 ppm and 5 ppm (151 MHz, 298 K, methylcyclohexane-d_{14}) of a) (BDI*)MgMgN$(SiMe_3)_2$, b) the reaction of **1** with KN$(SiMe_3)$, c) (BDI*)MgMgMg(BDI*).

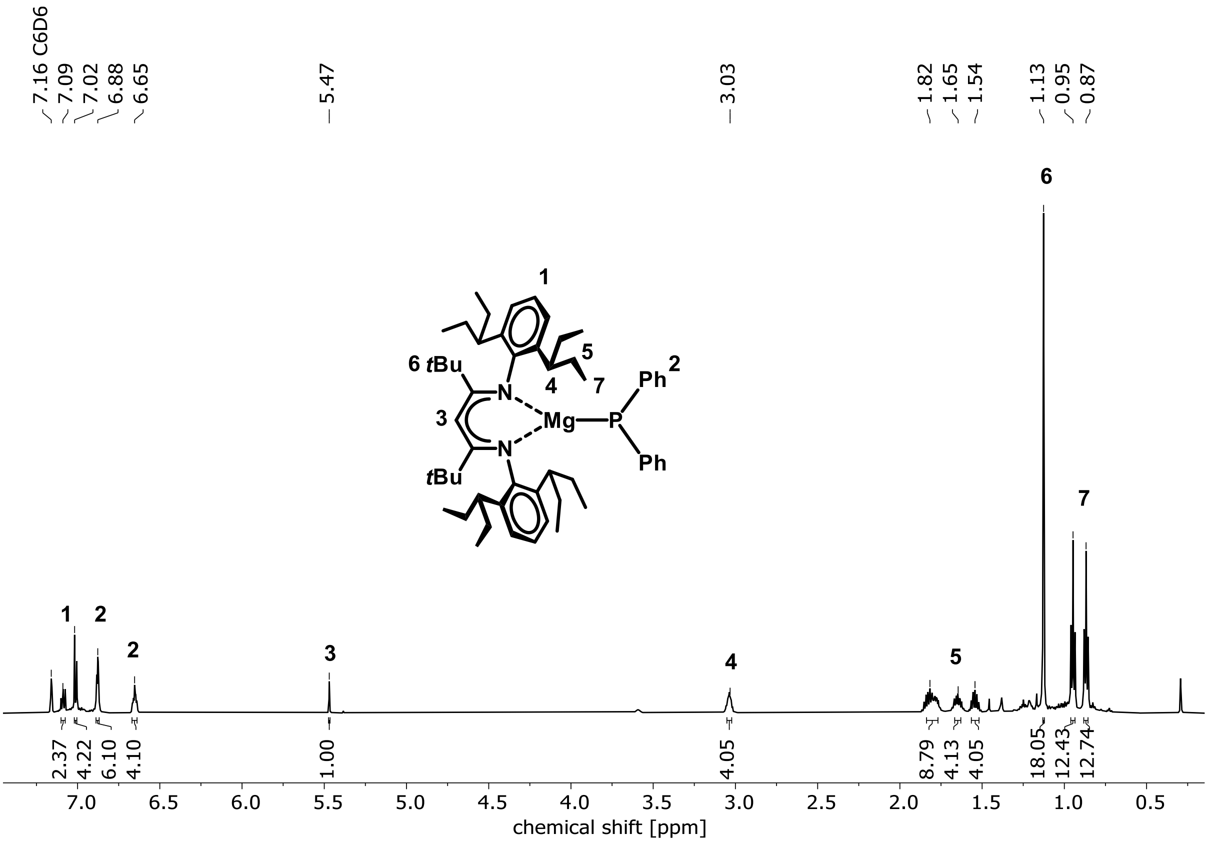

Figure S23. ^{1}H NMR spectrum (600.13 MHz, 298 K, C_6D_6) of (BDI*)MgPPh$_2$ (**4**).

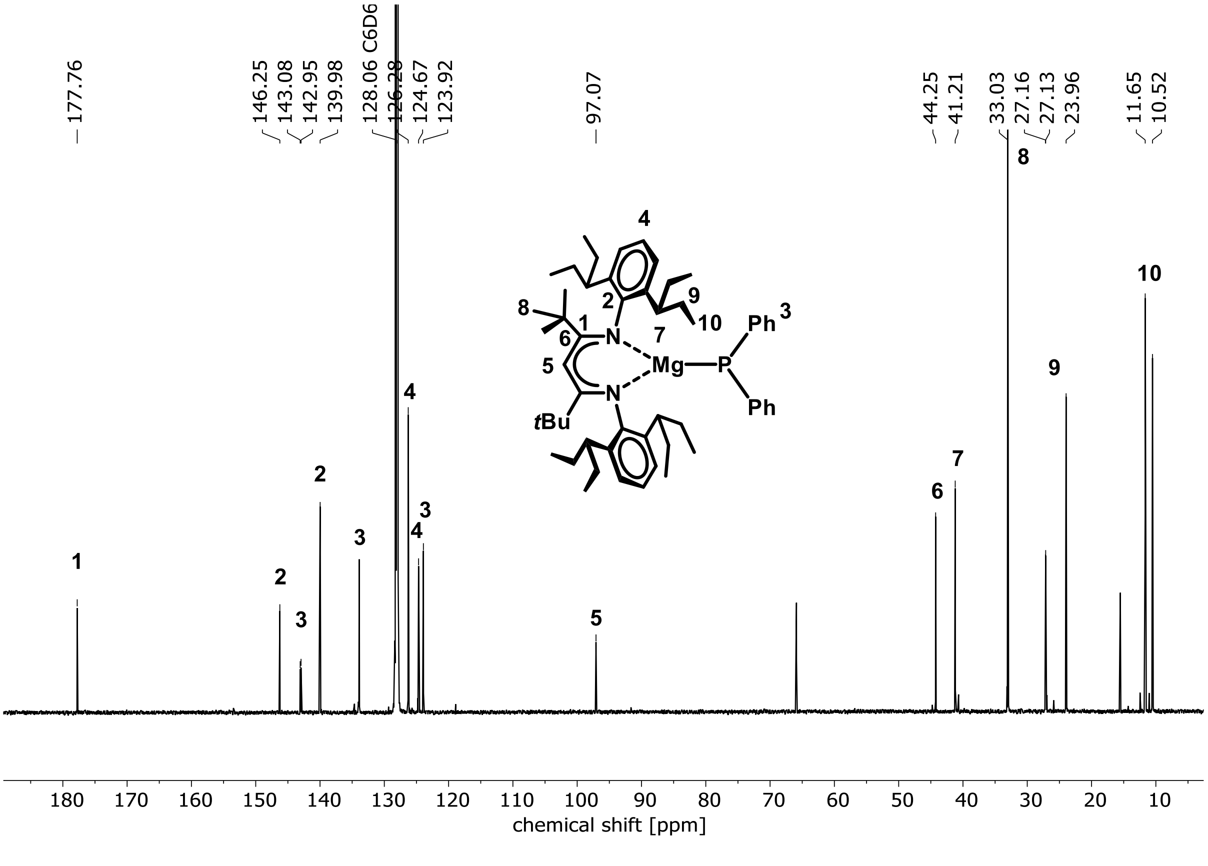

Figure S24. ^{13}C {^{1}H} NMR spectrum (151 MHz, 298 K, C_6D_6) of (BDI*)MgPPh$_2$ (**4**).

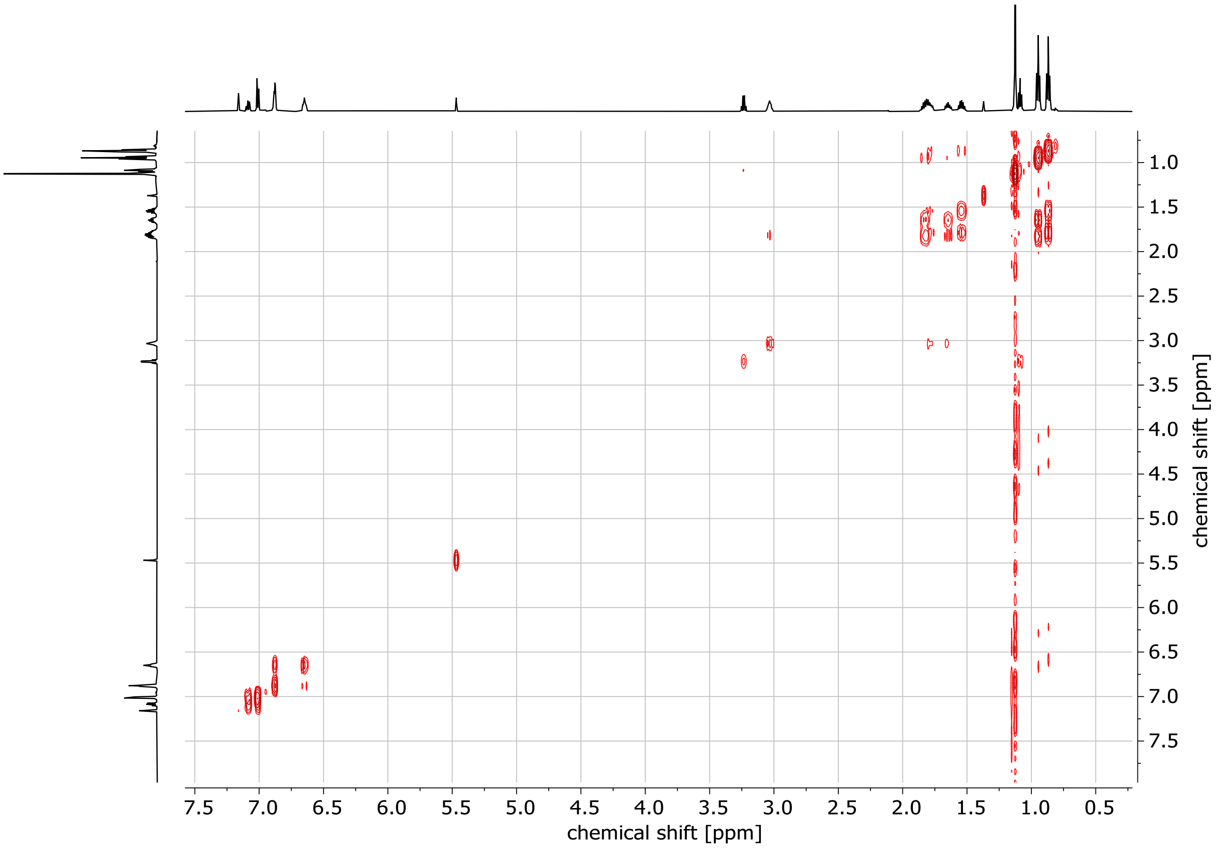

Figure S25. 2D-COSY NMR spectrum (600.13 MHz, 298 K, C_6D_6) of (BDI*)$MgPPh_2$ (**4**).

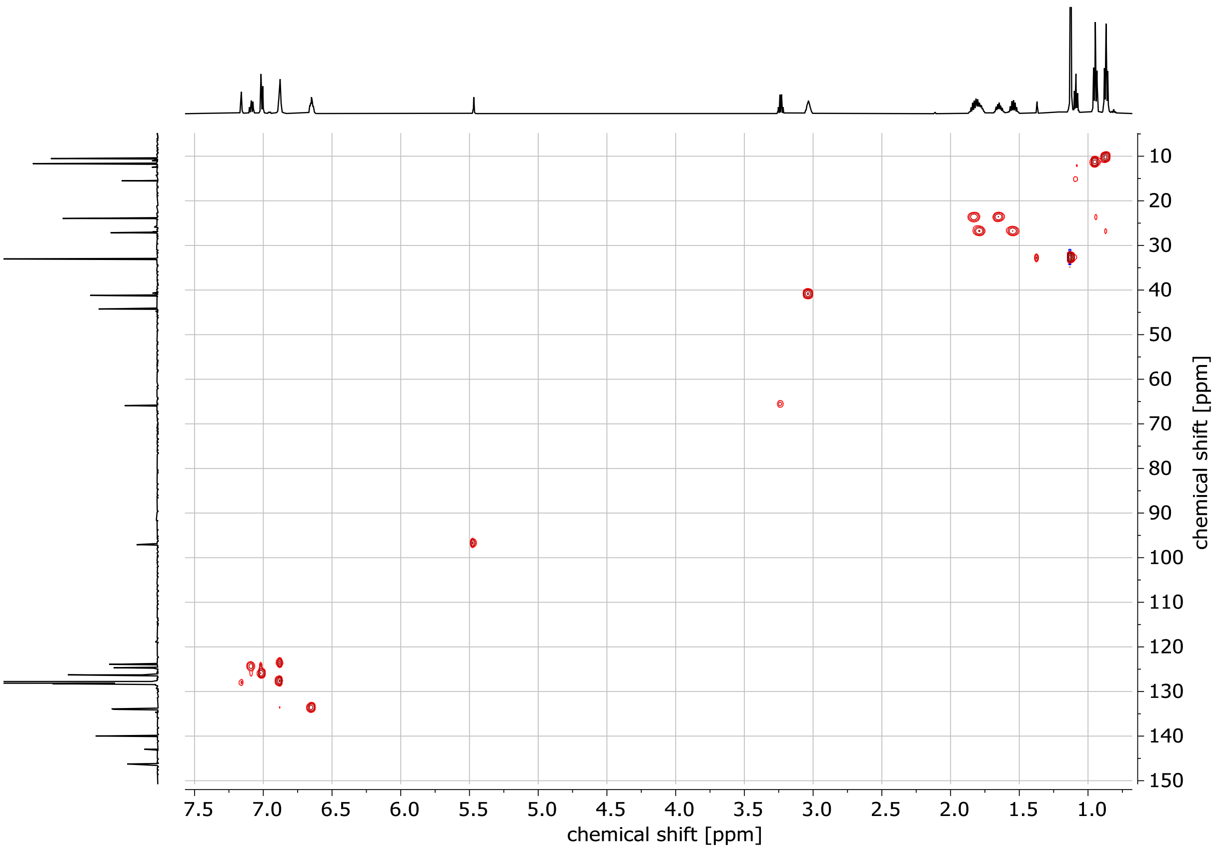

Figure S26. 2D-HSQC NMR spectrum (298 K, C_6D_6) of (BDI*)$MgPPh_2$ (**4**).

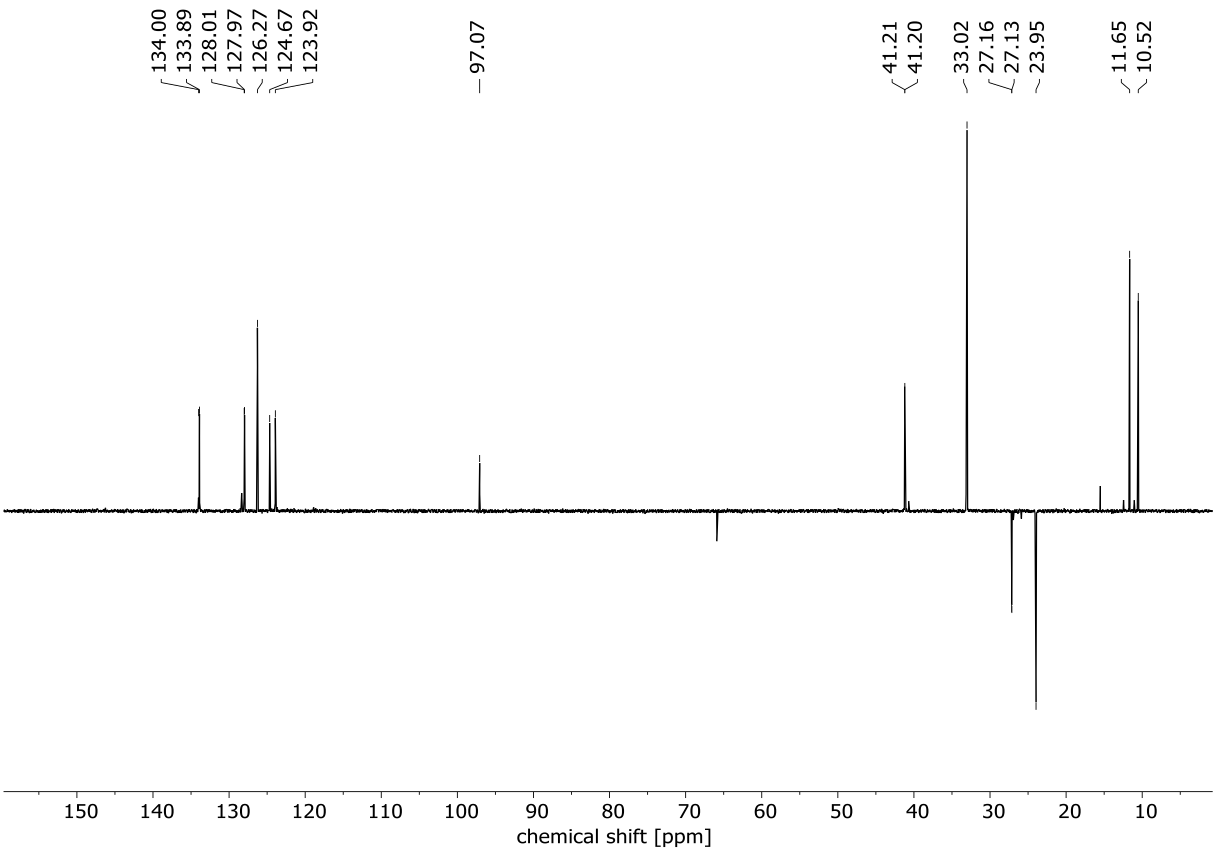

Figure S27. ^{13}C (DEPT 135) NMR spectrum (150.91 MHz, 298 K, C_6D_6) of (BDI*)$MgPPh_2$ (**4**).

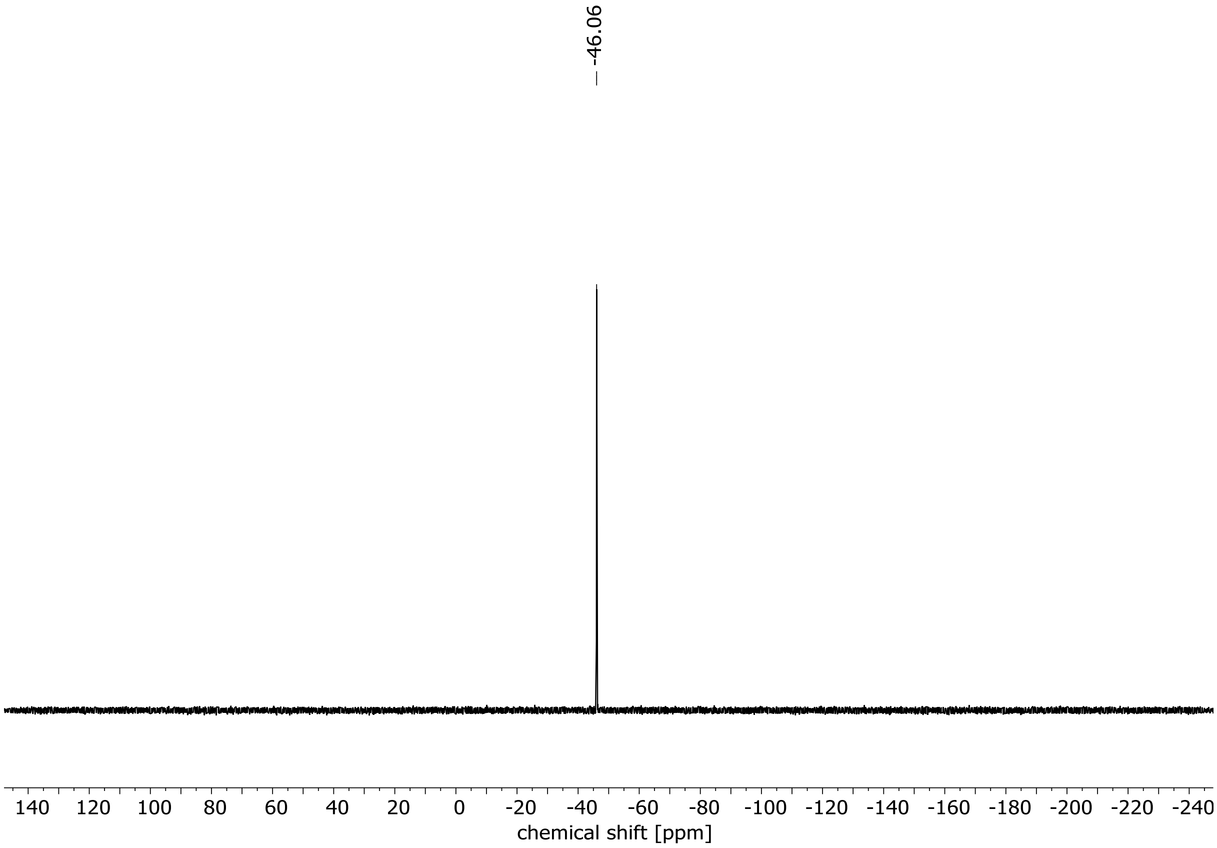

Figure S28. ^{31}P NMR spectrum (242.92 MHz, 298 K, C_6D_6) of (BDI*)$MgPPh_2$ (**4**).

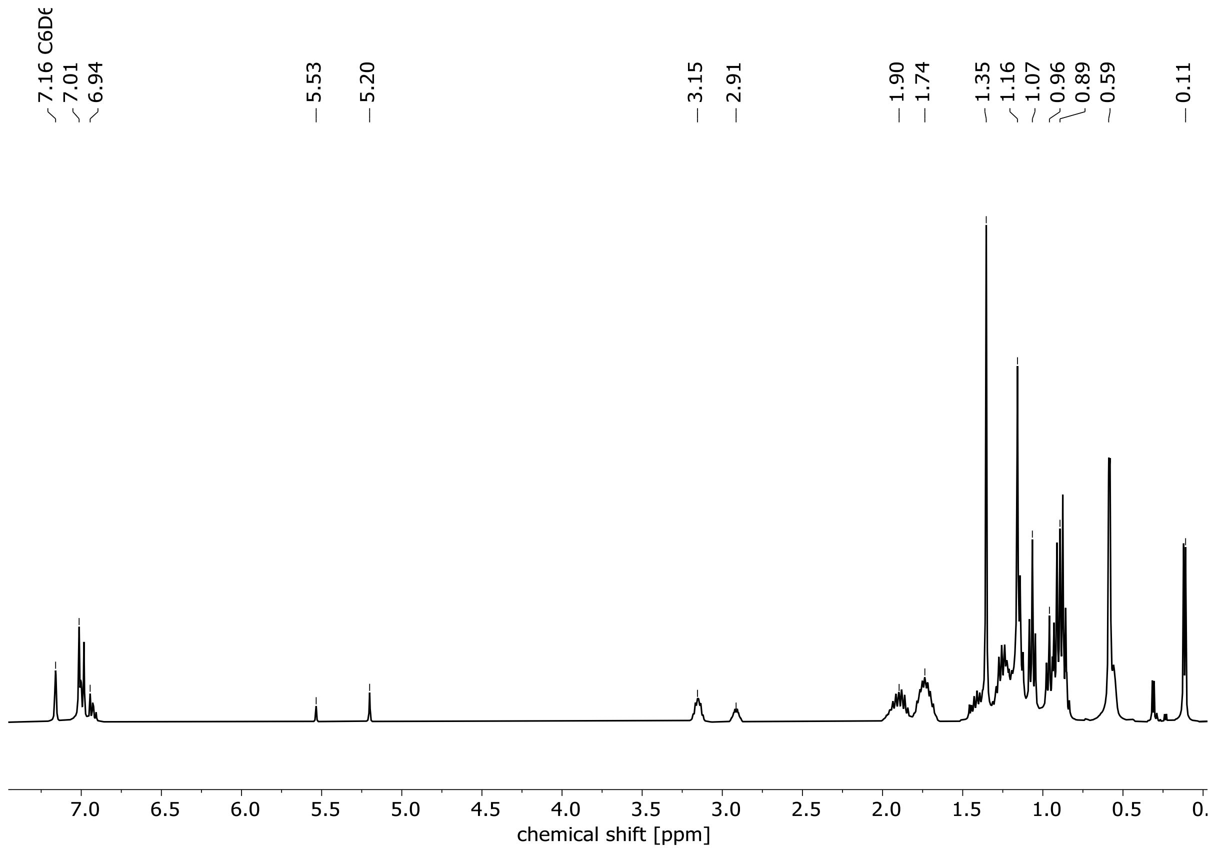

Figure S29. ^{1}H NMR spectrum (600.13 MHz, 298 K, C_6D_6) of the reaction of **1** with $LiP(SiMe_3)_2$.

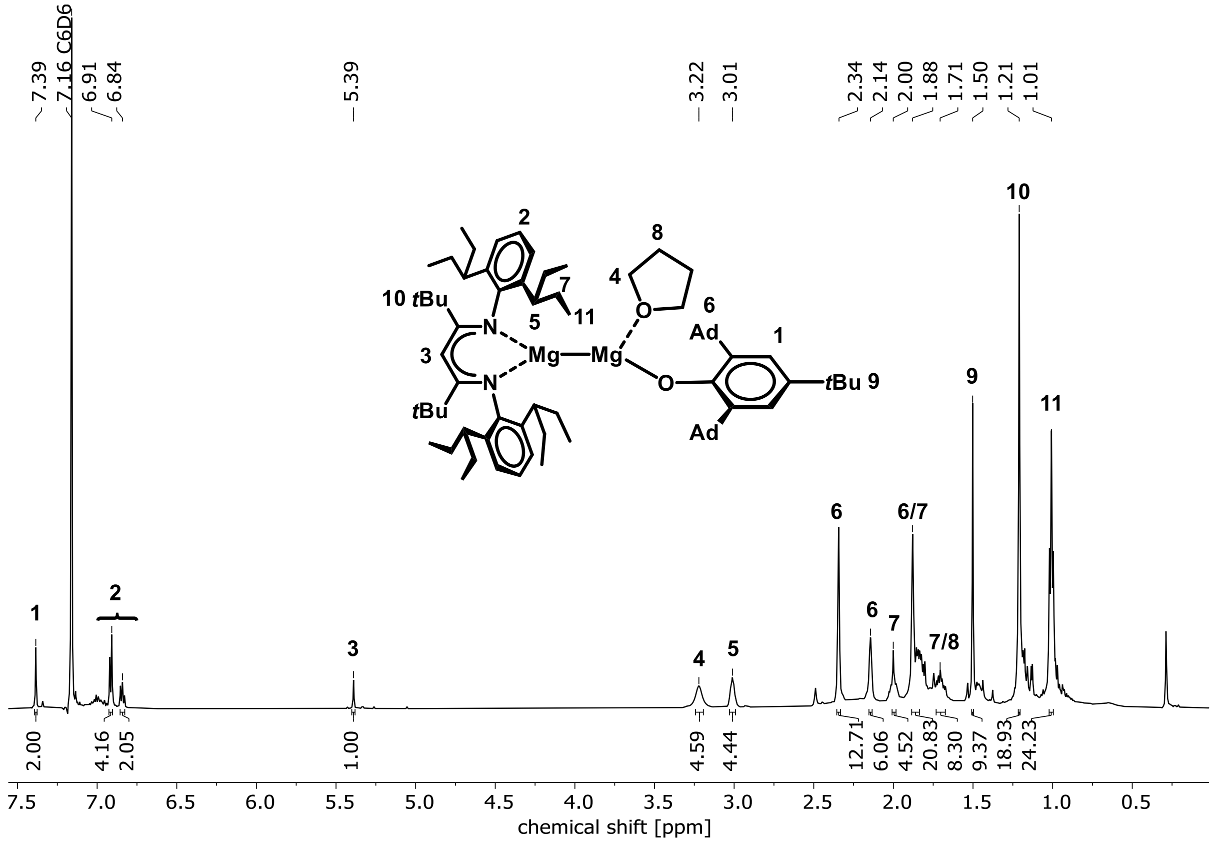

Figure S30. ^{1}H NMR spectrum (600.13 MHz, 298 K, C_6D_6) of the raw product of (BDI*)MgMgOAr·THF (**5**).

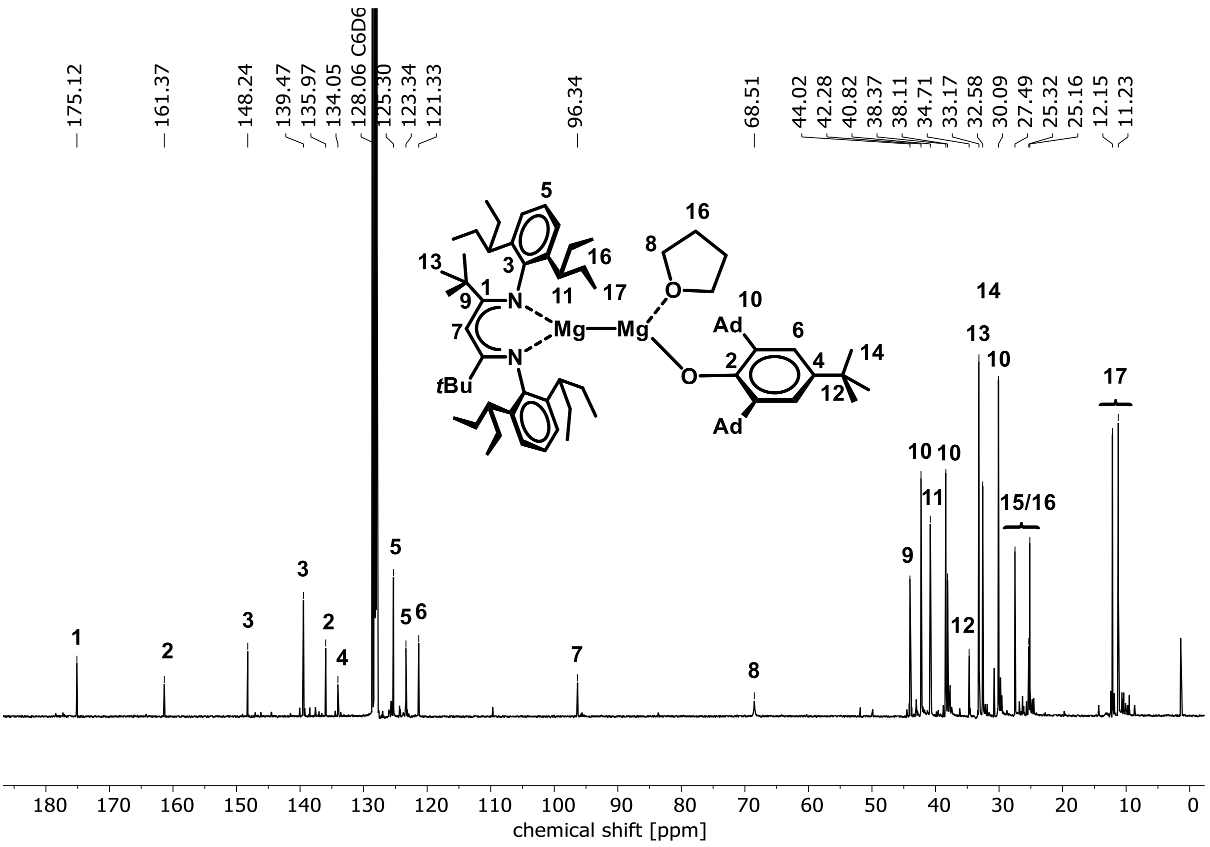

Figure S31. ^{13}C {^{1}H} NMR spectrum (151 MHz, 298 K, C_6D_6) of (BDI*)MgMgOAr·THF (**5**).

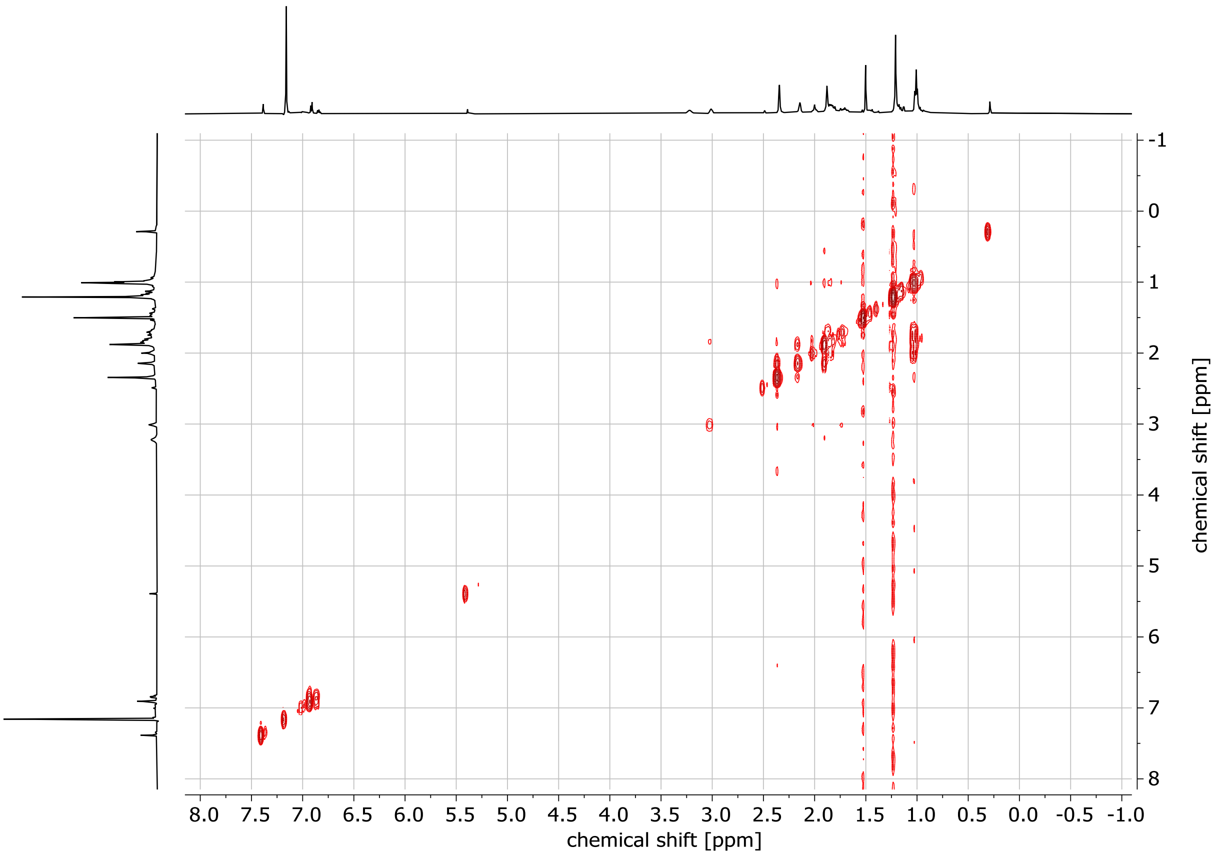

Figure S32. 2D-COSY NMR spectrum (600.13 MHz, 298 K, C_6D_6) of (BDI*)MgMgOAr·THF (**5**).

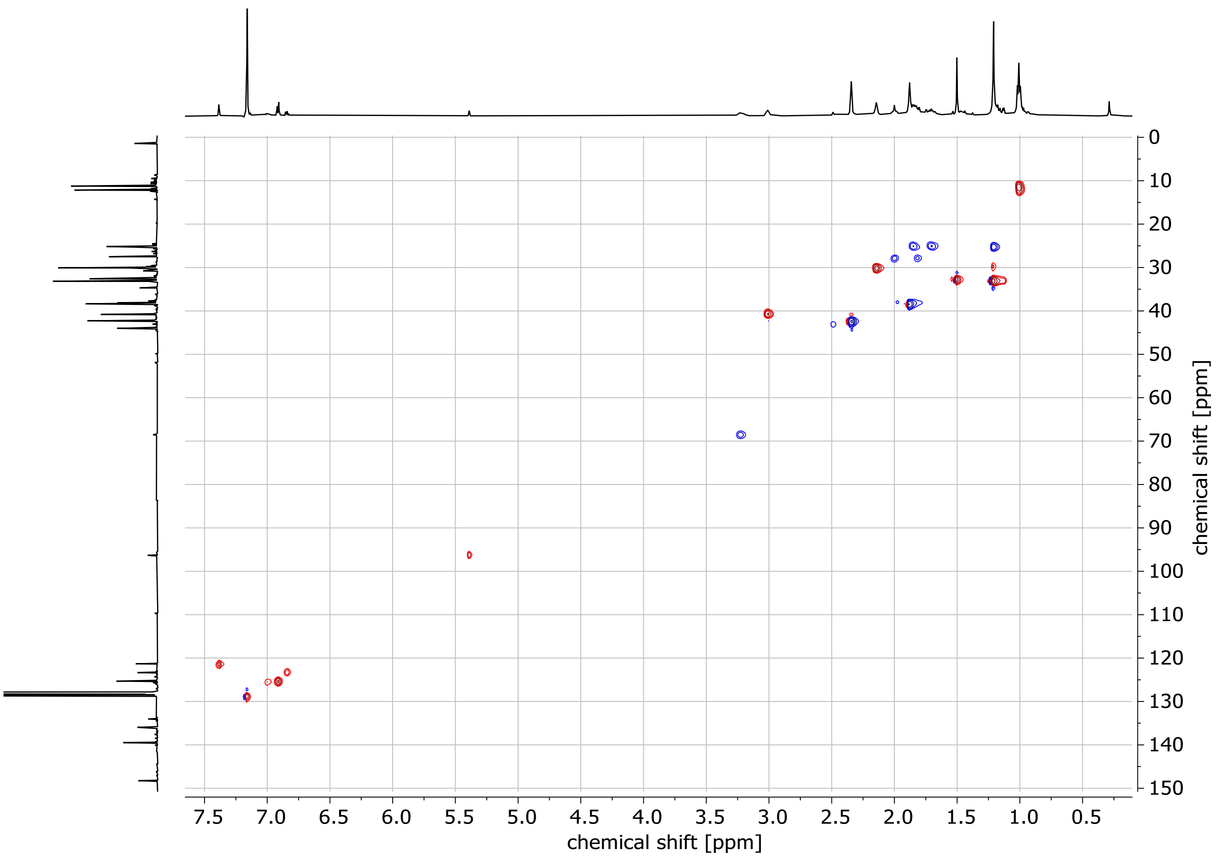

Figure S33. 2D-HSQC NMR spectrum (298 K, C_6D_6) of (BDI*)MgMgOAr·THF (**5**).

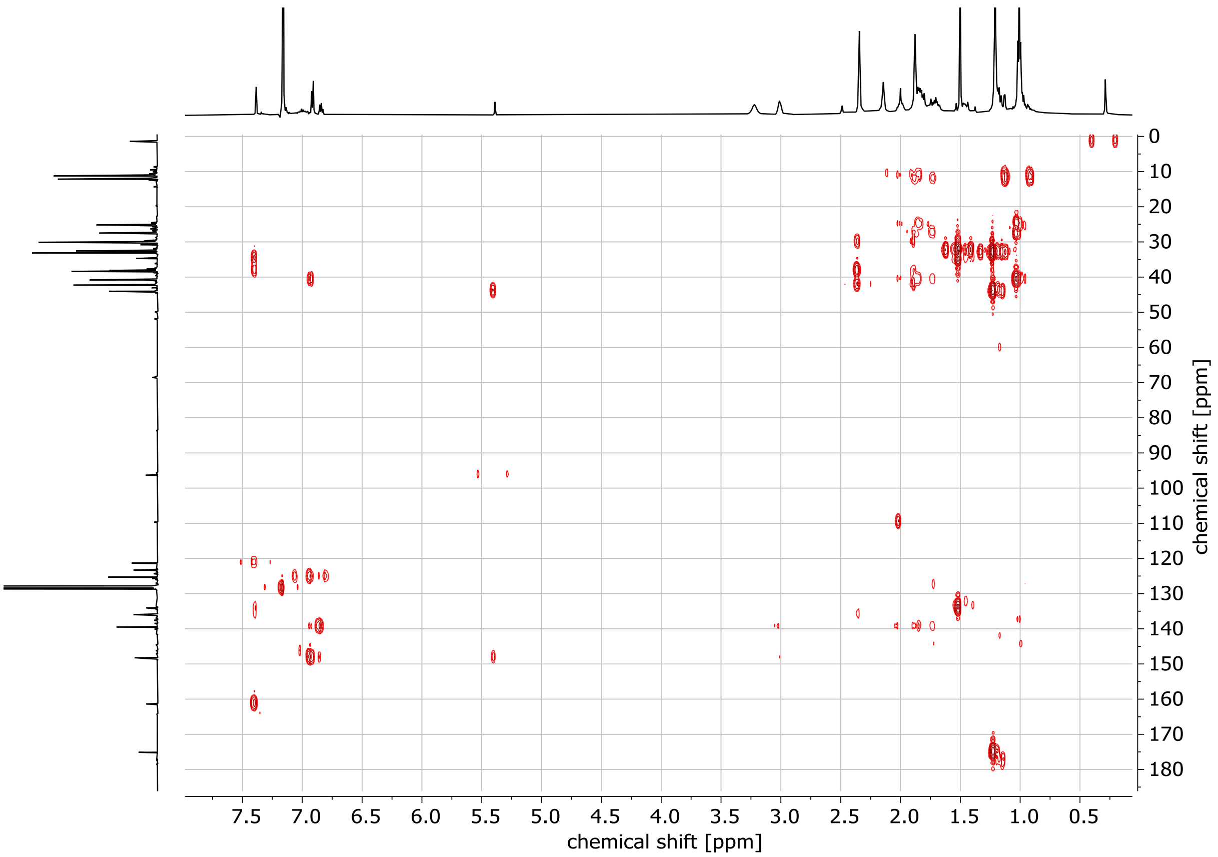

Figure S34. 2D-HMBC NMR spectrum (298 K, C_6D_6) of (BDI*)MgMgOAr·THF (**5**).

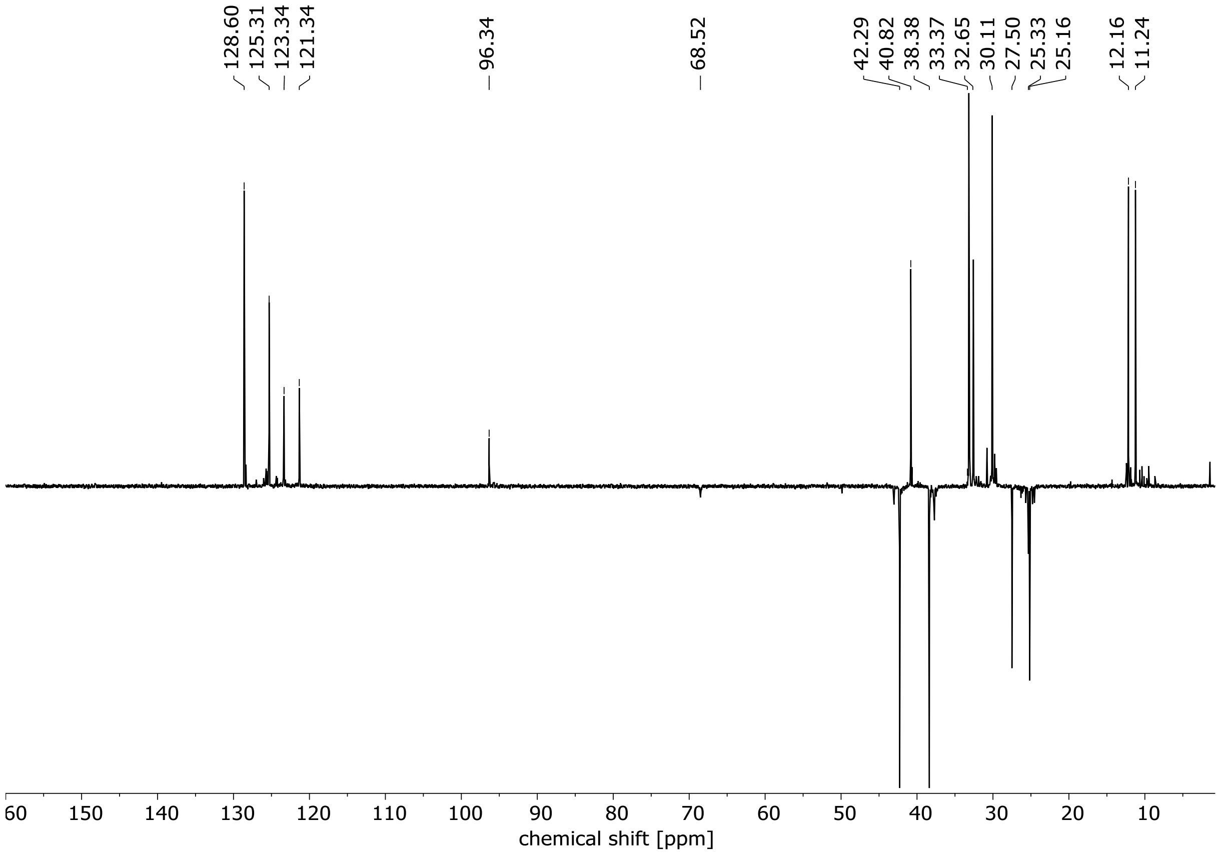

Figure S35. ^{13}C (DEPT 135) NMR spectrum (150.91 MHz, 298 K, C_6D_6) of (BDI*)MgMgOAr·THF (**5**).

4. Crystal structure determinations

Suitable single crystals of compounds **1**-**5**, (BDI*)MgPPh$_2$·THF (**4**-THF), (BDI*)MgP(SiMe$_3$)$_2$ and (BDI*)Li(THF) were embedded in protective perfluoropolyalkylether oil (viscosity 1800 cSt; ABCR GmbH) on a microscope slide and a single specimen was selected and subsequently transferred to the cold nitrogen gas stream of the diffractometer.

The intensity data was collected at 150K (compound (BDI*)MgPPh$_2$·THF (**4**-THF)) or 100 K (all other compounds) using CuK_α radiation (λ = 1.54184 Å) on an Agilent SuperNova dual radiation diffractometer with microfocus X-ray sources and mirror optics. The measured data were processed with the CrysAlisPro software package.[S11] Data were corrected for Lorentz and polarization effects, and an empirical absorption correction using spherical harmonics was applied. In case of (BDI*)MgMgOAr·THF (**5**), an additional analytical absorption correction[S12] was performed, while in all other cases an additional numerical absorption correction based on gaussian integration over a multifaceted crystal model was applied instead. Using Olex2,[S13] the structures were solved by dual-space methods (SHELXT)[S14] and refined by full-matrix least-squares procedures on F^2 using SHELXL.[S15] All non-hydrogen atoms were refined with anisotropic displacement parameters. Most hydrogen atoms were placed in geometrically calculated positions and refined by using a riding model where each H-atom was assigned a fixed isotropic displacement parameter with a value equal to 1.2U_{eq} (CH or CH$_2$) or 1.5U_{eq} (CH$_3$) of its parent C-atom.

The crystals of compound (BDI*)MgCp (**2**) contained co-crystallized severely disordered toluene. The contribution of this solvent moiety to the structure factors was secured by back-Fourier transformation using the solvent mask routine[S16] of the program Olex2.[S13] The solvent accessible voids per unit cell treated this way had a size of 633.6 Å^3 (13.7% of the unit cell) and contained 98.2 electrons/unit cell. This is consistent with 0.5 toluene per asymmetric unit or 2 toluene per unit cell (100 electrons/unit cell).

The position of the hydride ligand of (BDI*-H)Mg(H)CaCp* (**3**) was taken from a difference electron density map and was refined together with an isotropic displacement parameter. A methyl group of the Cp* ligand in this compound was slightly disordered. The relative occupancies of the two alternative orientations of this group were refined to 0.56(3) and 0.44(3), respectively.

The co-crystallized diethyl ether in case of compound (BDI*)MgPPh2 (**4**) is disordered as well. A suitable disorder model could be built with the help of similarity restraints (SADI, SIMU) and rigid bond restraints (RIGU).[S17] Since this diethyl ether is disordered about an inversion center, its occupancy was constrained to 0.5.

The co-crystallized solvent in the crystals of (BDI*)$MgPPh_2$·THF (**4**-THF) was also severely disordered. In this case, it was not clear, which solvent is actually present in the crystal (a mixture of benzene, pentane, diethyl ether and small amounts of THF was used for crystallization). Therefore, the contribution of the solvent to the structure factors was secured by back-Fourier transformation using the solvent mask routine[S16] of the program Olex2.[S13] The solvent accessible voids per unit cell treated this way had a size of 925.8 $Å^3$ (15.8% of the unit cell) and contained 175.8 electrons/unit cell.

The crystal of compound (BDI*)MgMgOAr·THF (**5**) was a non-merohedral twin. The fractional contributions of the two twin domains were refined to 0.5048(9) and 0.4952(9) later on. The asymmetric unit contained two molecules of the magnesium complex and two co-crystallized cyclohexanes. Disorder affected both solvent molecules, the two dipep moieties of one of the magnesium complexes as well as the tert-butyl group of the phenolate ligand of the other magnesium complex. Refinement of the disorder succeeded with the help of similarity restraints (SADI, SIMU) and rigid bond restraints (RIGU).[S17] An additional ISOR restraint was applied to C167. Site occupancy factors of 0.874(6)/0.126(6) (cyclohexane 1), 0.76(2)/0.24(2) (cyclohexane 2), 0.628(3)/0.372(3) (dipep 1), 0.699(3)/0.301(3) (dipep 2) and 0.668(7)/0.332(7) (*t*Bu) were determined for the two alternative orientations of the disordered groups, respectively. However, it was necessary to introduce a third orientation for two of the 3-pentyl moieties within the disordered dipep substituents, in order to describe the observed disorder properly. The site occupancy factors for these parts of the molecule were 0.452(3)/0.301(3)/0.246(3) (3-pentyl 1) and 0.389(3)/0.372(3)/0.240(3) (3-pentyl 2), respectively.

In case of the lithium complex [(BDI*)Li(THF)], racemic twinning was observed. The fractional contributions of the two twin domains were refined to 0.7(4) and 0.3(4) later on.

Deposition Number 2477181 (for (BDI*)MgMgCp*, **1**), 2477182 (for (BDI*-H)Mg(H)CaCp*, **3**), 2477183 (for (BDI*)MgCp, **2**), 2477184 (for [(BDI*)$MgPPh_2$·Et_2O, **4**-Et_2O), 2477185 (for (BDI*)$MgPPh_2$·THF, **4**-THF), 2477186 (for (BDI*)MgP($SiMe_3$)$_2$), 2477187 (for (BDI*)MgMgOAr·THF, **5**) and 2477188 (for (BDI*)Li·THF) contain the supplementary crystallographic data for this paper. These data are provided free of charge by the joint Cambridge Crystallographic Data Centre and Fachinformationszentrum Karlsruhe (http://www.ccdc.cam.ac.uk/structures).

Crystallographic and refinement data are summarized in Table S1.

Table S1. Crystal data and structure refinement for compounds **1-5**,. [(BDI*)MgPPh$_2$·THF] (**4**-THF), [(BDI*)MgP(SiMe$_3$)$_2$] and (BDI*)Li(THF).

Compound	(BDI*)MgMgCp*(**1**)	[(BDI*)MgCp]·0.5toluene (**2**)	(BDI*-H)Mg(H)CaCp* (**3**)
Identification code	hasj211027b	hasj230727b	hasj220720a
Empirical formula	$C_{53}H_{84}Mg_2N_2$	$C_{51.5}H_{78}MgN_2$	$C_{53}H_{84}CaMgN_2$
Formula weight	797.84	749.46	813.61
Temperature/K	100.0(6)	99.98(10)	100.0(2)
Crystal system	monoclinic	monoclinic	monoclinic
Space group	$P2_1/n$	$P2_1/c$	$P2_1$
a/Å	13.18073(8)	16.3155(3)	11.03789(13)
b/Å	17.97025(10)	12.0154(2)	20.26547(16)
c/Å	21.46011(11)	23.8652(5)	11.82050(14)
α/°	90	90	90
β/°	94.1777(5)	98.025(2)	111.9314(13)
γ/°	90	90	90
Volume/Å^3	5069.55(5)	4632.65(15)	2452.75(5)
Z	4	4	2
ρ_{calc}g/cm^3	1.045	1.075	1.102
μ/mm^{-1}	0.662	0.573	1.474
F(000)	1760.0	1652.0	896.0
Crystal size/mm^3	0.628 × 0.479 × 0.383	0.27 × 0.234 × 0.102	0.585 × 0.53 × 0.436
Radiation	Cu Kα (λ = 1.54184)	Cu Kα (λ = 1.54184)	Cu Kα (λ = 1.54184)
2Θ range for data collection/°	6.422 to 145.324	7.482 to 145.24	8.064 to 151.708
Index ranges	-16 ≤ h ≤ 16, -21 ≤ k ≤ 20, -26 ≤ l ≤ 25	-20 ≤ h ≤ 20, -14 ≤ k ≤ 14, -28 ≤ l ≤ 28	-13 ≤ h ≤ 13, -25 ≤ k ≤ 25, -14 ≤ l ≤ 14
Reflections collected	39246	35439	47268
Independent reflections	9917 [R_{int} = 0.0248, R_{sigma} = 0.0188]	8989 [R_{int} = 0.0360, R_{sigma} = 0.0286]	9837 [R_{int} = 0.0258, R_{sigma} = 0.0172]
Data/restraints/parameters	9917/0/534	8989/0/474	9837/1/538
Goodness-of-fit on F^2	1.031	1.025	1.034
Final R indexes [I>=2σ (I)]	R_1 = 0.0354, wR_2 = 0.0901	R_1 = 0.0389, wR_2 = 0.0944	R_1 = 0.0250, wR_2 = 0.0640
Final R indexes [all data]	R_1 = 0.0369, wR_2 = 0.0912	R_1 = 0.0446, wR_2 = 0.0983	R_1 = 0.0257, wR_2 = 0.0646
Largest diff. peak/hole / e Å^{-3}	0.30/-0.19	0.24/-0.27	0.17/-0.17
Flack parameter	-	-	-0.006(2)
CCDC number	2477181	2477183	2477182

Table S1. Crystal data and structure refinement for compounds **1-5**,. [(BDI*)MgPPh$_2$·THF] (**4**-THF), [(BDI*)MgP(SiMe$_3$)$_2$] and (BDI*)Li(THF). (continued).

Compound	[(BDI*)MgPPh$_2$]·0.5Et$_2$O (**4**)	[(BDI*)MgPPh$_2$·THF]·solvent (**4**-THF)	(BDI*)MgP(SiMe$_3$)$_2$
Identification code	hasj240605a	hasj240823b	hasj250505a
Empirical formula	$C_{57}H_{84}MgN_2O_{0.5}P$	$C_{59}H_{87}MgN_2OP$ [a)]	$C_{49}H_{87}MgN_2PSi_2$
Formula weight	860.54	895.58 [a)]	815.66
Temperature/K	99.97(15)	150.00(10)	100.00(10)
Crystal system	triclinic	monoclinic	tetragonal
Space group	P-1	P2$_1$/c	P-42$_1$c
a/Å	10.9610(2)	13.28360(10)	26.0311(2)
b/Å	12.2597(3)	19.0242(2)	26.0311(2)
c/Å	21.3820(5)	23.2965(2)	15.0717(2)
α/°	76.109(2)	90	90
β/°	75.2994(18)	94.4830(10)	90
γ/°	68.585(2)	90	90
Volume/Å^3	2552.43(11)	5869.24(9)	10212.9(2)
Z	2	4	8
ρ_{calc}g/cm^3	1.120	1.014 [a)]	1.061
μ/mm^{-1}	0.875	0.784 [a)]	1.272
F(000)	942.0	1960.0 [a)]	3600.0
Crystal size/mm^3	0.359 × 0.255 × 0.218	0.29 × 0.187 × 0.146	0.64 × 0.44 × 0.27
Radiation	Cu Kα (λ = 1.54184)	Cu Kα (λ = 1.54184)	Cu Kα (λ = 1.54184)
2Θ range for data collection/°	7.852 to 145.144	7.612 to 145.242	7.594 to 143.27
Index ranges	-12 ≤ h ≤ 13, -15 ≤ k ≤ 15, -26 ≤ l ≤ 26	-11 ≤ h ≤ 16, -23 ≤ k ≤ 21, -28 ≤ l ≤ 25	-31 ≤ h ≤ 32, -29 ≤ k ≤ 28, -18 ≤ l ≤ 11
Reflections collected	38396	24573	21582
Independent reflections	9954 [R_{int} = 0.0264, R_{sigma} = 0.0202]	11373 [R_{int} = 0.0253, R_{sigma} = 0.0321]	9061 [R_{int} = 0.0265, R_{sigma} = 0.0306]
Data/restraints/parameters	9954/78/593	11373/0/595	9061/0/516
Goodness-of-fit on F^2	1.029	1.165	1.027
Final R indexes [I>=2σ (I)]	R_1 = 0.0396, wR_2 = 0.1037	R_1 = 0.0549, wR_2 = 0.1327	R_1 = 0.0303, wR_2 = 0.0775
Final R indexes [all data]	R_1 = 0.0423, wR_2 = 0.1057	R_1 = 0.0585, wR_2 = 0.1346	R_1 = 0.0331, wR_2 = 0.0796
Largest diff. peak/hole / e Å^{-3}	0.56/-0.42	0.24/-0.23	0.21/-0.18
Flack parameter	-	-	-0.003(9)
CCDC number	2477184	2477185	2477186

a) Contribution of the masked disordered solvent neglected.

Table S1. Crystal data and structure refinement for compounds **1-5**,. [(BDI*)MgPPh$_2$·THF] (**4**-THF), [(BDI*)MgP(SiMe$_3$)$_2$] and (BDI*)Li(THF). (continued).

Compound	(BDI*)Li(THF)	(BDI*)MgMgOAr·THF (**5**)
Identification code	hasj250505b	hasj240216a_twin1_hklf5
Empirical formula	$C_{47}H_{77}LiN_2O$	$C_{83}H_{130}Mg_2N_2O_2$
Formula weight	693.04	1236.50
Temperature/K	100.00(10)	100.00(12)
Crystal system	monoclinic	triclinic
Space group	Pn	P-1
a/Å	10.41979(16)	17.7343(6)
b/Å	28.2993(4)	19.0137(5)
c/Å	14.8301(2)	22.6961(8)
α/°	90	103.556(3)
β/°	90.6542(14)	93.741(3)
γ/°	90	91.090(2)
Volume/Å^3	4372.72(11)	7419.3(4)
Z	4	4
ρ_{calc}g/cm^3	1.053	1.107
μ/mm^{-1}	0.451	0.632
F(000)	1536.0	2728.0
Crystal size/mm^3	0.446 × 0.153 × 0.141	0.205 × 0.131 × 0.082
Radiation	Cu Kα (λ = 1.54184)	Cu Kα (λ = 1.54184)
2Θ range for data collection/°	8.638 to 143.926	7.332 to 145.176
Index ranges	-12 ≤ h ≤ 12, -34 ≤ k ≤ 34, -18 ≤ l ≤ 15	-17 ≤ h ≤ 21, -23 ≤ k ≤ 23, -27 ≤ l ≤ 28
Reflections collected	63590	32350
Independent reflections	14447 [R_{int} = 0.0475, R_{sigma} = 0.0377]	32350 [R_{int} = 0.058, R_{sigma} = 0.0509]
Data/restraints/parameters	14447/2/948	32350/6421/2175
Goodness-of-fit on F^2	1.039	1.035
Final R indexes [I>=2σ (I)]	R_1 = 0.0598, wR_2 = 0.1696	R_1 = 0.0775, wR_2 = 0.1848
Final R indexes [all data]	R_1 = 0.0613, wR_2 = 0.1707	R_1 = 0.0990, wR_2 = 0.1959
Largest diff. peak/hole / e Å^{-3}	0.30/-0.32	0.32/-0.83
Flack parameter	0.3(4)	-
CCDC number	2477188	2477187

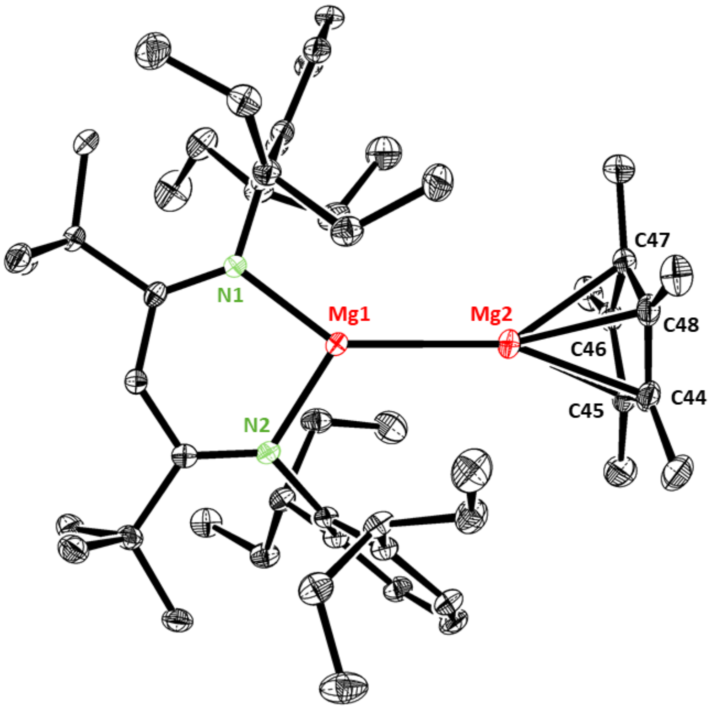

Figure S36. Molecular structure of (BDI*)MgMgCp*(**1**). Ellipsoids represent 50% probability. Hydrogen atoms have been omitted for clarity.

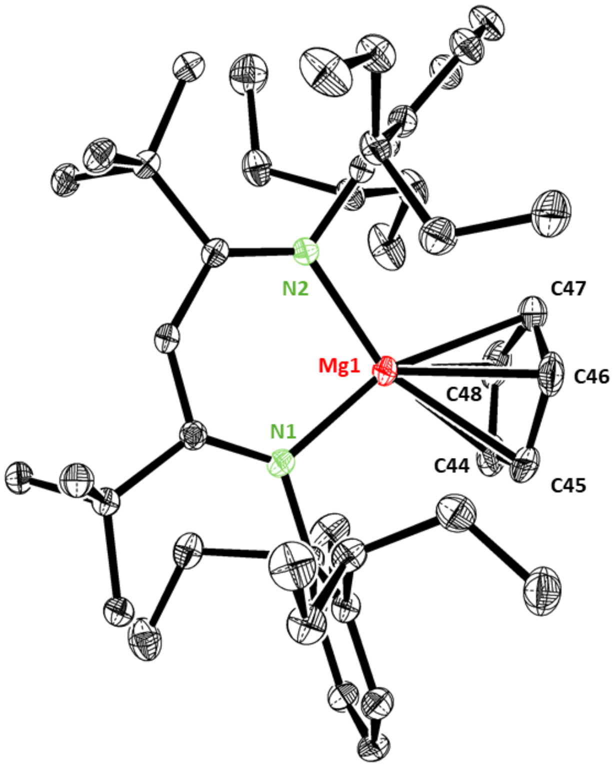

Figure S37. Molecular structure of (BDI*)MgCp (**2**). Ellipsoids represent 50% probability. Hydrogen atoms and co-crystallized toluene have been omitted for clarity.

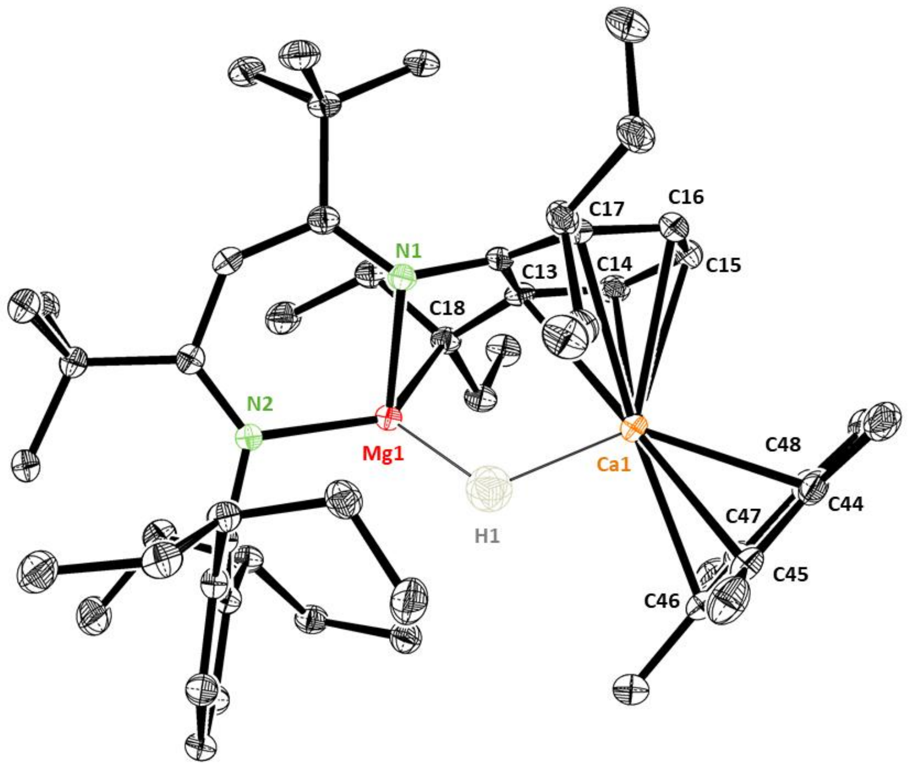

Figure S38. Molecular structure of (BDI*-H)Mg(H)CaCp* (**3**). Ellipsoids represent 50% probability. Hydrogen atoms have been omitted for clarity, except for the hydride H which was found and isotropically refined.

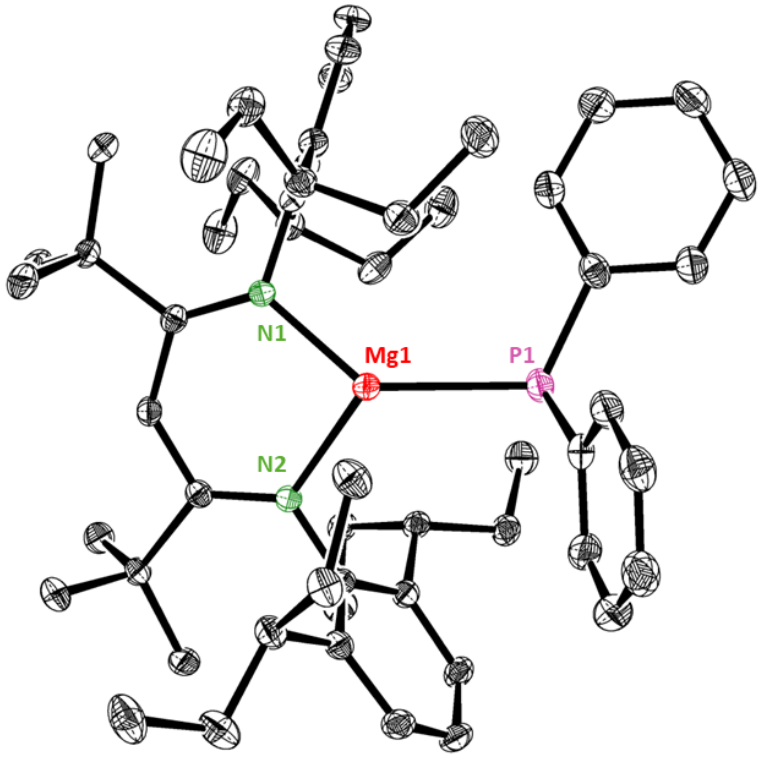

Figure S39. Molecular structure of (BDI*)$MgPPh_2$ (**4**). Ellipsoids represent 50% probability. Hydrogen atoms and co-crystallized Et_2O have been omitted for clarity.

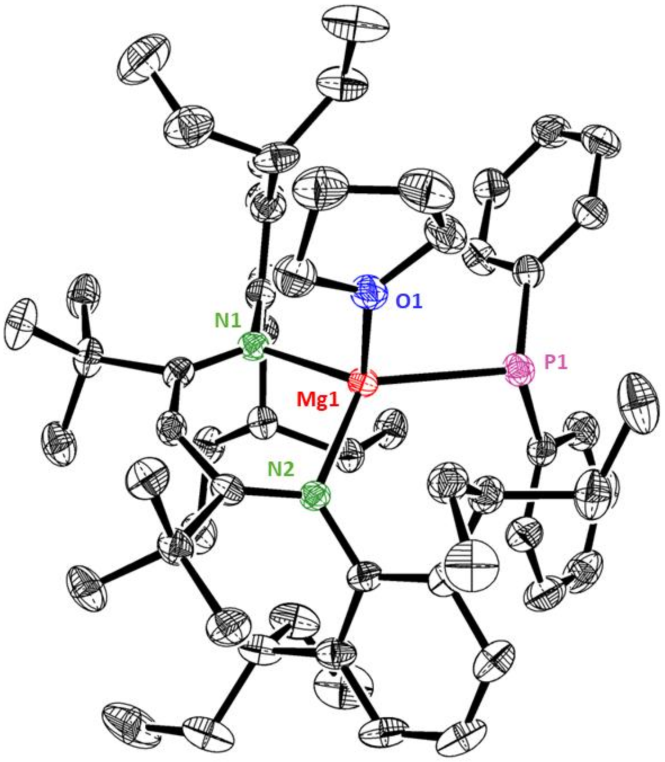

Figure S40. Molecular structure of [(BDI*)MgPPh$_2$·THF] (**4**-THF). Ellipsoids represent 50% probability. Hydrogen atoms and co-crystallized solvent have been omitted for clarity.

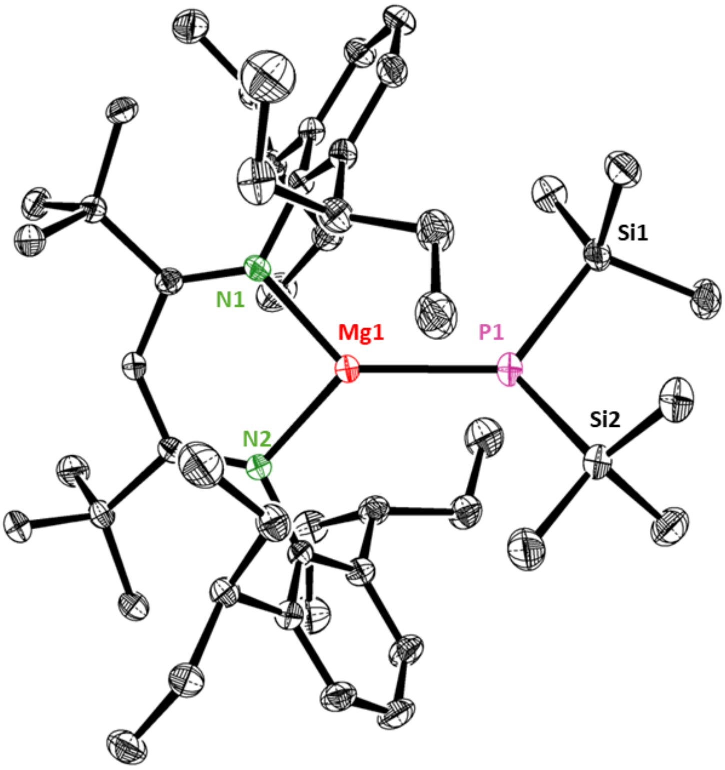

Figure S41. Molecular structure of (BDI*)Mg(SiMe$_3$)$_2$. Ellipsoids represent 50% probability. Hydrogen atoms have been omitted for clarity.

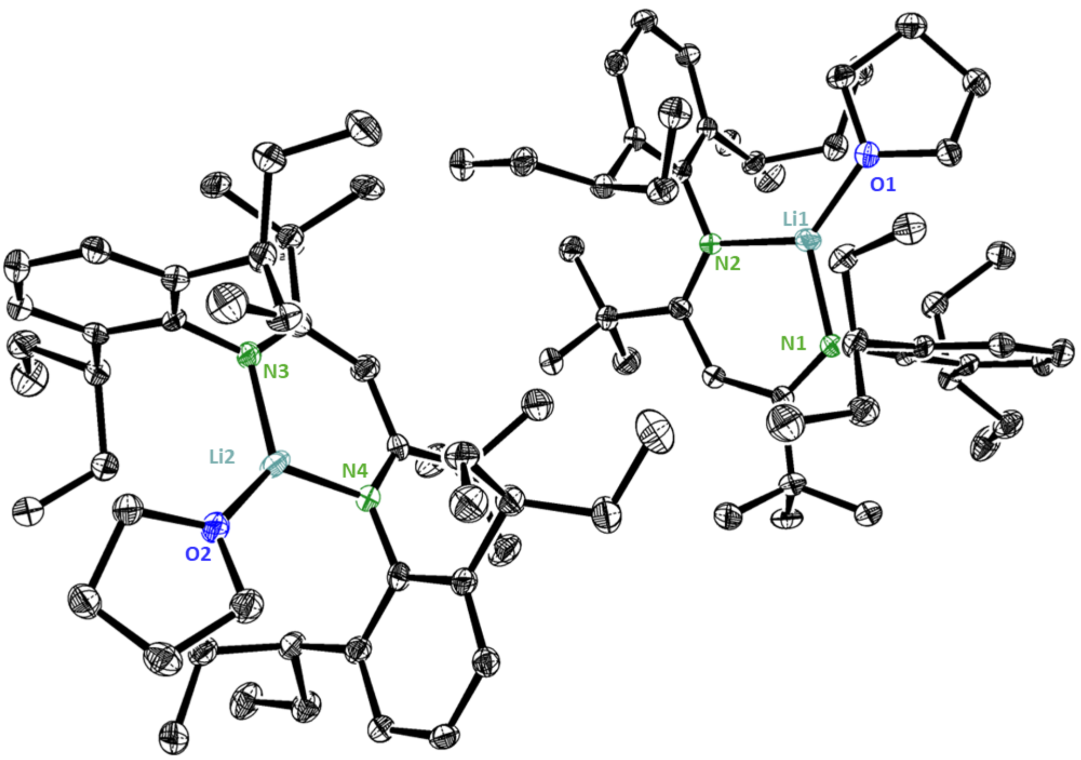

Figure S42. Molecular structure of (BDI*)Li(THF). Ellipsoids represent 50% probability. Hydrogen atoms have been omitted for clarity.

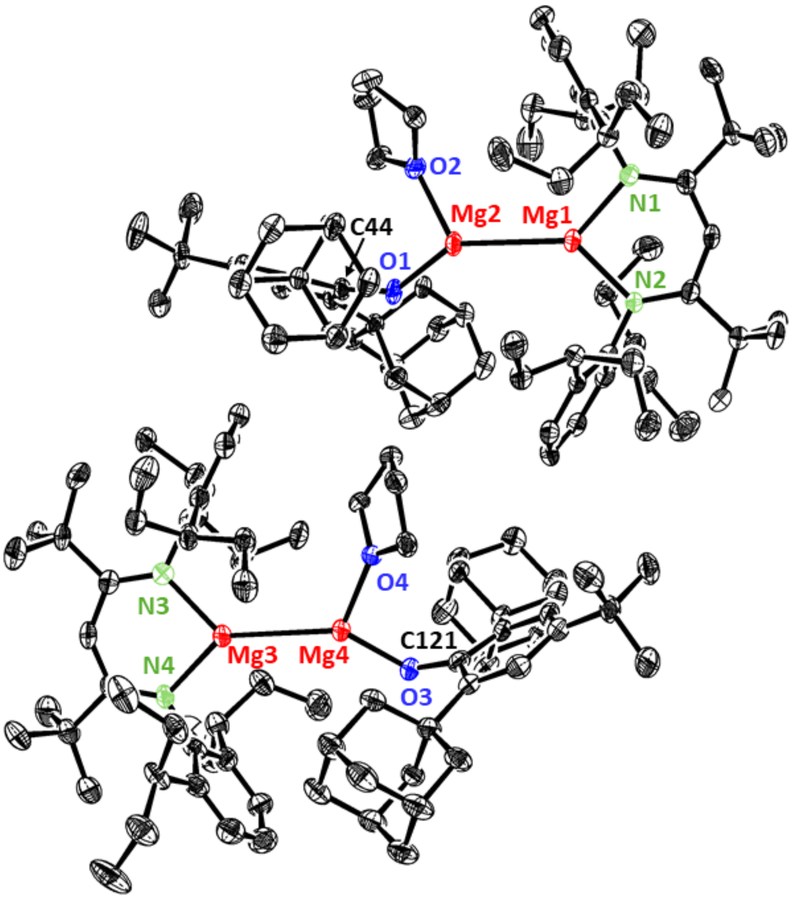

Figure S43. Molecular structure of (BDI*)MgMgOAr·THF (**5**).Ellipsoids represent 50% probability. Disorder, hydrogen atoms and co-crystallized cyclohexene have been omitted for clarity.

5. DFT Calculation

All calculations were carried out using Gaussian 16A.[S18] All methods were used as implemented. All structures were fully optimized at a B3PW91/def2SVP level of theory.[S19–S22] All structures were characterized as true minima (Nimag=0) or as transition states (Nimag=1) by frequency calculations on the same level of theory. Energies were determined at a B3PW91/def2TZVP level of theory. In all cases Grimme`s third dispersion correction with Becke-Johnson damping (GD3BJ) was applied.[S23] Charges were calculated with NBO7 analyses using the B3PW91/def2TZVP level of theory.[S24] All structures were evaluated using Molecule 2.3.[S25] The QTAIM analysis was calculated using the B3PW91-D3BJ/def2tzvp wave function with AIMAll (v17).[S26,S27]

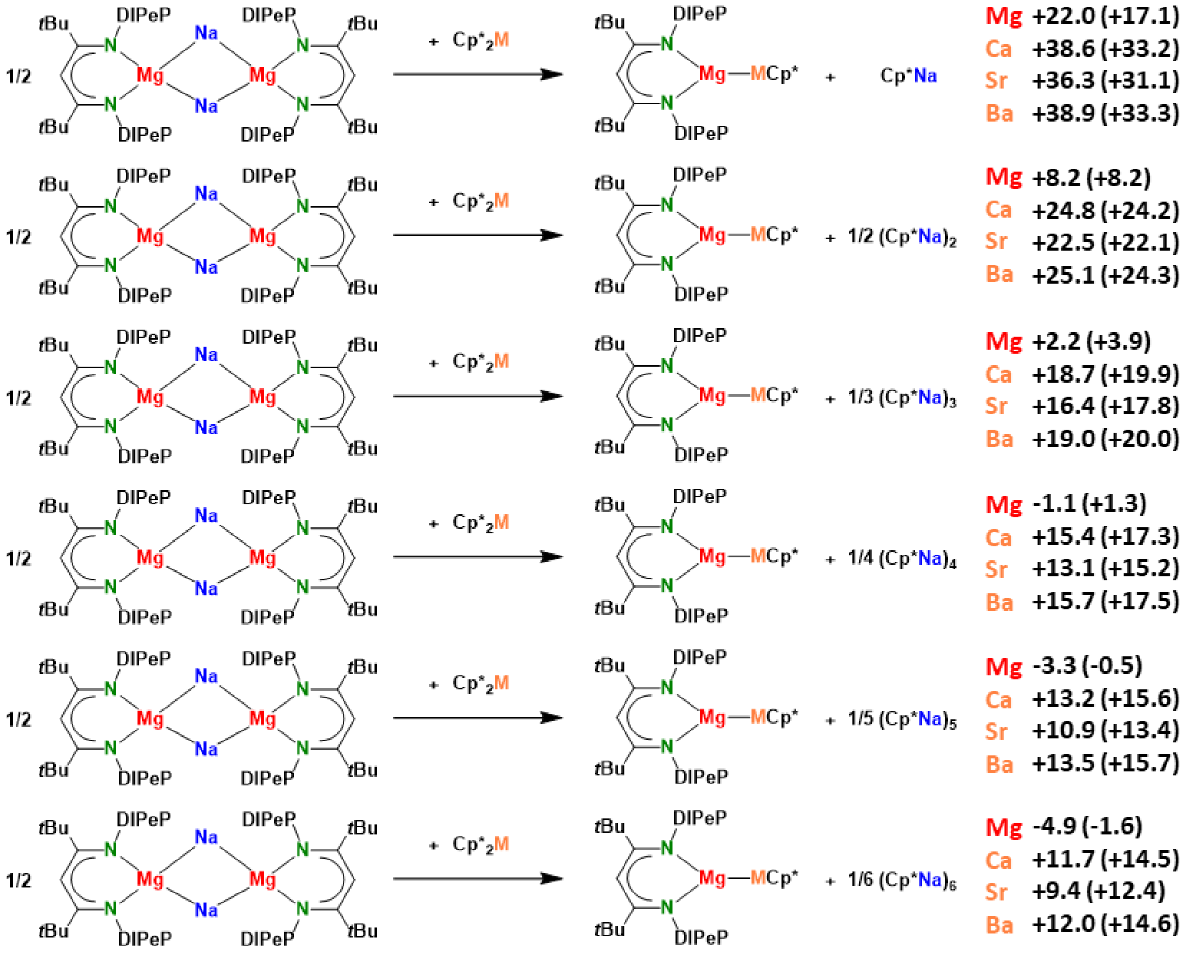

Figure S44. Reaction enthalpies (ΔH) and between brackets free energies (ΔG at 298 K) in kcal·mol^{-1}.

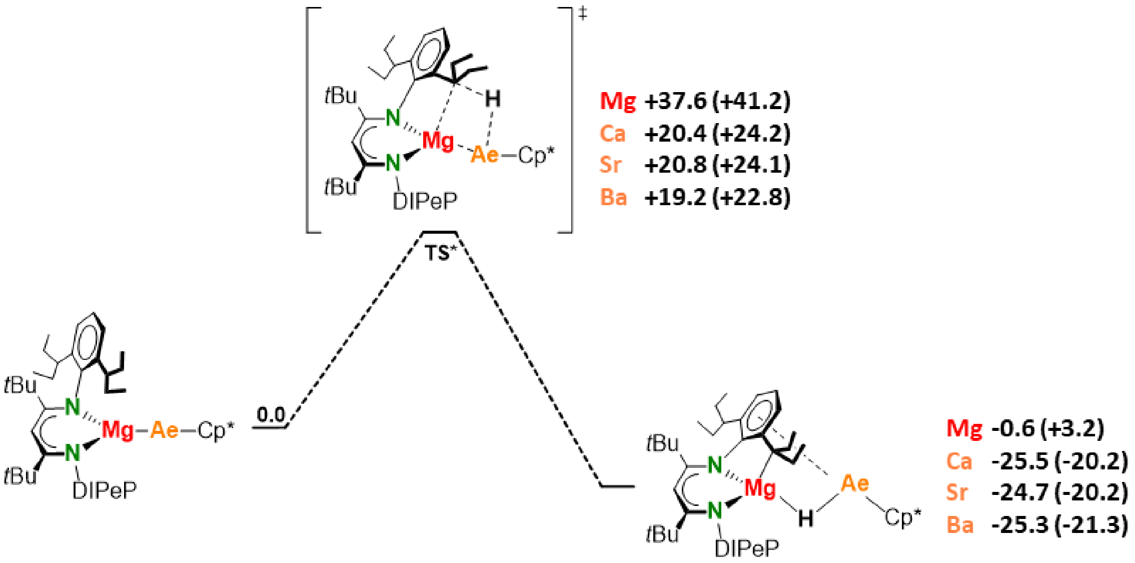

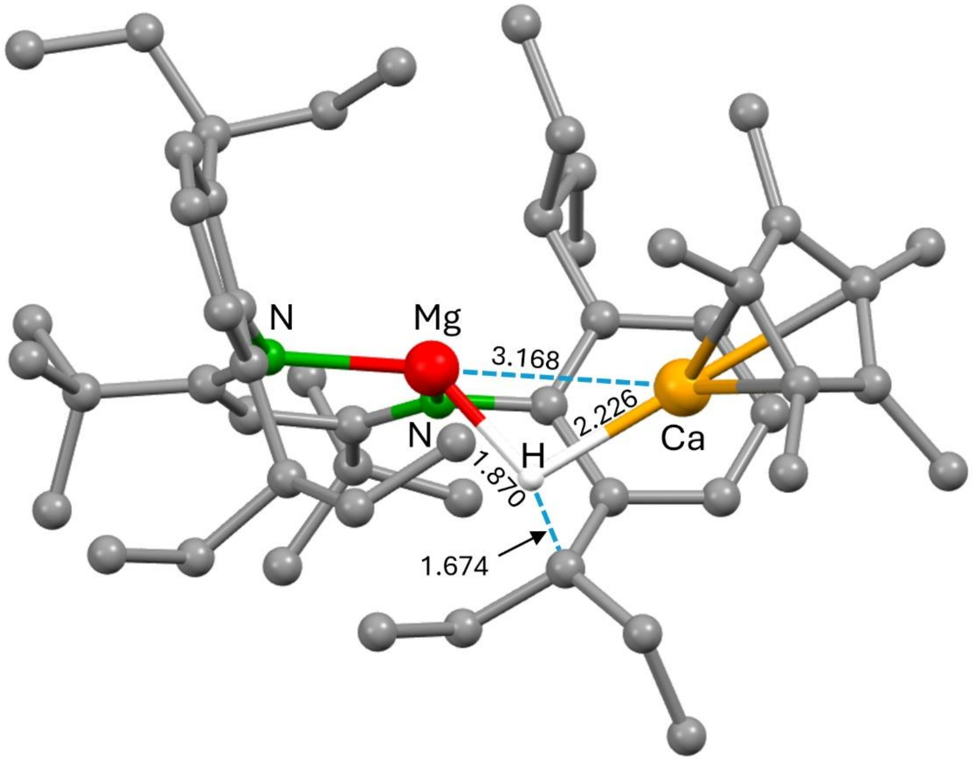

Figure S45. (top) Energy profiles for intramolecular C–H bond activation in [(BDI*)MgAeCp*] to give [(BDI*-H)Mg(H)AeCp*] (Ae = Mg, Ca, Sr, Ba). Reaction enthalpies (ΔH) and between brackets free energies (ΔG at 298 K) in kcal·mol^{-1}. (bottom) The transition state for decomposition of (BDI*)MgCaCp* (selected distances in Å).

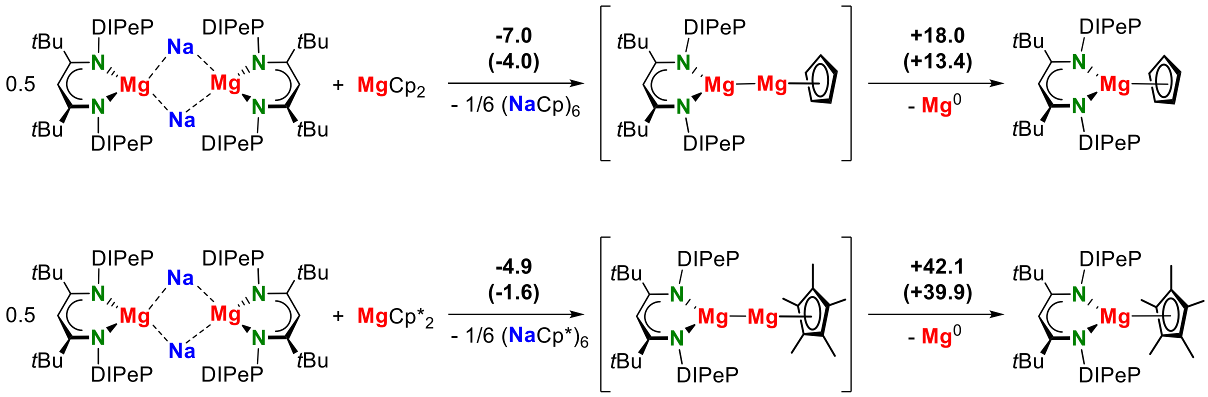

Figure S46. Calculated energies for the formation of (BDI*)MgMgCp*(**1**) and (BDI*)MgMgCp and decomposition to [(BDI*)MgCp*] and (BDI*)MgCp (**2**), respectively. Reaction enthalpies (ΔH) and between brackets free energies (ΔG at 298 K) in kcal·mol^{-1}.

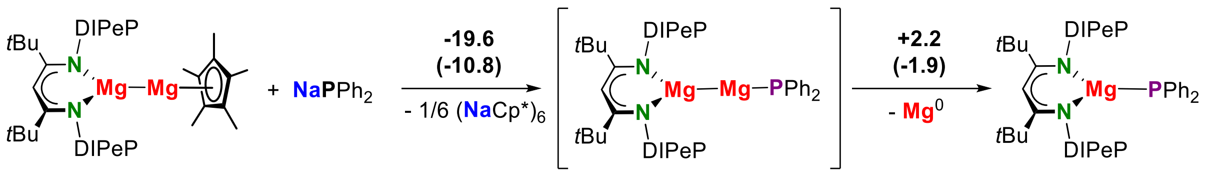

Figure S47. Calculated energies for the formation of (BDI*)MgMgPPh$_2$ and decomposition to (BDI*)MgPPh$_2$ (**4**). Reaction enthalpies (ΔH) and between brackets free energies (ΔG at 298 K) in kcal·mol^{-1}.

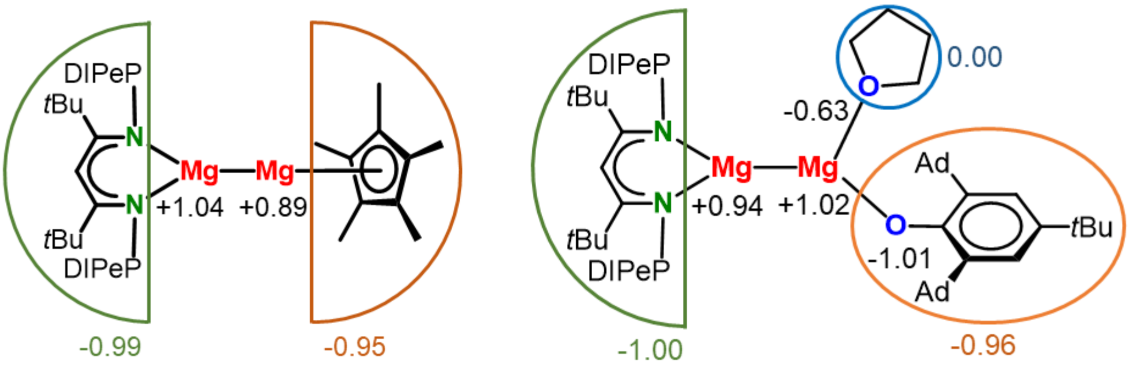

Figure S48. Calculated NPA charges for (BDI*)MgMgCp*(**1**) and (BDI*)MgMgOAr·THF (**5**).

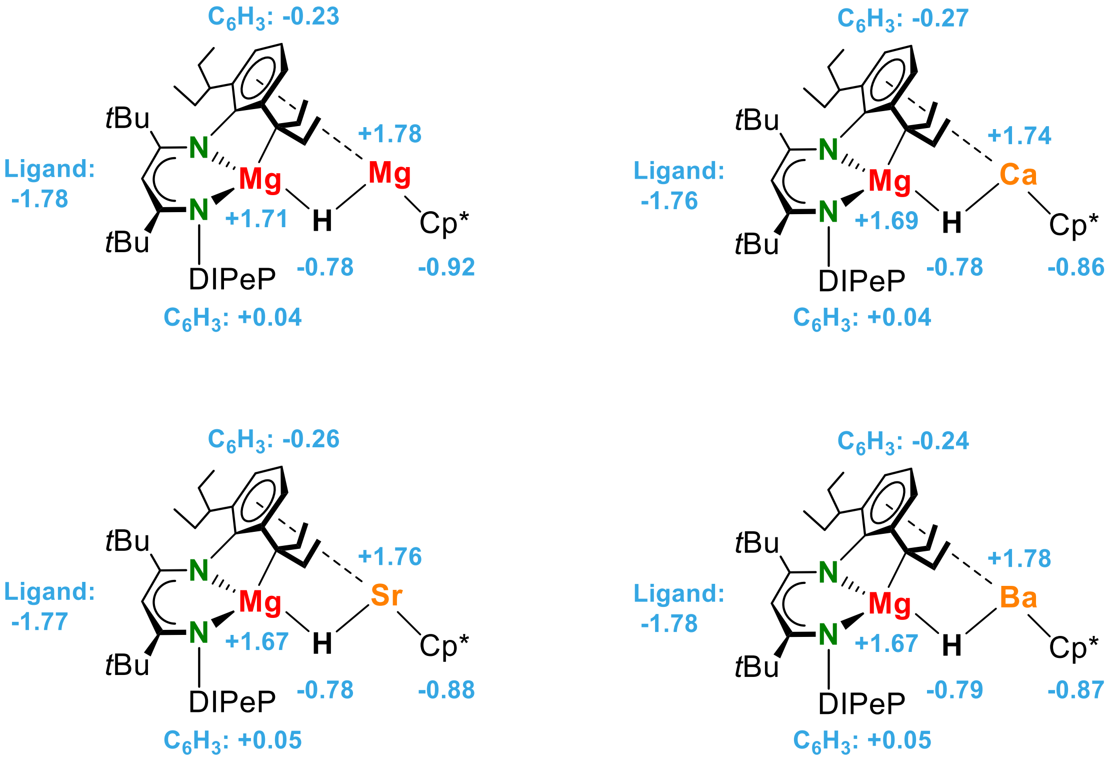

Figure S49. Calculated NPA charges for (BDI*-H)Mg(H)AeCp* (Ae = Mg, Ca, Sr, Ba).

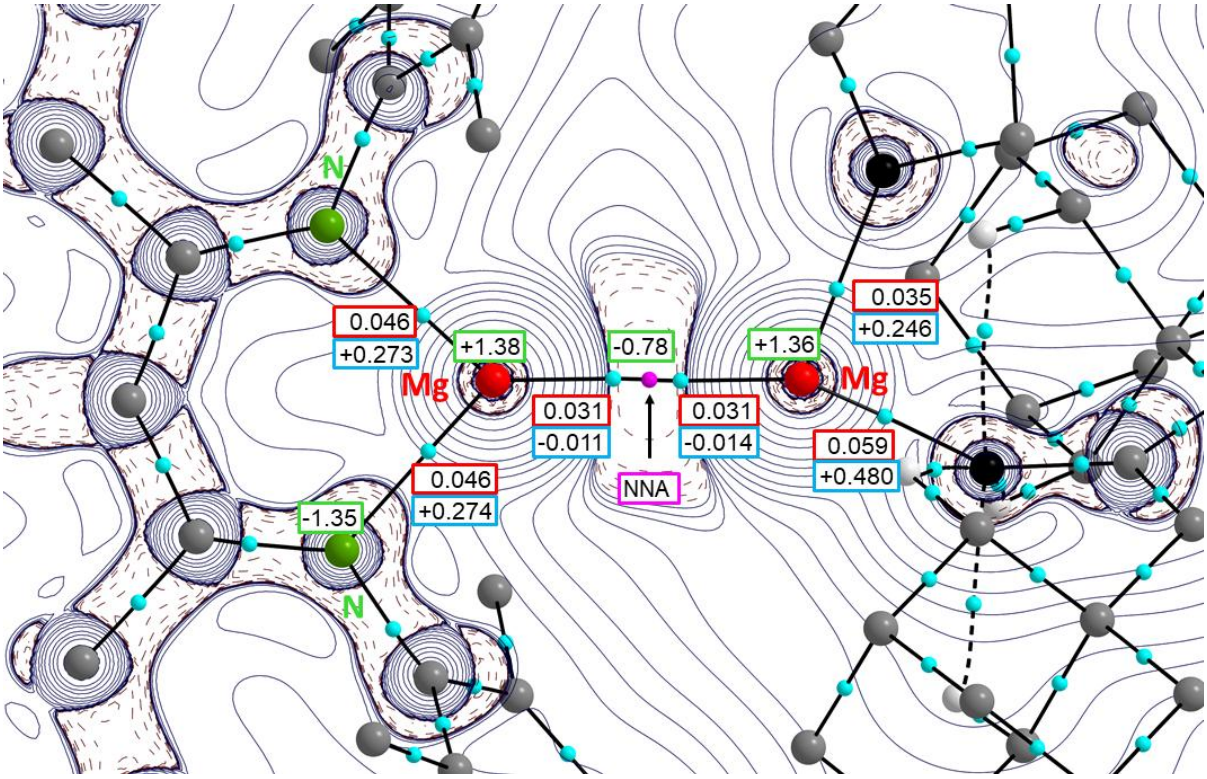

Figure S50. Atoms-In-Molecules analysis for (BDI*)MgMgOAr·THF (**5**) showing the Laplacian distribution with bond-critical-points (bcp's) as light-blue dots and AIM charges in green boxes. The electron density ρ(r) in e B¯3 and Laplacian ∇2ρ(r) in e B¯5 are shown in red and blue boxes, respectively. Non-nuclear attractor (NNA) in pink.

Red dashed lines indicate areas of charge concentration, while dark-blue solid lines show areas of charge depletion. DIPeP-groups and hydrogen atoms were omitted for clarity.

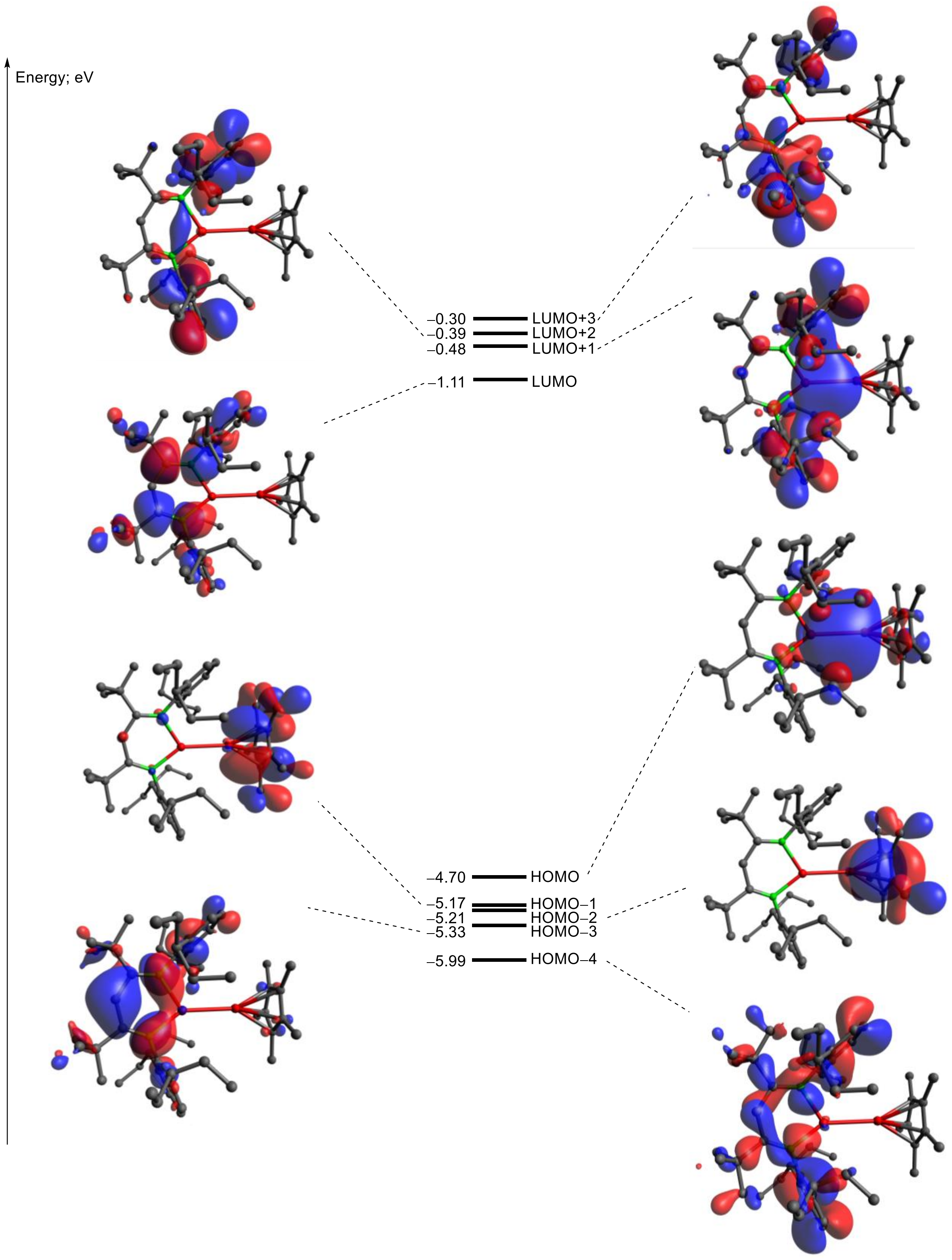

Figure S51. Kohn-Sham Molecular Orbital Diagram of (^{DIPeP}BDI*)MgMgCp* (**1**).

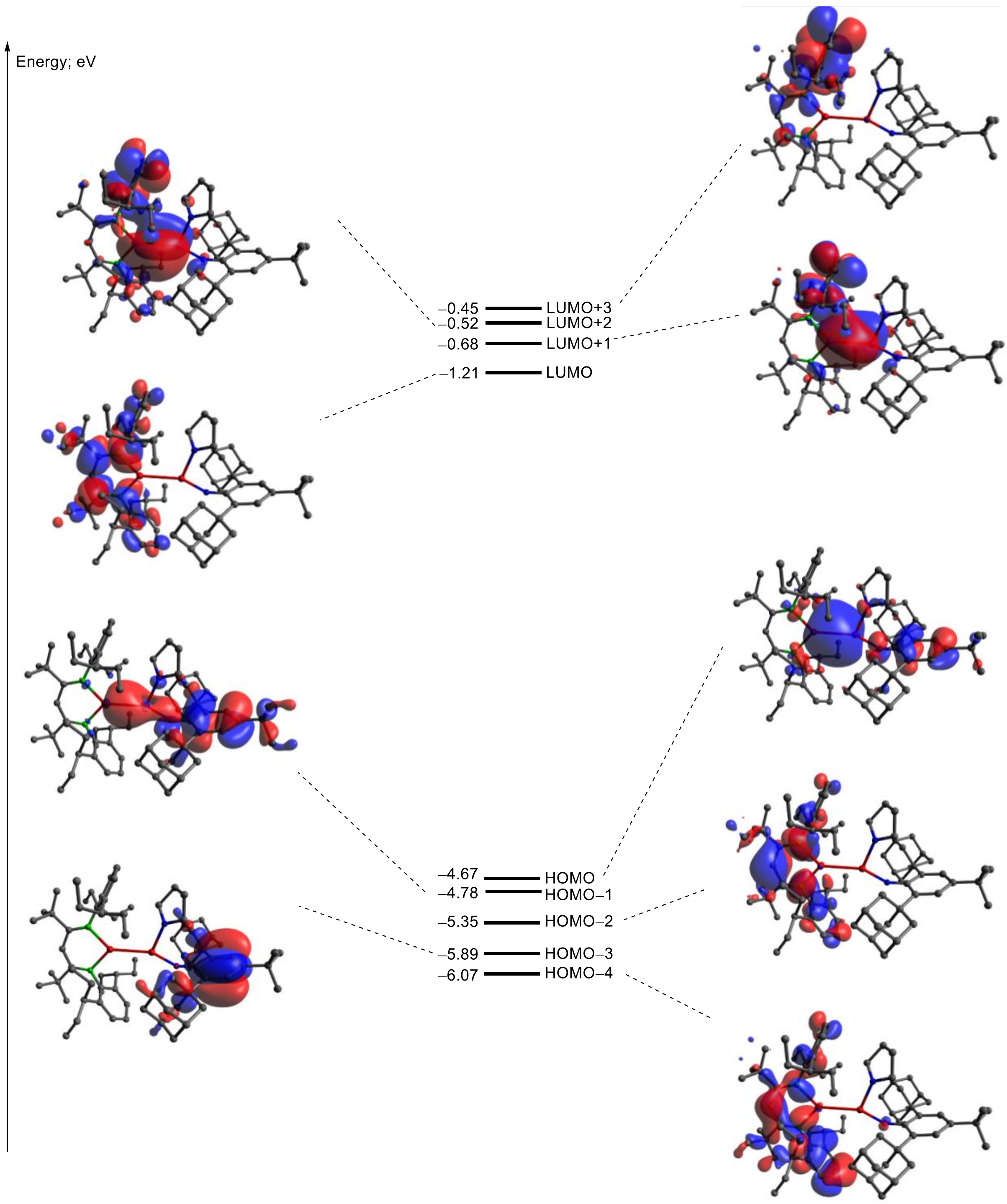

Figure S52. Kohn-Sham Molecular Orbital Diagram of (BDI*)MgMgOAr·THF (**5**).

XYZ-coordinates

[(BDI*)MgNa]$_2$ (**III**)

232

Mg	2.832345	0.013376	-0.010365
Na	-0.197787	1.532796	-0.204244
N	3.945456	1.846643	-0.001690
N	4.667782	-1.075708	-0.049670
C	5.266258	1.958573	-0.028262
C	6.117463	0.833144	-0.044068
H	7.168791	1.093978	-0.047682
C	5.889321	-0.562595	-0.040854
C	6.046928	3.312597	-0.001545
C	5.191963	4.585113	-0.010294
H	4.627484	4.708228	-0.941098
H	5.864649	5.451723	0.085474
H	4.476534	4.629316	0.818149
C	6.974953	3.396360	-1.227585
H	7.721894	2.592045	-1.250762
H	7.514998	4.356358	-1.219916
H	6.398291	3.344184	-2.163002
C	6.896390	3.345808	1.284512
H	6.262548	3.246925	2.178025
H	7.434801	4.304407	1.353609
H	7.639546	2.538200	1.315916
C	7.218646	-1.391416	-0.053064
C	8.045413	-1.052406	1.201338
H	8.306743	0.012804	1.258214
H	8.983112	-1.630309	1.193457
H	7.501303	-1.313664	2.119835
C	8.035538	-0.996171	-1.301008
H	7.479615	-1.203236	-2.226627
H	8.968307	-1.581058	-1.330552
H	8.307186	0.067413	-1.309079
C	7.061769	-2.916022	-0.089492
H	6.618079	-3.318550	0.827138
H	8.062522	-3.362545	-0.198591
H	6.451275	-3.262569	-0.930171
C	2.962263	2.842952	-0.106855
C	2.186173	3.178003	1.038153
C	1.088786	4.040316	0.879069
H	0.484290	4.308644	1.747359
C	0.733840	4.543500	-0.372502
H	-0.131888	5.200781	-0.472585
C	1.481934	4.180733	-1.493575
H	1.193409	4.566868	-2.472738
C	2.587886	3.329693	-1.388754
C	2.536852	2.619716	2.406293
H	3.189686	1.748931	2.226973
C	3.382044	3.602653	3.239174
H	3.724626	3.076225	4.144967
H	4.297471	3.830776	2.673501
C	2.697455	4.903588	3.631889
H	3.357308	5.516545	4.265145
H	1.770552	4.725418	4.199954
H	2.435281	5.505883	2.748859
C	1.296464	2.097420	3.146824
H	0.530050	2.883746	3.225817
H	0.851025	1.301721	2.520765
C	1.575538	1.543421	4.535740
H	1.858518	2.332842	5.248632
H	2.390737	0.805335	4.516555
H	0.683483	1.043798	4.941991
C	3.353001	2.888849	-2.621926
H	4.359059	2.598899	-2.286528
C	3.538039	4.014636	-3.647011
H	2.563536	4.335278	-4.050447
H	3.943599	4.895836	-3.121442
C	4.456930	3.644903	-4.803333
H	5.435635	3.291996	-4.439678
H	4.637986	4.510216	-5.458989
H	4.031169	2.844413	-5.427410
C	2.739285	1.609338	-3.221457
H	3.434337	1.195670	-3.969922
H	2.681313	0.846657	-2.421432
C	1.354157	1.763314	-3.822904
H	1.338973	2.471159	-4.666194
H	0.628738	2.120952	-3.077024
H	0.978436	0.799109	-4.194584
C	4.264098	-2.414721	0.115980
C	4.251417	-2.994541	1.410095
C	3.731994	-4.282226	1.567555
H	3.736925	-4.744551	2.556037
C	3.200062	-4.984753	0.490028
H	2.806863	-5.994442	0.631069
C	3.155976	-4.383117	-0.763942
H	2.714156	-4.927841	-1.599260
C	3.663344	-3.096104	-0.975577
C	4.732177	-2.200822	2.608838
H	5.456982	-1.460675	2.242192
C	3.577899	-1.374853	3.206097
H	3.987248	-0.703206	3.978214
H	3.185673	-0.707197	2.415415
C	2.413477	-2.173343	3.764669
H	2.721636	-2.849116	4.578119
H	1.943547	-2.782087	2.977759
H	1.640784	-1.502190	4.165920
C	5.467869	-3.056595	3.647487
H	4.787148	-3.804288	4.086239
H	6.245666	-3.637685	3.123416
C	6.105879	-2.250396	4.771006

H	6.768643	-1.463692	4.375248
H	6.710386	-2.894651	5.427566
H	5.352037	-1.756352	5.402681
C	3.572856	-2.433785	-2.338485
H	3.345696	-1.367955	-2.143523
C	4.918461	-2.445314	-3.079731
H	4.845295	-1.794367	-3.966306
H	5.657341	-1.962150	-2.427422
C	5.412256	-3.825167	-3.488280
H	6.422011	-3.771743	-3.923791
H	4.755701	-4.291521	-4.239526
H	5.456548	-4.506301	-2.623561
C	2.414523	-2.964527	-3.187326
H	2.572738	-4.025846	-3.440283
H	1.499212	-2.941239	-2.570067
C	2.179974	-2.177775	-4.467361
H	3.032410	-2.252948	-5.159330
H	2.021743	-1.111210	-4.248940
H	1.291347	-2.544497	-5.001477
Mg	-2.832285	-0.013360	-0.009991
Na	0.197810	-1.533250	-0.204134
N	-3.945442	-1.846610	-0.002022
N	-4.667776	1.075637	-0.049774
C	-5.266236	-1.958582	-0.029070
C	-6.117464	-0.833184	-0.045072
H	-7.168789	-1.094034	-0.049078
C	-5.889326	0.562559	-0.041527
C	-6.046891	-3.312630	-0.002751
C	-5.191935	-4.585156	-0.011353
H	-4.627232	-4.708233	-0.942027
H	-5.864663	-5.451758	0.084203
H	-4.476712	-4.629423	0.817264
C	-6.974508	-3.396293	-1.229105
H	-7.721426	-2.591961	-1.252493
H	-7.514588	-4.356273	-1.221674
H	-6.397526	-3.344081	-2.164323
C	-6.896772	-3.345960	1.283028
H	-6.263224	-3.247072	2.176750
H	-7.435143	-4.304596	1.351911
H	-7.639984	-2.538396	1.314223
C	-7.218640	1.391385	-0.053974
C	-8.045663	1.052230	1.200223
H	-8.307011	-0.012985	1.256907
H	-8.983356	1.630142	1.192230
H	-7.501732	1.313364	2.118860
C	-8.035285	0.996286	-1.302126
H	-7.479180	1.203449	-2.227614
H	-8.968039	1.581191	-1.331787
H	-8.306948	-0.067292	-1.310372
C	-7.061757	2.915993	-0.090191
H	-6.618364	3.318426	0.826625

H	-8.062478	3.362524	-0.199565
H	-6.450993	3.262633	-0.930636
C	-2.962248	-2.842935	-0.107028
C	-2.186506	-3.178195	1.038147
C	-1.089192	-4.040643	0.879272
H	-0.484969	-4.309134	1.747698
C	-0.733996	-4.543780	-0.372245
H	0.131652	-5.201194	-0.472161
C	-1.481715	-4.180765	-1.493496
H	-1.192988	-4.566865	-2.472614
C	-2.587550	-3.329547	-1.388895
C	-2.537471	-2.619989	2.406246
H	-3.190174	-1.749125	2.226829
C	-3.382974	-3.602888	3.238847
H	-3.725745	-3.076472	4.144576
H	-4.298281	-3.830887	2.672921
C	-2.698623	-4.903917	3.631666
H	-3.358677	-5.516808	4.264775
H	-1.771811	-4.725878	4.199922
H	-2.436346	-5.506214	2.748669
C	-1.297204	-2.097882	3.147107
H	-0.530925	-2.884321	3.226266
H	-0.851504	-1.302241	2.521159
C	-1.576529	-1.543870	4.535963
H	-1.859885	-2.333248	5.248752
H	-2.391547	-0.805588	4.516587
H	-0.684463	-1.044474	4.942477
C	-3.352157	-2.888301	-2.622235
H	-4.358269	-2.598208	-2.287124
C	-3.537047	-4.013787	-3.647668
H	-2.562456	-4.334505	-4.050834
H	-3.942976	-4.895047	-3.122484
C	-4.455445	-3.643548	-4.804218
H	-5.434225	-3.290590	-4.440817
H	-4.636401	-4.508625	-5.460214
H	-4.029318	-2.842930	-5.427881
C	-2.737927	-1.608775	-3.221213
H	-3.432768	-1.194528	-3.969554
H	-2.679690	-0.846452	-2.420856
C	-1.352813	-1.763005	-3.822637
H	-1.337959	-2.469931	-4.666706
H	-0.627721	-2.121952	-3.077066
H	-0.976406	-0.798630	-4.193172
C	-4.264158	2.414654	0.116087
C	-4.251824	2.994375	1.410246
C	-3.732498	4.282074	1.567933
H	-3.737698	4.744325	2.556450
C	-3.200333	4.984702	0.490589
H	-2.807205	5.994395	0.631802
C	-3.155908	4.383161	-0.763414
H	-2.713912	4.927968	-1.598584

C -3.663160 3.096139 -0.975271
C -4.732867 2.200558 2.608812
H -5.457474 1.460339 2.241916
C -3.578668 1.374719 3.206410
H -3.988152 0.703064 3.978450
H -3.186183 0.707061 2.415853
C -2.414469 2.173356 3.765235
H -2.722942 2.849238 4.578473
H -1.944321 2.782002 2.978382
H -1.641863 1.502318 4.166852
C -5.468975 3.056208 3.647262
H -4.788481 3.803981 4.086231
H -6.246697 3.637210 3.122983
C -6.107195 2.249892 4.770578
H -6.769652 1.463036 4.374607
H -6.712069 2.894027 5.426919
H -5.353463 1.756024 5.402524
C -3.572276 2.433923 -2.338203
H -3.345106 1.368095 -2.143258
C -4.917688 2.445435 -3.079797
H -4.844279 1.794495 -3.966358
H -5.656729 1.962256 -2.427688
C -5.411397 3.825284 -3.488472
H -6.421089 3.771863 -3.924127
H -4.754723 4.291600 -4.239634
H -5.455808 4.506447 -2.623779
C -2.413766 2.964823 -3.186705
H -2.571986 4.026162 -3.439580
H -1.498591 2.941537 -2.569243
C -2.178873 2.178238 -4.466782
H -3.031157 2.253439 -5.158936
H -2.020616 1.111657 -4.248464
H -1.290151 2.545088 -5.000651

$MgCp^*_2$
51
Mg -0.000148 0.000761 0.001253
C -1.968564 0.988488 0.710476
C -1.968082 0.981537 -0.719787
C -1.968677 -0.380757 -1.155123
C -1.969648 -1.215941 0.005832
C -1.969241 -0.369731 1.158840
C -2.010048 2.203658 1.584564
H -1.515159 2.029314 2.551912
H -3.046636 2.513280 1.805276
H -1.512588 3.064519 1.112564
C -2.008369 2.188314 -1.605495
H -3.044652 2.495079 -1.831454
H -1.511337 2.005082 -2.570296
C -2.007285 -0.849897 -2.576807
H -1.511574 -0.139677 -3.255775
H -3.042929 -0.971695 -2.939611
H -1.508666 -1.823198 -2.700050
C -2.011292 -2.712973 0.012826
H -1.516396 -3.140363 -0.872370
H -3.047827 -3.093229 0.016185
H -1.513844 -3.132234 0.900486
C -2.009879 -0.825980 2.584469
H -1.521926 -1.803783 2.714619
H -3.046036 -0.932632 2.950515
H -1.504511 -0.115599 3.256277
H -1.512228 3.053768 -1.140759
C 1.969203 0.435354 1.135680
C 1.969242 1.214297 -0.063779
C 1.968264 0.314151 -1.175262
C 1.967673 -1.021045 -0.662680
C 1.968255 -0.946128 0.765546
C 2.011183 0.971937 2.533118
H 1.509754 0.299230 3.245343
H 1.520145 1.953834 2.609420
H 3.047790 1.102668 2.889915
C 2.012122 2.709124 -0.142079
H 1.514541 3.178900 0.719897
H 1.518231 3.086136 -1.050355
C 2.008510 0.701280 -2.621335
H 1.510321 1.666020 -2.800738
H 1.513320 -0.046335 -3.259330
H 3.044705 0.801556 -2.989150
C 2.006576 -2.276595 -1.477614
H 1.510855 -2.147671 -2.451674
H 1.508131 -3.112882 -0.964434
H 3.042488 -2.597840 -1.684335
C 2.009563 -2.109970 1.707010
H 3.045839 -2.412634 1.938250
H 1.503829 -2.993198 1.288137
H 1.522039 -1.878138 2.666012
H 3.049055 3.087864 -0.159906

$CaCp^*_2$
51
Ca 0.000429 0.002020 -0.437210
C 2.347419 -1.100699 -0.444531
C 2.496270 0.141503 -1.128182
C 2.291265 1.189592 -0.183478
C 2.017642 0.595016 1.082251
C 2.051972 -0.820042 0.920745
C 2.546944 -2.465350 -1.032770
H 1.911165 -3.223251 -0.547725
H 3.589786 -2.811749 -0.918891
H 2.325045 -2.490720 -2.111454
C 2.890564 0.314880 -2.564687
H 3.987843 0.355498 -2.685716

H 2.494655 1.248245 -2.995926
C 2.416657 2.659303 -0.452291
H 2.172395 2.913898 -1.495841
H 3.444237 3.022916 -0.272742
H 1.755129 3.256579 0.195159
C 1.807027 1.333945 2.368439
H 1.304255 2.301069 2.212819
H 2.763254 1.551455 2.876700
H 1.194104 0.758013 3.078021
C 1.883744 -1.839585 2.005880
H 1.311186 -1.440967 2.855900
H 2.857125 -2.176834 2.404377
H 1.358734 -2.742151 1.653371
H 2.537714 -0.515508 -3.197062
C -2.228315 -1.212589 0.095235
C -2.478555 -0.423655 -1.065606
C -2.399946 0.949499 -0.688970
C -2.101101 1.007813 0.703062
C -1.996630 -0.327835 1.187577
C -2.270666 -2.709236 0.172282
H -1.584750 -3.103111 0.938833
H -1.999398 -3.182451 -0.784696
H -3.278967 -3.078756 0.431172
C -2.849482 -0.944771 -2.422060
H -2.403110 -1.931197 -2.625781
H -2.532411 -0.266685 -3.230604
C -2.668291 2.125897 -1.579220
H -2.415962 1.919073 -2.631562
H -2.097910 3.017228 -1.272815
H -3.734223 2.415676 -1.564247
C -1.988101 2.253781 1.528174
H -1.573983 3.097881 0.953820
H -1.340020 2.106582 2.405140
H -2.970977 2.583941 1.909123
C -1.769231 -0.732247 2.611836
H -2.719602 -0.815333 3.168602
H -1.147436 -0.003407 3.153733
H -1.269478 -1.709609 2.688694
H -3.942119 -1.068052 -2.528327

$SrCp^*_2$
51
Sr -0.000157 0.000271 -0.543946
C -2.559391 1.045254 -0.465323
C -2.714457 -0.259554 -1.016355
C -2.424755 -1.206199 0.008475
C -2.093044 -0.486896 1.192363
C -2.175272 0.904849 0.899237
C -2.836365 2.340435 -1.168894
H -2.216386 3.165244 -0.781547
H -3.887443 2.659255 -1.047981
H -2.656147 2.274363 -2.254363
C -3.187790 -0.580574 -2.402862
H -4.288174 -0.667903 -2.453328
H -2.785125 -1.538439 -2.771128
C -2.531445 -2.697307 -0.111030
H -2.322343 -3.050997 -1.133853
H -3.543628 -3.060807 0.142525
H -1.835017 -3.218147 0.565596
C -1.801975 -1.090769 2.532649
H -1.296964 -2.065635 2.448161
H -2.725130 -1.261943 3.114929
H -1.157490 -0.442377 3.145566
C -1.980057 2.024871 1.876383
H -1.316852 1.735253 2.705003
H -2.934883 2.346324 2.329786
H -1.541222 2.919882 1.405745
H -2.905932 0.197100 -3.131454
C 2.423636 1.206577 0.013341
C 2.713967 0.264748 -1.015714
C 2.560303 -1.042635 -0.470397
C 2.176398 -0.908585 0.894863
C 2.092965 0.481769 1.194148
C 2.528774 2.698296 -0.099730
H 1.832892 3.215429 0.580274
H 2.317762 3.056362 -1.120649
H 3.541015 3.061623 0.153850
C 3.186062 0.592513 -2.401066
H 2.780356 1.550515 -2.765683
H 2.906470 -0.183294 -3.132493
C 2.838244 -2.334502 -1.179663
H 2.659717 -2.263187 -2.265065
H 2.217598 -3.161145 -0.797278
H 3.889091 -2.654023 -1.058614
C 1.982737 -2.033064 1.867194
H 1.545797 -2.926869 1.392529
H 1.318614 -1.748205 2.696748
H 2.937930 -2.354701 2.319706
C 1.802230 1.079405 2.537291
H 2.725625 1.248534 3.119792
H 1.158535 0.427845 3.147679
H 1.296556 2.054320 2.457510
H 4.286155 0.683511 -2.451452

$BaCp^*_2$
51
Ba 0.000064 0.000500 -0.686633
C -2.733722 1.046705 -0.331280
C -2.934617 -0.254453 -0.870206
C -2.565619 -1.203322 0.124264
C -2.140931 -0.488761 1.279906
C -2.242679 0.903443 0.997049

C -3.045587 2.344130 -1.014263
H -2.395126 3.163943 -0.667262
H -4.083607 2.673948 -0.826673
H -2.937643 2.277970 -2.109860
C -3.491287 -0.571956 -2.225333
H -4.592548 -0.664303 -2.212293
H -3.108403 -1.527446 -2.621419
C -2.674437 -2.693668 0.004335
H -2.518153 -3.043635 -1.029923
H -3.670749 -3.063869 0.307211
H -1.942638 -3.215167 0.642295
C -1.768655 -1.095921 2.597920
H -1.277907 -2.075069 2.482788
H -2.654186 -1.260399 3.238151
H -1.081866 -0.453092 3.169204
C -1.978680 2.020713 1.960465
H -1.268309 1.725241 2.746592
H -2.900994 2.350648 2.471963
H -1.562396 2.913895 1.465287
H -3.257446 0.209824 -2.967120
C 2.565519 1.203178 0.126539
C 2.934587 0.256216 -0.869712
C 2.733863 -1.045979 -0.333216
C 2.242795 -0.905245 0.995380
C 2.140926 0.486426 1.280849
C 2.674349 2.693722 0.009188
H 1.943080 3.214155 0.648613
H 2.517323 3.045530 -1.024340
H 3.670936 3.063290 0.311931
C 3.491074 0.576533 -2.224252
H 3.104657 1.530592 -2.620465
H 3.261040 -0.206001 -2.966384
C 3.045450 -2.342134 -1.018743
H 2.935824 -2.274360 -2.114089
H 2.395926 -3.162795 -0.672031
H 4.083923 -2.671702 -0.833251
C 1.978827 -2.024265 1.956771
H 1.561993 -2.916384 1.460130
H 1.268913 -1.729974 2.743749
H 2.901236 -2.355497 2.467258
C 1.768523 1.090935 2.600039
H 2.653715 1.252012 3.241599
H 1.079837 0.448056 3.168998
H 1.279848 2.071325 2.486817
H 4.591930 0.673457 -2.210246

(BDI*)MgMgCp*(**1**)
141
Mg -0.070064 -0.129363 -0.127184
Mg 2.166715 -1.661465 0.235473
N -2.131063 -0.149399 -0.205158
N 0.038798 1.932152 -0.352070
C -2.886471 0.939768 -0.310998
C -2.333729 2.233817 -0.438552
H -3.083575 3.009109 -0.536442
C -1.012106 2.733856 -0.457348
C -4.446803 0.949480 -0.277514
C -5.141194 -0.414639 -0.202230
H -4.912922 -1.055560 -1.059319
H -6.228828 -0.242322 -0.195162
H -4.888966 -0.971327 0.705867
C -4.975986 1.638786 -1.549879
H -4.669877 2.690065 -1.629604
H -6.076787 1.611193 -1.555580
H -4.621244 1.117858 -2.452207
C -4.883188 1.740524 0.970939
H -4.500387 1.262692 1.885534
H -5.982640 1.760620 1.036062
H -4.523098 2.777954 0.960007
C -0.940945 4.279594 -0.670942
C -1.572957 4.589409 -2.043797
H -1.034579 4.075942 -2.853675
H -1.525851 5.672356 -2.239095
H -2.625150 4.280352 -2.098307
C -1.748348 5.003405 0.422483
H -2.812019 4.730653 0.417106
H -1.682327 6.091873 0.269126
H -1.350750 4.783253 1.423825
C 0.463747 4.894335 -0.670446
H 0.951965 4.833675 0.308534
H 0.372158 5.960370 -0.930562
H 1.134781 4.431545 -1.402578
C -2.549212 -1.502011 -0.127234
C -2.692291 -2.124069 1.135745
C -3.004891 -3.487163 1.179478
H -3.158982 -3.970528 2.145480
C -3.130248 -4.237015 0.014479
H -3.366226 -5.302233 0.068862
C -2.950728 -3.622102 -1.220960
H -3.042064 -4.216881 -2.131429
C -2.667771 -2.256860 -1.318724
C -2.526231 -1.329398 2.415003
H -2.514749 -0.266047 2.128161
C -1.160573 -1.592697 3.080272
H -0.368920 -1.382333 2.337744
H -1.020462 -0.847923 3.879293
C -0.924018 -2.986649 3.639506
H 0.042935 -3.027747 4.162262
H -0.896715 -3.743782 2.842077
H -1.699845 -3.283977 4.362777
C -3.712238 -1.508829 3.374364
H -4.641497 -1.329023 2.808900

H	-3.775833	-2.555188	3.715487
C	-3.669519	-0.583822	4.582918
H	-3.571059	0.470048	4.274276
H	-2.822315	-0.811745	5.247592
H	-4.588321	-0.670551	5.182795
C	-2.465846	-1.598070	-2.671646
H	-2.802375	-0.552789	-2.576527
C	-0.972016	-1.530633	-3.045625
H	-0.892837	-1.089681	-4.053731
H	-0.469173	-0.800008	-2.386005
C	-0.207258	-2.842385	-2.982255
H	0.840399	-2.696167	-3.284023
H	-0.642448	-3.605621	-3.645878
H	-0.200765	-3.247638	-1.959129
C	-3.282625	-2.225182	-3.808567
H	-3.022883	-1.684616	-4.734632
H	-2.959261	-3.265037	-3.983347
C	-4.790987	-2.181251	-3.611064
H	-5.146250	-1.145326	-3.495692
H	-5.100977	-2.738197	-2.714061
H	-5.317734	-2.618168	-4.473246
C	1.397279	2.252331	-0.145772
C	1.835797	2.660670	1.138780
C	3.204812	2.827334	1.359406
H	3.555267	3.148884	2.341174
C	4.135117	2.575888	0.354380
H	5.201652	2.716292	0.544172
C	3.698720	2.123614	-0.885982
H	4.433762	1.904841	-1.660943
C	2.338499	1.936418	-1.157163
C	0.836976	2.845650	2.264884
H	-0.103722	3.196283	1.816582
C	1.257860	3.914071	3.281232
H	2.149457	3.585013	3.839978
H	1.571801	4.813265	2.724487
C	0.162878	4.289274	4.270898
H	0.484827	5.115458	4.922849
H	-0.110827	3.446833	4.924116
H	-0.753401	4.613881	3.751666
C	0.500310	1.490759	2.915001
H	-0.268769	1.646004	3.688584
H	0.003490	0.864011	2.154937
C	1.683194	0.729988	3.488620
H	2.413067	0.484369	2.703077
H	1.357551	-0.216152	3.945169
H	2.208695	1.308059	4.264716
C	1.879992	1.422904	-2.509681
H	0.914031	0.913669	-2.343299
C	1.562916	2.571173	-3.483582
H	1.100427	2.146692	-4.389720
H	0.783444	3.195216	-3.023325
C	2.749959	3.441341	-3.867694
H	2.443859	4.241364	-4.559165
H	3.540196	2.859529	-4.368052
H	3.200750	3.918322	-2.983726
C	2.828029	0.367276	-3.089419
H	3.841816	0.778396	-3.215216
H	2.923640	-0.433330	-2.335112
C	2.373305	-0.230320	-4.412430
H	2.424510	0.498713	-5.235546
H	1.335565	-0.590576	-4.354717
H	3.006243	-1.085312	-4.694741
C	4.473596	-2.097119	-0.131050
C	4.264120	-2.010508	1.278345
C	3.401396	-3.081522	1.662800
C	3.070221	-3.825385	0.488379
C	3.734531	-3.217108	-0.620671
C	5.418335	-1.240523	-0.914000
H	5.192146	-1.255148	-1.989706
H	6.460854	-1.587249	-0.798841
H	5.387337	-0.191684	-0.583902
C	4.923624	-1.022132	2.189998
H	4.921791	-0.005745	1.765720
H	5.977169	-1.291282	2.384260
H	4.419730	-0.971165	3.165707
C	2.985209	-3.416715	3.061424
H	2.832038	-2.515436	3.674792
H	3.745679	-4.031097	3.575173
H	2.046076	-3.988211	3.078116
C	2.223316	-5.059903	0.437462
H	1.434174	-5.047745	1.204641
H	2.820695	-5.974304	0.601596
H	1.724166	-5.171841	-0.536919
C	3.725727	-3.713464	-2.033609
H	2.795804	-4.250833	-2.272075
H	4.559650	-4.411427	-2.226749
H	3.822135	-2.890172	-2.757929

(BDI*)MgCaCp*

141

Mg	0.323616	-0.324923	0.419606
Ca	-2.763358	-0.917181	0.061078
N	2.401794	-0.172566	0.053873
N	0.156763	1.837506	0.098402
C	3.080912	0.911650	-0.285467
C	2.476314	2.190647	-0.371356
H	3.177375	2.978571	-0.617148
C	1.156712	2.651179	-0.218483
C	4.595083	0.943399	-0.668650
C	5.367694	-0.374604	-0.547555
H	5.384091	-0.764745	0.474457
H	6.409820	-0.186136	-0.850017

H	4.971964	-1.161721	-1.196729
C	5.326378	1.959294	0.230306
H	4.974801	2.990343	0.093622
H	6.404012	1.944243	0.004486
H	5.201482	1.701201	1.292868
C	4.689982	1.380367	-2.143491
H	4.164270	0.664119	-2.793061
H	5.745133	1.409635	-2.458770
H	4.253256	2.372845	-2.317833
C	1.020335	4.201771	-0.372513
C	1.834189	4.847956	0.767001
H	1.462049	4.526904	1.750912
H	1.750666	5.945168	0.713592
H	2.898775	4.583792	0.715110
C	1.596708	4.665234	-1.722350
H	2.665986	4.439903	-1.828951
H	1.476007	5.755458	-1.820830
H	1.069439	4.194436	-2.564692
C	-0.406167	4.755605	-0.285225
H	-1.032113	4.445741	-1.129984
H	-0.352579	5.855241	-0.300346
H	-0.923940	4.464869	0.635510
C	2.908290	-1.482939	0.236556
C	2.831315	-2.425491	-0.817135
C	3.258317	-3.735602	-0.572974
H	3.248153	-4.461925	-1.387095
C	3.702810	-4.129310	0.684765
H	4.024033	-5.159168	0.856927
C	3.730795	-3.204245	1.724203
H	4.069384	-3.521550	2.712036
C	3.345493	-1.875856	1.525163
C	2.334906	-2.025520	-2.193522
H	2.266474	-0.925761	-2.197389
C	0.903500	-2.527458	-2.471867
H	0.239477	-2.111577	-1.689481
H	0.566627	-2.077951	-3.419840
C	0.690022	-4.032005	-2.519980
H	-0.347930	-4.259492	-2.809221
H	0.861921	-4.495178	-1.537314
H	1.347198	-4.530013	-3.251102
C	3.331715	-2.405866	-3.299933
H	4.332707	-2.055789	-2.998542
H	3.416984	-3.502345	-3.377749
C	2.987228	-1.826936	-4.665477
H	2.864638	-0.732292	-4.613807
H	2.051017	-2.243720	-5.067015
H	3.780360	-2.038312	-5.399000
C	3.370136	-0.877847	2.670243
H	3.577621	0.112615	2.233422
C	1.988455	-0.764803	3.345725
H	2.076055	-0.056442	4.187541

H	1.283307	-0.281507	2.643023
C	1.371999	-2.072455	3.816204
H	0.408727	-1.889662	4.315591
H	2.022184	-2.602995	4.529260
H	1.175858	-2.745178	2.967515
C	4.463197	-1.139785	3.714565
H	4.353473	-0.373708	4.501026
H	4.282074	-2.101026	4.224017
C	5.886955	-1.103966	3.178178
H	6.113820	-0.130458	2.716287
H	6.053995	-1.878420	2.414501
H	6.619289	-1.267209	3.983773
C	-1.216942	2.044616	0.006321
C	-1.853894	2.083388	-1.269640
C	-3.253980	2.093406	-1.330450
H	-3.747786	2.129571	-2.303016
C	-4.037264	2.059953	-0.174068
H	-5.125951	2.080344	-0.245008
C	-3.411766	1.994606	1.072349
H	-4.026374	1.948775	1.972223
C	-2.012762	1.962171	1.189866
C	-1.025038	2.025138	-2.538817
H	-0.036992	2.439799	-2.297551
C	-1.595240	2.876748	-3.678631
H	-2.582664	2.497784	-3.989954
H	-1.777358	3.894230	-3.292643
C	-0.686401	2.948486	-4.898228
H	-1.087639	3.643116	-5.651550
H	-0.572859	1.967552	-5.384292
H	0.322642	3.298138	-4.626669
C	-0.759427	0.561242	-2.935426
H	0.037425	0.529198	-3.695124
H	-0.337862	0.047206	-2.051200
C	-1.964932	-0.224477	-3.422695
H	-2.799889	-0.200161	-2.700982
H	-1.706279	-1.281006	-3.582961
H	-2.367625	0.166811	-4.369489
C	-1.354307	1.851354	2.551484
H	-0.393118	1.335554	2.380627
C	-0.987959	3.230229	3.127360
H	-0.402506	3.077924	4.048400
H	-0.297977	3.715627	2.422295
C	-2.164836	4.152328	3.408448
H	-1.820875	5.117721	3.810313
H	-2.859040	3.719420	4.145904
H	-2.741873	4.360314	2.493751
C	-2.151099	0.970944	3.521133
H	-3.141107	1.407727	3.731507
H	-2.341088	0.007944	3.011780
C	-1.438038	0.696510	4.836474
H	-1.346038	1.602181	5.455190

H	-0.423714	0.307276	4.661457
H	-1.984226	-0.051785	5.430264
C	-5.170309	-1.588280	0.795074
C	-5.154712	-1.707318	-0.625169
C	-4.262784	-2.765555	-0.966980
C	-3.726311	-3.298307	0.239598
C	-4.285092	-2.570079	1.327860
C	-6.022446	-0.653371	1.598414
H	-5.514847	-0.313575	2.515470
H	-6.965622	-1.130865	1.918399
H	-6.300130	0.244826	1.026681
C	-5.987673	-0.919365	-1.590267
H	-6.299420	0.047281	-1.167497
H	-6.911136	-1.456708	-1.870569
H	-5.450740	-0.706137	-2.529017
C	-3.989320	-3.281769	-2.347146
H	-4.216377	-2.531535	-3.119307
H	-4.600149	-4.172551	-2.578134
H	-2.936354	-3.580093	-2.477560
C	-2.774804	-4.450171	0.349330
H	-2.136221	-4.540915	-0.542247
H	-3.308707	-5.410835	0.464369
H	-2.103232	-4.345351	1.215107
C	-4.017504	-2.828471	2.779688
H	-2.964053	-3.094429	2.963150
H	-4.630420	-3.661800	3.167263
H	-4.246481	-1.949518	3.401425

(BDI*)MgSrCp*

141

Mg	-0.529227	-0.227715	-0.578589
Sr	2.770185	-0.792633	-0.390158
N	-2.577523	-0.320196	-0.015086
N	-0.478143	1.840580	0.168771
C	-3.292177	0.621403	0.579660
C	-2.768115	1.906510	0.864854
H	-3.496738	2.574384	1.306653
C	-1.502307	2.495812	0.704355
C	-4.764884	0.457765	1.076226
C	-5.456078	-0.873994	0.765009
H	-5.539729	-1.069268	-0.308147
H	-6.476422	-0.831643	1.177488
H	-4.951163	-1.731593	1.219832
C	-5.640259	1.559581	0.447960
H	-5.347867	2.572409	0.754707
H	-6.689850	1.413277	0.747063
H	-5.594429	1.516367	-0.650739
C	-4.755324	0.604873	2.610364
H	-4.130170	-0.176032	3.069506
H	-5.777966	0.492071	3.004157
H	-4.368044	1.578926	2.937979
C	-1.468048	4.002077	1.129833
C	-2.406551	4.763814	0.171623
H	-2.081782	4.652321	-0.873168
H	-2.403091	5.838092	0.415918
H	-3.441396	4.401954	0.235209
C	-1.982329	4.174505	2.570640
H	-3.025870	3.856211	2.693260
H	-1.924952	5.236120	2.857664
H	-1.371636	3.603213	3.284781
C	-0.097591	4.686611	1.067058
H	0.605819	4.292940	1.809909
H	-0.236628	5.757847	1.280217
H	0.379436	4.606722	0.084310
C	-3.017603	-1.602482	-0.424651
C	-2.796452	-2.727300	0.406072
C	-3.139657	-3.993625	-0.080038
H	-3.010499	-4.866455	0.562012
C	-3.651224	-4.162028	-1.362622
H	-3.904413	-5.160378	-1.726801
C	-3.839704	-3.050724	-2.179013
H	-4.239710	-3.189339	-3.185002
C	-3.538598	-1.761217	-1.732147
C	-2.245001	-2.565979	1.810373
H	-2.292471	-1.490542	2.045288
C	-0.753208	-2.940424	1.905086
H	-0.199632	-2.298000	1.192822
H	-0.393867	-2.657696	2.907789
C	-0.382202	-4.386174	1.618449
H	0.696739	-4.541715	1.776926
H	-0.599764	-4.656947	0.574672
H	-0.912321	-5.096982	2.272564
C	-3.111552	-3.282941	2.857941
H	-4.163669	-3.000118	2.689109
H	-3.074220	-4.373946	2.704203
C	-2.729693	-2.962773	4.296797
H	-2.726612	-1.874633	4.475096
H	-1.727982	-3.340451	4.552527
H	-3.440019	-3.413721	5.006616
C	-3.751906	-0.554045	-2.630376
H	-3.990335	0.298804	-1.974285
C	-2.459364	-0.167956	-3.376326
H	-2.679202	0.704711	-4.015373
H	-1.717717	0.199790	-2.641638
C	-1.816175	-1.275969	-4.193898
H	-0.914807	-0.909134	-4.707045
H	-2.498964	-1.672045	-4.961699
H	-1.507048	-2.111276	-3.547859
C	-4.921249	-0.697494	-3.613513
H	-4.948385	0.220870	-4.224611
H	-4.720329	-1.511859	-4.329394
C	-6.283329	-0.908172	-2.968149

H	-6.537219	-0.076378	-2.292854
H	-6.314274	-1.835214	-2.376070
H	-7.077161	-0.971924	-3.728234
C	0.878786	2.156083	0.210831
C	1.606402	2.033128	1.432436
C	2.999660	2.182598	1.411678
H	3.562361	2.103362	2.343517
C	3.689530	2.436584	0.222662
H	4.773426	2.563226	0.229696
C	2.976951	2.527124	-0.974574
H	3.519941	2.715855	-1.902257
C	1.581594	2.373542	-1.012195
C	0.880219	1.674181	2.716073
H	-0.143111	2.062314	2.625628
C	1.488540	2.319378	3.965624
H	2.512145	1.946183	4.135195
H	1.594877	3.401911	3.780608
C	0.665109	2.095345	5.226702
H	1.088187	2.646378	6.080120
H	0.626993	1.032215	5.509276
H	-0.374260	2.435648	5.092702
C	0.720728	0.147198	2.829438
H	-0.035813	-0.087161	3.594715
H	0.287685	-0.213805	1.877702
C	1.996010	-0.627656	3.115643
H	2.819292	-0.347457	2.435483
H	1.833130	-1.710788	3.014381
H	2.377985	-0.447668	4.132164
C	0.826287	2.469541	-2.324293
H	-0.090387	1.868844	-2.192360
C	0.341746	3.903810	-2.602768
H	-0.310053	3.883293	-3.490982
H	-0.311364	4.205045	-1.771502
C	1.439039	4.940564	-2.790731
H	1.009973	5.934921	-2.988100
H	2.096925	4.693760	-3.639010
H	2.072771	5.025394	-1.894136
C	1.587535	1.840711	-3.496940
H	2.545982	2.356517	-3.671959
H	1.839331	0.804708	-3.202324
C	0.797197	1.802449	-4.796638
H	0.639225	2.807623	-5.216301
H	-0.192425	1.345754	-4.642741
H	1.323038	1.208390	-5.559234
C	5.506506	-1.008825	-0.839459
C	5.354591	-1.310590	0.543282
C	4.650453	-2.545078	0.647297
C	4.370456	-3.006971	-0.670826
C	4.897413	-2.055820	-1.589747
C	6.244192	0.159139	-1.421871
H	5.705534	0.623677	-2.265682
H	7.235879	-0.133600	-1.810187
H	6.417458	0.945841	-0.673157
C	5.911051	-0.515536	1.686062
H	5.942869	0.562477	1.464107
H	6.943654	-0.818112	1.935856
H	5.316761	-0.639835	2.604894
C	4.342869	-3.293964	1.908936
H	4.382916	-2.642945	2.794812
H	5.062649	-4.113598	2.083465
H	3.342938	-3.759056	1.887815
C	3.703606	-4.304938	-1.013948
H	2.886584	-4.551890	-0.316508
H	4.413530	-5.151095	-0.980922
H	3.271975	-4.290929	-2.026230
C	4.881483	-2.159562	-3.085345
H	4.017162	-2.735369	-3.451984
H	5.785365	-2.662819	-3.473455
H	4.844329	-1.169539	-3.568567

(BDI*)MgBaCp*

141

Mg	-0.735697	-0.141297	-0.748916
Ba	2.771461	-0.613044	-0.497268
N	-2.727501	-0.423283	0.003397
N	-0.746843	1.845683	0.239220
C	-3.443598	0.408497	0.738889
C	-2.985683	1.706631	1.080846
H	-3.723065	2.289887	1.617505
C	-1.776155	2.390098	0.882728
C	-4.843235	0.093494	1.358406
C	-5.464640	-1.261990	1.006201
H	-5.646971	-1.380712	-0.065933
H	-6.435375	-1.335770	1.521338
H	-4.851513	-2.107044	1.333611
C	-5.855275	1.167287	0.913868
H	-5.611122	2.172045	1.283248
H	-6.856692	0.910661	1.293065
H	-5.914384	1.219274	-0.183701
C	-4.684987	0.124643	2.891221
H	-3.963945	-0.639197	3.220356
H	-5.651141	-0.094933	3.372921
H	-4.332015	1.097836	3.257850
C	-1.815402	3.869657	1.395820
C	-2.834828	4.616092	0.511188
H	-2.539710	4.578771	-0.547799
H	-2.893215	5.674628	0.811398
H	-3.840207	4.180860	0.588660
C	-2.281316	3.936105	2.861383
H	-3.301220	3.556349	3.006061
H	-2.268880	4.982419	3.204614
H	-1.612830	3.362197	3.519650

C	-0.493916	4.643659	1.323418
H	0.259427	4.258908	2.020837
H	-0.692222	5.691692	1.596782
H	-0.048754	4.644535	0.322825
C	-3.122622	-1.689724	-0.489194
C	-2.744388	-2.869856	0.195288
C	-3.047897	-4.105768	-0.386199
H	-2.795036	-5.024161	0.146187
C	-3.673204	-4.188357	-1.625885
H	-3.892879	-5.163252	-2.067218
C	-4.019320	-3.020761	-2.299675
H	-4.509952	-3.091102	-3.272137
C	-3.762873	-1.761277	-1.751129
C	-2.075945	-2.806837	1.556396
H	-2.179463	-1.767937	1.909189
C	-0.559020	-3.066949	1.485496
H	-0.122389	-2.283100	0.834657
H	-0.136096	-2.903268	2.490010
C	-0.110507	-4.417064	0.951555
H	0.985302	-4.511362	1.020404
H	-0.382418	-4.535992	-0.107959
H	-0.542652	-5.261117	1.512540
C	-2.785654	-3.696377	2.590139
H	-3.869935	-3.508883	2.523202
H	-2.656780	-4.760460	2.331922
C	-2.324294	-3.464838	4.022762
H	-2.419314	-2.403014	4.304367
H	-1.271817	-3.751268	4.170526
H	-2.925135	-4.051880	4.734187
C	-4.149762	-0.493880	-2.495558
H	-4.369048	0.273072	-1.735212
C	-2.974620	0.057205	-3.326823
H	-3.310775	0.981569	-3.827697
H	-2.170738	0.381663	-2.638179
C	-2.375752	-0.906028	-4.338676
H	-1.561784	-0.423921	-4.900438
H	-3.121903	-1.255501	-5.069266
H	-1.947788	-1.787335	-3.837552
C	-5.408656	-0.630958	-3.362332
H	-5.562986	0.337099	-3.869028
H	-5.234949	-1.355047	-4.175622
C	-6.675062	-1.007165	-2.606793
H	-6.909355	-0.266651	-1.826452
H	-6.579009	-1.986539	-2.114249
H	-7.541000	-1.059036	-3.284667
C	0.588229	2.234778	0.275540
C	1.347940	2.087575	1.476912
C	2.722302	2.363191	1.454066
H	3.302453	2.279179	2.375133
C	3.369073	2.750883	0.275633
H	4.437923	2.974409	0.280067

C	2.630565	2.853183	-0.906058
H	3.138785	3.148771	-1.826058
C	1.250652	2.590562	-0.938206
C	0.676028	1.557767	2.730461
H	-0.377296	1.864379	2.687904
C	1.248286	2.125558	4.032007
H	2.301590	1.824276	4.158101
H	1.262155	3.226073	3.954481
C	0.461612	1.710209	5.267932
H	0.853089	2.200010	6.172226
H	0.506675	0.623473	5.437651
H	-0.602120	1.981788	5.175154
C	0.648633	0.018262	2.688306
H	-0.111679	-0.360112	3.389339
H	0.277188	-0.277804	1.687007
C	1.979300	-0.665361	2.963485
H	2.815138	-0.207014	2.406366
H	1.940635	-1.735775	2.708677
H	2.270402	-0.601659	4.022680
C	0.464805	2.744797	-2.226555
H	-0.426742	2.103306	-2.117861
C	-0.076822	4.175246	-2.405329
H	-0.739165	4.184312	-3.285942
H	-0.731388	4.397266	-1.550121
C	0.973178	5.267899	-2.539963
H	0.499369	6.251775	-2.679526
H	1.634661	5.097960	-3.404243
H	1.608979	5.331400	-1.643211
C	1.223843	2.217703	-3.449231
H	2.149963	2.790927	-3.621878
H	1.530385	1.181307	-3.213126
C	0.396458	2.198375	-4.725693
H	0.154846	3.211660	-5.081061
H	-0.552396	1.662720	-4.566875
H	0.935336	1.686317	-5.537186
C	5.721525	-0.700053	-0.709599
C	5.479157	-1.109052	0.630278
C	4.854493	-2.388079	0.592509
C	4.714718	-2.770175	-0.772503
C	5.250393	-1.725848	-1.577343
C	6.410189	0.561679	-1.133654
H	5.973081	0.994867	-2.049438
H	7.481161	0.397066	-1.350162
H	6.362718	1.334506	-0.351371
C	5.865261	-0.340864	1.858358
H	5.647111	0.736443	1.764821
H	6.944870	-0.423157	2.076567
H	5.334547	-0.702564	2.751935
C	4.487223	-3.242686	1.767968
H	4.445343	-2.662851	2.701860
H	5.218878	-4.054293	1.929756

H	3.506866	-3.734344	1.643874
C	4.173372	-4.080981	-1.257933
H	3.347185	-4.451462	-0.628958
H	4.945415	-4.871812	-1.255495
H	3.793193	-4.016501	-2.289431
C	5.364014	-1.728270	-3.071977
H	4.565259	-2.318159	-3.550103
H	6.320670	-2.162957	-3.413678
H	5.318745	-0.710480	-3.493499

NaCp*
26

Na	0.000501	0.000507	1.975676
C	-0.999167	0.688089	-0.208983
C	0.345706	1.162782	-0.209216
C	1.212757	0.030418	-0.209211
C	0.403733	-1.144123	-0.208991
C	-0.963325	-0.737650	-0.208806
C	-2.230437	1.536085	-0.330334
H	-3.110449	1.063901	0.136610
H	-2.503364	1.725680	-1.385431
H	-2.104280	2.525700	0.138923
C	0.771788	2.595813	-0.330556
H	0.867777	2.913988	-1.385647
H	1.751981	2.781552	0.138677
C	2.707305	0.068073	-0.330539
H	3.142000	0.966314	0.138126
H	3.039542	0.077101	-1.385624
H	3.186588	-0.807772	0.137010
C	0.901407	-2.553881	-0.330258
H	1.889565	-2.690003	0.139290
H	1.013605	-2.866761	-1.385327
H	0.216029	-3.280387	0.136489
C	-2.150456	-1.646399	-0.330180
H	-1.972667	-2.630058	0.134791
H	-2.416457	-1.845473	-1.385293
H	-3.052067	-1.221488	0.141022
H	0.050818	3.286884	0.136392

$(NaCp^*)_2$
52

Na	-4.234990	-0.001746	0.156255
C	-1.969826	-0.807337	0.886762
C	-2.076535	-1.115276	-0.505743
C	-2.132930	0.118354	-1.228032
C	-2.060439	1.188031	-0.280910
C	-1.960075	0.615416	1.025546
C	-1.757423	-1.799116	1.993224
H	-2.163235	-1.441215	2.952339
H	-0.685589	-2.002583	2.164410
H	-2.229964	-2.769417	1.774641
C	-2.007885	-2.488381	-1.108979
H	-0.967463	-2.811439	-1.294216
H	-2.527485	-2.539728	-2.078446
C	-2.145280	0.264879	-2.722290
H	-2.650300	-0.580345	-3.215493
H	-1.126708	0.309824	-3.148803
H	-2.655371	1.186817	-3.042847
C	-1.968734	2.650439	-0.607727
H	-2.484307	2.896080	-1.549171
H	-0.922578	2.985830	-0.726662
H	-2.406587	3.279344	0.183321
C	-1.736045	1.372216	2.302210
H	-2.201480	2.369923	2.278121
H	-0.662122	1.531013	2.503962
H	-2.141599	0.837953	3.175537
H	-2.455727	-3.251264	-0.452890
Na	0.283642	-0.000250	-0.186973
C	2.413230	-0.801902	0.929521
C	2.537642	-1.110752	-0.454824
C	2.602344	0.116287	-1.175258
C	2.516802	1.182940	-0.235304
C	2.400624	0.615050	1.065103
C	2.384277	-1.793963	2.052916
H	1.828051	-1.414446	2.926573
H	3.395904	-2.053950	2.419264
H	1.910755	-2.744884	1.755324
C	2.661392	-2.484985	-1.041393
H	3.710204	-2.835160	-1.090714
H	2.270826	-2.532843	-2.071887
C	2.812587	0.259991	-2.652861
H	2.380686	-0.583463	-3.217500
H	3.883707	0.301411	-2.928451
H	2.355651	1.182365	-3.049568
C	2.614795	2.645444	-0.550526
H	2.220161	2.881204	-1.553260
H	3.657197	3.017179	-0.532584
H	2.054127	3.263577	0.171034
C	2.358251	1.375275	2.356260
H	1.871923	2.358994	2.243397
H	3.366347	1.574480	2.767661
H	1.807509	0.829428	3.140759
H	2.111677	-3.238407	-0.452110

$(NaCp^*)_3$
78

Na	-6.364520	0.170908	-0.726629
C	-4.001130	-0.592736	-1.131374
C	-3.951643	0.830088	-1.003387
C	-4.305014	1.165837	0.341271
C	-4.571798	-0.050679	1.046020
C	-4.384070	-1.137774	0.134481

C	-3.600624	-1.387857	-2.339463
H	-4.156241	-2.335755	-2.413345
H	-2.529054	-1.651342	-2.323958
H	-3.767990	-0.831039	-3.274014
C	-3.484901	1.798260	-2.050786
H	-2.412610	2.036638	-1.942920
H	-4.022827	2.758348	-2.000431
C	-4.285891	2.546678	0.930694
H	-4.582606	3.312985	0.197402
H	-3.281727	2.838114	1.288630
H	-4.961949	2.636017	1.794965
C	-4.904721	-0.169079	2.505749
H	-5.457534	0.708905	2.874626
H	-4.005415	-0.256673	3.142321
H	-5.518960	-1.057987	2.718095
C	-4.465362	-2.599549	0.468089
H	-5.123689	-2.788745	1.329704
H	-3.479964	-3.027931	0.726369
H	-4.845211	-3.197190	-0.375741
H	-3.615581	1.397293	-3.067014
Na	-2.011818	-0.082132	0.476932
C	0.161732	-0.206900	-0.868206
C	0.225427	0.999384	-0.110598
C	0.288014	0.652274	1.271801
C	0.262201	-0.772751	1.370531
C	0.182462	-1.303783	0.045608
C	0.157824	-0.300099	-2.365896
H	-0.074310	-1.321021	-2.703461
H	1.137494	-0.039235	-2.802416
H	-0.583724	0.372510	-2.828550
C	0.307509	2.389257	-0.669291
H	1.330110	2.801842	-0.615133
H	-0.345874	3.097896	-0.132507
C	0.447940	1.622482	2.405168
H	-0.080329	2.569905	2.210511
H	1.505095	1.888632	2.587860
H	0.059043	1.216997	3.352472
C	0.374910	-1.571453	2.636414
H	-0.035953	-1.026806	3.501274
H	1.420697	-1.820422	2.893858
H	-0.163799	-2.530236	2.567790
C	0.185112	-2.759734	-0.319588
H	-0.215243	-3.382881	0.495430
H	1.197625	-3.143919	-0.539965
H	-0.426339	-2.962921	-1.213475
H	0.016674	2.413091	-1.730156
Na	2.542888	-0.163937	0.208635
C	4.481511	0.198816	-1.400590
C	4.544950	1.264427	-0.459680
C	4.853411	0.709439	0.814074
C	4.982068	-0.700305	0.660327
C	4.751548	-1.015784	-0.709135
C	4.238202	0.337454	-2.873044
H	3.767145	-0.563831	-3.300105
H	5.169493	0.502778	-3.448539
H	3.575961	1.188758	-3.105600
C	4.388187	2.722696	-0.768189
H	5.352393	3.219366	-0.990560
H	3.940698	3.280629	0.072175
C	5.081109	1.483313	2.077644
H	4.484851	2.411057	2.107425
H	6.137137	1.789406	2.207808
H	4.815106	0.900110	2.975434
C	5.376264	-1.669696	1.733942
H	5.031667	-1.346701	2.730911
H	6.473110	-1.798325	1.811574
H	4.958542	-2.675523	1.558662
C	4.849187	-2.377643	-1.328349
H	4.593784	-3.176811	-0.612227
H	5.866426	-2.607316	-1.700066
H	4.171504	-2.490395	-2.191720
H	3.743831	2.890992	-1.647148

$(NaCp^{*})_4$

104

Na	-8.645915	0.878717	-0.003502
C	-6.245217	1.355781	0.606144
C	-6.563158	0.089553	1.190549
C	-6.794964	-0.841517	0.129003
C	-6.619560	-0.149818	-1.111343
C	-6.279858	1.207934	-0.815773
C	-5.830715	2.591309	1.351438
H	-6.102919	3.510703	0.810190
H	-4.738888	2.636541	1.510065
H	-6.292715	2.645687	2.349249
C	-6.546575	-0.231501	2.657301
H	-5.545533	-0.539192	3.010008
H	-7.230273	-1.057675	2.906633
C	-7.074673	-2.308329	0.288736
H	-7.628641	-2.524108	1.215526
H	-6.151203	-2.913984	0.333218
H	-7.665377	-2.709177	-0.549538
C	-6.675259	-0.764543	-2.480262
H	-7.366239	-1.620771	-2.520222
H	-5.691980	-1.143259	-2.813492
H	-7.000816	-0.041539	-3.244250
C	-5.906524	2.261252	-1.818267
H	-6.416687	2.112696	-2.782494
H	-4.823731	2.265780	-2.035526
H	-6.155042	3.274002	-1.464929
H	-6.836497	0.634329	3.272631
Na	-4.262657	-0.225093	0.003026

C	-1.933544	0.646950	0.597067
C	-2.044634	-0.644097	1.196874
C	-2.127426	-1.612089	0.148838
C	-2.067443	-0.916790	-1.098307
C	-1.947653	0.478773	-0.820029
C	-1.760310	1.945164	1.328507
H	-2.180263	2.791899	0.763134
H	-0.699427	2.189418	1.513974
H	-2.253848	1.929070	2.313041
C	-2.021694	-0.932729	2.669449
H	-0.997125	-1.045156	3.068369
H	-2.554667	-1.865556	2.911536
C	-2.214364	-3.100233	0.325505
H	-2.725328	-3.371452	1.262827
H	-1.223600	-3.589292	0.359416
H	-2.766888	-3.578771	-0.498721
C	-2.072736	-1.543650	-2.461952
H	-2.618172	-2.500442	-2.470100
H	-1.056415	-1.760299	-2.838617
H	-2.546508	-0.889529	-3.211290
C	-1.793343	1.569495	-1.838446
H	-2.318343	1.331094	-2.777021
H	-0.738066	1.753161	-2.107725
H	-2.192052	2.528677	-1.472607
H	-2.492974	-0.125126	3.252175
Na	0.270433	-0.595296	0.005529
C	2.412673	0.685027	0.585392
C	2.545429	-0.607227	1.175950
C	2.632481	-1.567371	0.122192
C	2.552752	-0.866043	-1.119729
C	2.417311	0.525547	-0.832037
C	2.359878	1.988595	1.325110
H	1.804149	2.754837	0.761866
H	3.364442	2.405716	1.516922
H	1.871046	1.882840	2.306789
C	2.647353	-0.894557	2.645046
H	3.684712	-0.828541	3.020873
H	2.289185	-1.907136	2.889698
C	2.835306	-3.044723	0.291034
H	2.370675	-3.418533	1.217541
H	3.902803	-3.327658	0.341743
H	2.401493	-3.615596	-0.545406
C	2.654675	-1.473770	-2.487912
H	2.307500	-2.519081	-2.497268
H	3.689753	-1.483188	-2.875865
H	2.051271	-0.922170	-3.226924
C	2.366681	1.632554	-1.842844
H	1.901395	1.304235	-2.785937
H	3.370230	2.014920	-2.102569
H	1.789365	2.495442	-1.474634
H	2.054107	-0.183906	3.242774
Na	4.819112	-0.121051	-0.031147
C	6.713179	1.443555	0.654693
C	7.036968	0.163879	1.186675
C	7.288330	-0.715444	0.095533
C	7.118697	0.021597	-1.110956
C	6.763024	1.355699	-0.764727
C	6.435480	2.682545	1.450945
H	5.775073	3.381325	0.910437
H	7.354176	3.250273	1.695514
H	5.946787	2.456132	2.413819
C	7.159573	-0.178526	2.640924
H	8.180321	-0.012214	3.036505
H	6.919489	-1.236874	2.839118
C	7.732525	-2.143460	0.200783
H	7.335010	-2.635451	1.104686
H	8.833833	-2.246675	0.250265
H	7.404067	-2.744667	-0.663876
C	7.347006	-0.496012	-2.499252
H	7.133837	-1.575393	-2.578801
H	8.391208	-0.359606	-2.841358
H	6.709261	0.013451	-3.241535
C	6.542988	2.485362	-1.724974
H	6.149469	2.133673	-2.693659
H	7.471960	3.043619	-1.951858
H	5.824186	3.225904	-1.335173
H	6.482768	0.427708	3.266619

$(NaCp^*)_5$

130

Na	10.837634	-1.223357	-0.140031
C	8.395052	-1.748563	0.225909
C	8.734737	-0.735194	1.176653
C	9.044713	0.461375	0.455755
C	8.895958	0.186665	-0.940679
C	8.494479	-1.179072	-1.082140
C	7.907100	-3.131028	0.549439
H	8.155381	-3.852392	-0.244334
H	6.810185	-3.169583	0.671753
H	8.337671	-3.511612	1.488540
C	8.670583	-0.874229	2.670440
H	7.666725	-0.646660	3.072570
H	9.369490	-0.192084	3.178602
C	9.372229	1.795909	1.061601
H	9.889312	1.694069	2.028279
H	8.471522	2.406200	1.256053
H	10.017098	2.400033	0.404729
C	9.031004	1.183605	-2.055472
H	9.749709	1.980861	-1.810792
H	8.075672	1.687763	-2.288978
H	9.366677	0.712515	-2.992380
C	8.127826	-1.860100	-2.368927

H	8.693667	-1.459248	-3.224094
H	7.058777	-1.736994	-2.617752
H	8.312960	-2.944630	-2.329071
H	8.908944	-1.897305	3.000412
Na	6.501741	0.032909	0.075508
C	4.100126	-0.813234	0.321772
C	4.259161	0.231019	1.282669
C	4.443811	1.458335	0.574381
C	4.398468	1.170661	-0.824757
C	4.185859	-0.233141	-0.979653
C	3.827849	-2.256854	0.626112
H	4.226135	-2.921341	-0.156644
H	2.748962	-2.479474	0.705058
H	4.280300	-2.565840	1.581518
C	4.189767	0.072511	2.773424
H	3.160031	0.155783	3.166163
H	4.784382	0.839244	3.294446
C	4.610644	2.816604	1.191327
H	5.110237	2.762136	2.171490
H	3.648987	3.334335	1.362199
H	5.211944	3.484208	0.554156
C	4.507206	2.172433	-1.937111
H	5.124088	3.038161	-1.649003
H	3.527315	2.578076	-2.248674
H	4.960540	1.733110	-2.839832
C	4.019899	-0.961721	-2.280681
H	4.585089	-0.480372	-3.094235
H	2.967514	-1.005579	-2.613875
H	4.366780	-2.004736	-2.211783
H	4.565766	-0.910670	3.098066
Na	2.007358	0.700275	0.077864
C	-0.254880	-0.490372	0.312342
C	-0.291612	0.618052	1.210694
C	-0.272012	1.816909	0.434267
C	-0.223050	1.447441	-0.945138
C	-0.212646	0.021283	-1.019112
C	-0.311458	-1.938567	0.698731
H	0.182931	-2.578456	-0.048738
H	-1.345952	-2.312587	0.796489
H	0.181219	-2.123330	1.666488
C	-0.390667	0.535625	2.705657
H	-1.435475	0.498488	3.064248
H	0.077002	1.404515	3.195191
C	-0.338104	3.215720	0.974456
H	0.154730	3.298588	1.956330
H	-1.373944	3.575089	1.116418
H	0.151585	3.937044	0.301440
C	-0.223602	2.388290	-2.114479
H	0.216052	3.363574	-1.853042
H	-1.238368	2.596484	-2.500524
H	0.353732	1.986922	-2.962778

C	-0.211873	-0.795930	-2.277286
H	0.293388	-0.271680	-3.103713
H	-1.230471	-1.034508	-2.633361
H	0.302636	-1.759786	-2.137627
H	0.104297	-0.366942	3.097752
Na	-2.546682	0.661549	-0.077963
C	-4.580022	-0.879190	0.204047
C	-4.772574	0.207191	1.108374
C	-4.964713	1.393924	0.338521
C	-4.889852	1.039784	-1.043232
C	-4.651798	-0.365724	-1.125172
C	-4.407545	-2.319738	0.583917
H	-3.810054	-2.869880	-0.160285
H	-5.371352	-2.852536	0.671236
H	-3.900890	-2.427220	1.556128
C	-4.833617	0.105715	2.603720
H	-5.851393	-0.115825	2.972188
H	-4.519702	1.043076	3.089817
C	-5.259378	2.759552	0.886181
H	-4.766188	2.926384	1.857335
H	-6.339076	2.929801	1.051451
H	-4.917700	3.555239	0.205266
C	-5.082256	1.969372	-2.205439
H	-4.771810	2.997924	-1.961780
H	-6.135350	2.030820	-2.536461
H	-4.498187	1.651691	-3.084154
C	-4.552491	-1.172446	-2.386062
H	-4.155747	-0.575573	-3.222722
H	-5.529598	-1.568889	-2.717791
H	-3.888713	-2.042921	-2.261685
H	-4.180821	-0.695555	2.985485
Na	-7.055841	-0.118393	-0.119017
C	-8.858650	-1.857851	0.363971
C	-9.073746	-0.795777	1.286217
C	-9.468129	0.355309	0.547794
C	-9.496854	0.004230	-0.831805
C	-9.119478	-1.363901	-0.945000
C	-8.485476	-3.266304	0.715061
H	-7.899066	-3.750879	-0.083918
H	-9.366920	-3.913635	0.889158
H	-7.878923	-3.315772	1.635081
C	-8.964783	-0.890051	2.778101
H	-9.914410	-1.193502	3.260151
H	-8.680832	0.073640	3.233709
C	-9.854017	1.682785	1.127598
H	-9.307182	1.901806	2.060442
H	-10.931068	1.745463	1.376975
H	-9.648542	2.513752	0.431780
C	-9.924692	0.895522	-1.958762
H	-9.700159	1.956360	-1.755846
H	-11.012394	0.841087	-2.158969

H -9.422492 0.635822 -2.906137
C -9.071443 -2.162883 -2.212447
H -8.820348 -1.538068 -3.086231
H -10.035997 -2.653178 -2.447984
H -8.318406 -2.967833 -2.165615
H -8.208528 -1.629509 3.091572

$(NaCp^*)_6$
156
Na 8.697214 -0.279895 0.143396
C 6.256100 -0.808510 0.687695
C 6.547714 0.439269 1.318343
C 6.783746 1.407365 0.293760
C 6.637093 0.756073 -0.969816
C 6.310656 -0.612464 -0.725218
C 5.899118 -2.088157 1.385103
H 6.189750 -2.969738 0.792507
H 4.814982 -2.183414 1.573881
H 6.394924 -2.173220 2.364975
C 6.554296 0.697804 2.796823
H 5.562222 0.990250 3.186762
H 7.245936 1.511131 3.067406
C 7.092870 2.860622 0.507985
H 7.641796 3.027251 1.448267
H 6.187177 3.492176 0.562097
H 7.708448 3.272372 -0.307296
C 6.759984 1.404557 -2.317649
H 7.442593 2.268368 -2.293946
H 5.795171 1.780051 -2.704776
H 7.145204 0.703700 -3.075127
C 6.015318 -1.648997 -1.768881
H 6.532281 -1.432224 -2.716561
H 4.939202 -1.718022 -2.008336
H 6.326634 -2.655444 -1.447181
H 6.858857 -0.194517 3.366236
Na 4.275111 0.761704 0.106591
C 1.926027 -0.137594 0.634742
C 2.005429 1.151517 1.243117
C 2.102248 2.125607 0.202375
C 2.082253 1.436118 -1.049029
C 1.973084 0.037944 -0.780584
C 1.769124 -1.442756 1.357353
H 2.209072 -2.277802 0.790084
H 0.711829 -1.708396 1.535355
H 2.257198 -1.423984 2.344563
C 1.952656 1.431092 2.716583
H 0.920117 1.518973 3.101367
H 2.462100 2.373504 2.971281
C 2.175128 3.613247 0.388730
H 2.671518 3.882405 1.334432
H 1.180889 4.096288 0.411283
H 2.737038 4.099470 -0.424404
C 2.127114 2.069258 -2.408962
H 2.668467 3.028243 -2.394928
H 1.122951 2.283425 -2.818819
H 2.629725 1.421163 -3.144489
C 1.872002 -1.050643 -1.807794
H 2.421612 -0.796723 -2.727903
H 0.829931 -1.255982 -2.112068
H 2.280940 -2.002329 -1.433996
H 2.433588 0.631054 3.301857
Na -0.267089 1.081924 -0.006232
C -2.434610 -0.226372 0.449061
C -2.566743 1.017466 1.136605
C -2.637089 2.056939 0.159228
C -2.548084 1.453295 -1.132620
C -2.423211 0.042487 -0.952116
C -2.374106 -1.582976 1.086216
H -1.762774 -2.284610 0.496913
H -3.370107 -2.048370 1.193740
H -1.938441 -1.538080 2.096546
C -2.664205 1.194886 2.623471
H -3.698806 1.098173 3.000302
H -2.307597 2.187807 2.939294
C -2.816176 3.520393 0.440445
H -2.334615 3.817557 1.385751
H -3.877734 3.817254 0.526130
H -2.382308 4.144472 -0.356616
C -2.615892 2.166959 -2.451043
H -2.239296 3.199279 -2.376720
H -3.644095 2.237342 -2.851236
H -2.017674 1.656007 -3.222320
C -2.346531 -0.982403 -2.044875
H -1.833329 -0.591348 -2.937798
H -3.341955 -1.325929 -2.380220
H -1.799550 -1.881344 -1.720350
H -2.065123 0.445383 3.164915
Na -4.805446 0.649326 -0.124722
C -6.732931 -0.981646 0.341824
C -7.019252 0.200344 1.087599
C -7.278906 1.254976 0.161171
C -7.151831 0.723683 -1.158420
C -6.814172 -0.658992 -1.045655
C -6.464366 -2.341104 0.915603
H -5.804498 -2.936465 0.264652
H -7.387465 -2.932120 1.054958
H -5.980589 -2.278338 1.903170
C -7.100246 0.300244 2.582169
H -8.108124 0.065563 2.969578
H -6.854970 1.313853 2.937133
C -7.679635 2.656919 0.515441
H -7.208496 2.993102 1.453193

H -8.770467 2.766008 0.657577
H -7.392659 3.374206 -0.269701
C -7.384460 1.470840 -2.438775
H -7.152025 2.542591 -2.334331
H -8.431988 1.410177 -2.787094
H -6.760603 1.080096 -3.258691
C -6.632928 -1.618798 -2.184247
H -6.248404 -1.115625 -3.085781
H -7.575583 -2.115373 -2.478960
H -5.923174 -2.422169 -1.930418
H -6.404258 -0.396518 3.076092
Na -9.248549 -0.450103 -0.108440
C -10.978599 -2.192504 0.582985
C -11.279303 -1.016827 1.326222
C -11.704671 -0.013911 0.410250
C -11.667188 -0.570177 -0.899749
C -11.218033 -1.916852 -0.792612
C -10.545809 -3.509042 1.153914
H -9.906135 -4.073395 0.454297
H -11.398105 -4.172275 1.398973
H -9.971085 -3.385939 2.087353
C -11.217196 -0.877456 2.817282
H -12.170276 -1.143973 3.314168
H -10.985194 0.155618 3.127146
C -12.174937 1.363396 0.769570
H -11.676818 1.747207 1.676041
H -13.262955 1.406845 0.971082
H -11.980264 2.090801 -0.036645
C -12.096954 0.116912 -2.160908
H -11.930124 1.206489 -2.116251
H -13.173137 -0.023778 -2.380610
H -11.549683 -0.256407 -3.043100
C -11.085659 -2.894079 -1.921598
H -10.834613 -2.395539 -2.873128
H -12.015982 -3.465659 -2.106329
H -10.296137 -3.640918 -1.731777
H -10.444322 -1.527537 3.260928
Na 12.964040 -1.706536 -0.288230
C 10.565703 -2.071107 0.422930
C 11.022827 -0.914891 1.130643
C 11.271626 0.119565 0.173488
C 10.968182 -0.398446 -1.125382
C 10.532576 -1.751843 -0.970707
C 10.095386 -3.355081 1.043232
H 10.252617 -4.217220 0.376799
H 9.015709 -3.338095 1.277291
H 10.614089 -3.570354 1.990158
C 11.125998 -0.780518 2.622803
H 10.178553 -0.448327 3.084824
H 11.888416 -0.043426 2.918399
C 11.682552 1.530042 0.484865
H 12.289943 1.589754 1.401148
H 10.816874 2.198609 0.643227
H 12.271196 1.977940 -0.330744
C 10.993495 0.375857 -2.411587
H 11.747490 1.177848 -2.395523
H 10.025595 0.862672 -2.628089
H 11.214439 -0.268058 -3.276950
C 10.019995 -2.641784 -2.065702
H 10.497907 -2.422872 -3.033039
H 8.932931 -2.527593 -2.222609
H 10.191017 -3.706922 -1.845917
H 11.385033 -1.735175 3.106481

(BDI*-H)Mg(H)MgCp* Transition State
141
Mg 2.519036 -1.266274 0.121756
H 1.150477 -0.892362 -1.330359
Mg -0.180430 -0.244248 -0.308335
N 0.276274 1.726112 -0.585655
N -2.205032 -0.026717 -0.186661
C -0.653439 2.626477 -0.840424
C -2.040099 2.319794 -0.726744
H -2.670217 3.168110 -0.962682
C -2.781102 1.155329 -0.444763
C -0.375446 4.076353 -1.333720
C 1.084939 4.343618 -1.709040
H 1.764697 4.262037 -0.859614
H 1.165686 5.367934 -2.104150
H 1.446092 3.656145 -2.485526
C -1.208251 4.360932 -2.601913
H -1.015155 3.613555 -3.385068
H -0.931048 5.348576 -3.001489
H -2.289524 4.377086 -2.413224
C -0.801274 5.064166 -0.233626
H -1.869531 4.967739 0.007734
H -0.618651 6.098572 -0.565696
H -0.234096 4.903412 0.693698
C -4.322944 1.368208 -0.603446
C -4.586135 1.749097 -2.076740
H -4.226380 0.967040 -2.760531
H -4.091499 2.686863 -2.360854
H -5.668470 1.870545 -2.240735
C -4.804482 2.522219 0.294847
H -5.893743 2.642274 0.186983
H -4.337975 3.482682 0.038194
H -4.593973 2.323077 1.355537
C -5.204833 0.152453 -0.296885
H -4.930850 -0.729754 -0.884813
H -6.244794 0.409621 -0.550717
H -5.184735 -0.133028 0.758953
C 1.696184 1.765644 -0.604434

C	2.353738	1.017348	-1.660496
C	3.734394	0.738330	-1.455853
H	4.270966	0.139888	-2.190946
C	4.464580	1.334496	-0.426696
H	5.540225	1.160177	-0.361229
C	3.829309	2.131955	0.512240
H	4.402371	2.562611	1.334314
C	2.435517	2.303852	0.472678
C	1.595033	0.299142	-2.654409
C	0.512692	1.008669	-3.446353
H	0.330254	2.002539	-3.029272
H	0.919534	1.205740	-4.457580
C	-0.819828	0.291676	-3.606124
H	-1.387291	0.293220	-2.663809
H	-0.684565	-0.753502	-3.923807
H	-1.455352	0.787112	-4.356597
C	2.291133	-0.794510	-3.439692
H	1.516411	-1.469326	-3.844272
H	2.882287	-1.430070	-2.751242
C	3.183614	-0.340578	-4.597721
H	3.653580	-1.201375	-5.099928
H	3.985232	0.334408	-4.262256
H	2.602061	0.204035	-5.357602
C	1.764384	2.941555	1.673116
H	0.753796	3.262358	1.378064
C	2.512696	4.155920	2.248621
H	1.891467	4.579915	3.053223
H	3.429674	3.801871	2.752150
C	2.872133	5.267891	1.274467
H	3.425783	6.068239	1.789255
H	1.976270	5.723885	0.828922
H	3.504864	4.901224	0.452263
C	1.602988	1.855077	2.754339
H	1.035713	1.021042	2.311503
H	2.602640	1.442441	2.975780
C	0.938350	2.314209	4.043681
H	0.726397	1.459467	4.703140
H	-0.016049	2.826024	3.842080
H	1.574577	3.009729	4.610660
C	-2.778877	-1.203913	0.351476
C	-2.739598	-2.401034	-0.407704
C	-3.197966	-3.586860	0.177562
H	-3.193165	-4.506831	-0.409140
C	-3.649727	-3.617625	1.490812
H	-3.998593	-4.553759	1.932424
C	-3.629423	-2.449256	2.248167
H	-3.944656	-2.487764	3.291817
C	-3.196757	-1.237315	1.707247
C	-2.258731	-2.393690	-1.846469
H	-1.478392	-1.619483	-1.928053
C	-3.368232	-1.937540	-2.809269
H	-3.645911	-0.908320	-2.541413
H	-2.940122	-1.876762	-3.823643
C	-4.612980	-2.812585	-2.826818
H	-5.074567	-2.877481	-1.829361
H	-4.389166	-3.839810	-3.153691
H	-5.366667	-2.405211	-3.517867
C	-1.599376	-3.694302	-2.311712
H	-2.323684	-4.525602	-2.293823
H	-1.335402	-3.563925	-3.375141
C	-0.355517	-4.067329	-1.522740
H	-0.586199	-4.224628	-0.459628
H	0.400783	-3.268177	-1.574065
H	0.103239	-4.990173	-1.908402
C	-3.068613	0.000610	2.573179
H	-3.374276	0.867190	1.968946
C	-1.591852	0.245525	2.932492
H	-1.071896	0.601944	2.027060
H	-1.549239	1.091120	3.635265
C	-0.856274	-0.950835	3.513391
H	-1.344194	-1.331501	4.424106
H	0.176758	-0.686168	3.776837
H	-0.811099	-1.783348	2.794274
C	-3.953589	0.009302	3.824199
H	-3.682619	-0.826827	4.490752
H	-3.703618	0.920806	4.392945
C	-5.451757	-0.008028	3.550705
H	-6.026919	0.012082	4.489057
H	-5.757786	-0.905254	2.992131
H	-5.756903	0.867444	2.957085
C	4.392006	-1.767565	1.473699
C	3.248327	-2.341827	2.107903
C	2.726937	-3.358565	1.252748
C	3.532472	-3.398497	0.076980
C	4.563969	-2.413704	0.213072
C	5.314767	-0.781078	2.117164
H	6.020994	-0.350728	1.395129
H	5.913397	-1.261270	2.911280
H	4.772790	0.056471	2.582991
C	2.792620	-2.044192	3.501960
H	2.809678	-0.966262	3.726091
H	3.442328	-2.534948	4.248760
H	1.769438	-2.402791	3.678026
C	1.571526	-4.250841	1.587668
H	0.644004	-3.687955	1.785896
H	1.779866	-4.855766	2.486137
H	1.355134	-4.950435	0.769737
C	3.414592	-4.358359	-1.067017
H	2.452648	-4.887648	-1.053992
H	4.210097	-5.123185	-1.034660
H	3.494089	-3.857368	-2.045385
C	5.687153	-2.207077	-0.753801

H	5.356660	-2.319119	-1.798217
H	6.498350	-2.939783	-0.595763
H	6.131978	-1.206486	-0.656627

(BDI*-H)Mg(H)CaCp* Transition State

141

Ca	2.810580	-0.867301	-0.070362
H	1.009079	-0.820786	-1.376933
Mg	-0.311011	-0.329894	-0.146655
N	0.113441	1.632956	-0.631036
N	-2.361549	-0.125163	-0.206288
C	-0.811383	2.467291	-1.076062
C	-2.188611	2.126149	-1.058517
H	-2.815350	2.919213	-1.445004
C	-2.932469	0.991232	-0.665480
C	-0.533714	3.885090	-1.657639
C	0.942543	4.182941	-1.939878
H	1.559506	4.184755	-1.039049
H	1.017515	5.182297	-2.395560
H	1.382608	3.461634	-2.641456
C	-1.270505	4.066205	-3.000717
H	-0.985158	3.286492	-3.722274
H	-0.998348	5.041203	-3.433562
H	-2.363616	4.048382	-2.902362
C	-1.072240	4.915961	-0.649979
H	-2.160188	4.825578	-0.521385
H	-0.851629	5.938367	-0.996296
H	-0.610040	4.784576	0.338279
C	-4.466014	1.168086	-0.924573
C	-4.663811	1.380756	-2.441022
H	-4.279731	0.525739	-3.015709
H	-4.155861	2.280858	-2.810533
H	-5.737866	1.481520	-2.662814
C	-4.986754	2.411250	-0.179441
H	-6.072462	2.507449	-0.336375
H	-4.516685	3.341420	-0.525499
H	-4.811043	2.331979	0.902955
C	-5.364286	-0.008442	-0.525072
H	-5.063857	-0.951477	-0.993621
H	-6.389454	0.215845	-0.858339
H	-5.396807	-0.170784	0.556429
C	1.529704	1.759614	-0.573971
C	2.323684	1.126616	-1.620335
C	3.745661	1.202774	-1.494675
H	4.371848	0.794534	-2.287267
C	4.359177	1.831588	-0.415024
H	5.448976	1.877157	-0.362729
C	3.583864	2.383536	0.601128
H	4.069759	2.830790	1.468993
C	2.175220	2.357673	0.533957
C	1.713934	0.174630	-2.523144
C	0.558848	0.623132	-3.407600
H	0.223938	1.617216	-3.099583
H	0.974381	0.774464	-4.420726
C	-0.652719	-0.288194	-3.512208
H	-1.280598	-0.214440	-2.612938
H	-0.370352	-1.343776	-3.645601
H	-1.291360	-0.000764	-4.360789
C	2.613369	-0.846384	-3.201766
H	1.987564	-1.715187	-3.470985
H	3.363831	-1.256532	-2.491525
C	3.369697	-0.393482	-4.452083
H	4.011654	-1.200956	-4.837522
H	4.005972	0.483435	-4.261965
H	2.672292	-0.117662	-5.256915
C	1.409391	2.907547	1.721923
H	0.364165	3.069932	1.413486
C	1.974731	4.231254	2.271056
H	1.271549	4.599822	3.034401
H	2.907468	4.016375	2.822116
C	2.236063	5.344655	1.268213
H	2.640698	6.233087	1.777044
H	1.317332	5.653266	0.750612
H	2.964846	5.040451	0.502101
C	1.415721	1.834110	2.829684
H	0.915839	0.934184	2.433747
H	2.462003	1.531351	3.004829
C	0.786456	2.251447	4.150729
H	0.675851	1.386971	4.821962
H	-0.212397	2.690182	4.003055
H	1.399216	2.995832	4.680009
C	-2.984973	-1.233794	0.421820
C	-2.952930	-2.505177	-0.203051
C	-3.508148	-3.600742	0.467976
H	-3.509732	-4.577037	-0.019462
C	-4.042661	-3.474001	1.743709
H	-4.468802	-4.341508	2.252646
C	-3.998475	-2.237475	2.381650
H	-4.370541	-2.153541	3.403663
C	-3.471103	-1.110559	1.748378
C	-2.357154	-2.681762	-1.585221
H	-1.561534	-1.927730	-1.683895
C	-3.368612	-2.352310	-2.695418
H	-3.660662	-1.297920	-2.585024
H	-2.846500	-2.417369	-3.664877
C	-4.615634	-3.224174	-2.723242
H	-5.172247	-3.162379	-1.775272
H	-4.372087	-4.284323	-2.893556
H	-5.295217	-2.909825	-3.530153
C	-1.670983	-4.032318	-1.811613
H	-2.400024	-4.858015	-1.755226
H	-1.304422	-4.041194	-2.852627
C	-0.510142	-4.290152	-0.863962

H	-0.843242	-4.326650	0.183855
H	0.236054	-3.481805	-0.933629
H	-0.006551	-5.242509	-1.090391
C	-3.307261	0.194866	2.500571
H	-3.514822	1.018976	1.801567
C	-1.836911	0.367194	2.930420
H	-1.246623	0.642333	2.038954
H	-1.777413	1.245853	3.589942
C	-1.208529	-0.839104	3.611859
H	-1.776592	-1.148298	4.502912
H	-0.181181	-0.617675	3.935507
H	-1.163194	-1.706178	2.935070
C	-4.255285	0.379934	3.690416
H	-4.066786	-0.394723	4.453000
H	-3.984329	1.332091	4.177074
C	-5.735655	0.409808	3.334172
H	-6.356357	0.565445	4.229846
H	-6.064027	-0.527417	2.860627
H	-5.958495	1.226975	2.631137
C	4.834639	-1.467283	1.447292
C	3.683857	-1.950342	2.130921
C	3.129727	-3.017448	1.368143
C	3.930388	-3.186572	0.203279
C	4.983367	-2.224834	0.249808
C	5.768756	-0.409117	1.948816
H	6.381143	0.011241	1.137760
H	6.468499	-0.809201	2.704144
H	5.234562	0.431074	2.420729
C	3.197568	-1.506475	3.476690
H	3.548263	-0.494036	3.726269
H	3.555177	-2.175278	4.280546
H	2.097680	-1.499877	3.537812
C	1.975875	-3.870506	1.798609
H	1.111550	-3.271660	2.129212
H	2.254802	-4.526963	2.642268
H	1.626567	-4.521187	0.986218
C	3.734856	-4.225218	-0.860236
H	4.278626	-5.159162	-0.631275
H	4.098150	-3.883611	-1.842707
H	2.674509	-4.493766	-0.982022
C	6.084822	-2.075532	-0.755935
H	5.765739	-2.382402	-1.764715
H	6.964986	-2.693619	-0.503062
H	6.437526	-1.034595	-0.829348

(BDI*-H)Mg(H)SrCp* Transition State

141

Sr	2.831128	-0.738406	-0.283466
H	0.812837	-0.565364	-1.621102
Mg	-0.455528	-0.288978	-0.282883
N	-0.173324	1.742483	-0.512537
N	-2.511215	-0.210386	-0.183419
C	-1.169415	2.564396	-0.797939
C	-2.520636	2.132029	-0.766337
H	-3.213301	2.919702	-1.031498
C	-3.171311	0.909836	-0.489810
C	-1.008222	4.062218	-1.192905
C	0.426290	4.483415	-1.528772
H	1.108816	4.397241	-0.681037
H	0.417623	5.538684	-1.842008
H	0.846452	3.892629	-2.354060
C	-1.850343	4.385336	-2.444489
H	-1.592191	3.722418	-3.283559
H	-1.646715	5.420583	-2.758556
H	-2.931915	4.306771	-2.273957
C	-1.523132	4.906828	-0.014112
H	-2.591140	4.726283	0.173926
H	-1.387421	5.979688	-0.225226
H	-0.979564	4.670728	0.911242
C	-4.718892	0.998865	-0.703640
C	-4.953861	1.318477	-2.196029
H	-4.531249	0.533704	-2.839950
H	-4.500377	2.271731	-2.497165
H	-6.035278	1.374583	-2.397337
C	-5.315323	2.135736	0.146588
H	-6.409739	2.150275	0.026287
H	-4.938341	3.126392	-0.140587
H	-5.097681	1.993181	1.214704
C	-5.523269	-0.268632	-0.392950
H	-5.162841	-1.145272	-0.941260
H	-6.567384	-0.093817	-0.695667
H	-5.524832	-0.520611	0.671949
C	1.234162	1.946715	-0.533286
C	1.974678	1.497231	-1.706443
C	3.395629	1.655699	-1.677153
H	3.977226	1.406137	-2.564369
C	4.059518	2.167218	-0.564152
H	5.146163	2.279986	-0.586293
C	3.339610	2.526988	0.573497
H	3.868819	2.879101	1.459559
C	1.930557	2.428281	0.599139
C	1.346400	0.645690	-2.691344
C	0.088947	1.112586	-3.411474
H	-0.294139	2.022433	-2.942735
H	0.400963	1.434585	-4.422086
C	-1.053269	0.118930	-3.555167
H	-1.602899	0.004333	-2.610079
H	-0.702473	-0.877339	-3.865426
H	-1.786301	0.464628	-4.299299
C	2.240839	-0.200249	-3.581010
H	1.646891	-1.063436	-3.929885
H	3.072808	-0.649510	-2.997011

C	2.856065	0.482531	-4.804816
H	3.509332	-0.211762	-5.356431
H	3.452147	1.366826	-4.534977
H	2.075676	0.820851	-5.502517
C	1.231527	2.766747	1.902589
H	0.157515	2.896531	1.693959
C	1.763402	4.043813	2.580895
H	1.101268	4.267142	3.432184
H	2.745911	3.821155	3.033684
C	1.887229	5.289222	1.716508
H	2.279964	6.130247	2.308440
H	0.916846	5.601499	1.306699
H	2.571953	5.130981	0.869944
C	1.388711	1.562202	2.854703
H	0.895598	0.691946	2.388650
H	2.460733	1.303988	2.899066
C	0.868230	1.765780	4.269704
H	0.889879	0.821219	4.832931
H	-0.169587	2.132289	4.271862
H	1.477202	2.489783	4.830348
C	-3.036723	-1.401029	0.377081
C	-2.942846	-2.619909	-0.339175
C	-3.410896	-3.793348	0.263730
H	-3.367289	-4.730577	-0.293441
C	-3.916620	-3.791964	1.557317
H	-4.275511	-4.718557	2.010945
C	-3.927315	-2.604296	2.283600
H	-4.271292	-2.617998	3.318703
C	-3.487123	-1.404387	1.722457
C	-2.380925	-2.663702	-1.746579
H	-1.637153	-1.854363	-1.821945
C	-3.448632	-2.330998	-2.802571
H	-3.802633	-1.306802	-2.614330
H	-2.956778	-2.300191	-3.789419
C	-4.639548	-3.277295	-2.850589
H	-5.170096	-3.307330	-1.886369
H	-4.336792	-4.307868	-3.092419
H	-5.361106	-2.958674	-3.618435
C	-1.618139	-3.949993	-2.081975
H	-2.297540	-4.818858	-2.080734
H	-1.269532	-3.857716	-3.125138
C	-0.427249	-4.216046	-1.173365
H	-0.737020	-4.365473	-0.128284
H	0.267341	-3.360088	-1.180688
H	0.129074	-5.111526	-1.489825
C	-3.366658	-0.152233	2.568542
H	-3.642669	0.711525	1.945239
C	-1.891033	0.063400	2.961611
H	-1.345072	0.422455	2.071961
H	-1.849141	0.898461	3.677941
C	-1.189144	-1.156947	3.538847
H	-1.718002	-1.555740	4.418420
H	-0.165171	-0.912054	3.853181
H	-1.123769	-1.969760	2.799381
C	-4.277545	-0.112984	3.800399
H	-4.018003	-0.931205	4.493380
H	-4.040704	0.813431	4.350445
C	-5.770103	-0.142246	3.499124
H	-6.362851	-0.091621	4.425314
H	-6.064366	-1.058414	2.965674
H	-6.065652	0.711324	2.870206
C	4.816244	-1.278905	1.561439
C	3.711009	-2.072171	1.982394
C	3.504535	-3.093305	1.011641
C	4.477157	-2.927581	-0.014054
C	5.288124	-1.804586	0.325335
C	5.420744	-0.137813	2.322416
H	5.906847	0.591396	1.656039
H	6.190811	-0.479414	3.037317
H	4.667862	0.412621	2.908377
C	2.960984	-1.942555	3.273501
H	3.021046	-0.924289	3.685855
H	3.359231	-2.622345	4.048657
H	1.893478	-2.187768	3.157368
C	2.494582	-4.195051	1.118716
H	1.525805	-3.834715	1.500747
H	2.831535	-4.993127	1.804860
H	2.301050	-4.671936	0.146945
C	4.659868	-3.810396	-1.212612
H	5.042937	-3.254467	-2.084552
H	3.717118	-4.290677	-1.519634
H	5.381595	-4.625335	-1.023066
C	6.467175	-1.304399	-0.453835
H	6.637938	-0.227769	-0.296200
H	6.345100	-1.459883	-1.538430
H	7.403081	-1.818260	-0.168552

(BDI*-H)Mg(H)BaCp* Transition State
141

Ba	2.904201	-0.520253	-0.469853
H	0.609726	-0.239601	-1.794815
Mg	-0.580255	-0.212483	-0.363026
N	-0.467076	1.844302	-0.343825
N	-2.632118	-0.303829	-0.160610
C	-1.530000	2.618170	-0.482741
C	-2.843766	2.082839	-0.455408
H	-3.603787	2.839083	-0.599048
C	-3.386956	0.788302	-0.310986
C	-1.492648	4.161762	-0.682327
C	-0.112553	4.725079	-1.036852
H	0.628359	4.572654	-0.249589
H	-0.207255	5.809818	-1.199242

H	0.287303	4.281422	-1.958851
C	-2.432502	4.587888	-1.828897
H	-2.191160	4.058780	-2.762752
H	-2.310658	5.666475	-2.012316
H	-3.494450	4.416998	-1.609111
C	-1.983908	4.800173	0.628811
H	-3.023223	4.516361	0.848030
H	-1.932916	5.898843	0.562101
H	-1.367122	4.481528	1.480650
C	-4.942478	0.773567	-0.481889
C	-5.236269	1.219967	-1.930782
H	-4.770126	0.537524	-2.656065
H	-4.861460	2.230271	-2.140674
H	-6.323220	1.211995	-2.108972
C	-5.607285	1.768143	0.487447
H	-6.702018	1.698176	0.392528
H	-5.326170	2.811170	0.289795
H	-5.346833	1.544813	1.531854
C	-5.636531	-0.579513	-0.287962
H	-5.221993	-1.365006	-0.928434
H	-6.698470	-0.460522	-0.553614
H	-5.591042	-0.935921	0.745735
C	0.919630	2.149133	-0.423872
C	1.602668	1.914109	-1.692365
C	2.996070	2.231649	-1.747244
H	3.514063	2.181433	-2.704983
C	3.704818	2.639697	-0.620829
H	4.767854	2.881390	-0.704092
C	3.058120	2.734640	0.613194
H	3.626801	3.005318	1.503860
C	1.664043	2.517775	0.717354
C	0.972677	1.149401	-2.744781
C	-0.371228	1.582756	-3.314265
H	-0.807599	2.369396	-2.694373
H	-0.162279	2.078537	-4.280140
C	-1.420171	0.505212	-3.544094
H	-1.892385	0.202107	-2.599136
H	-0.996437	-0.396290	-4.012695
H	-2.229353	0.873945	-4.192086
C	1.866313	0.517805	-3.798491
H	1.337779	-0.361948	-4.206785
H	2.786546	0.108942	-3.333145
C	2.295558	1.402950	-4.971882
H	2.976272	0.859417	-5.646111
H	2.808095	2.317731	-4.638443
H	1.428419	1.719329	-5.570305
C	1.042558	2.620513	2.098192
H	-0.050801	2.684880	1.977984
C	1.526177	3.839432	2.907074
H	0.913090	3.896805	3.820268
H	2.553040	3.643004	3.263591
C	1.494376	5.190891	2.210118
H	1.864741	5.979283	2.883359
H	0.476535	5.470790	1.905820
H	2.126267	5.199271	1.309524
C	1.361358	1.320092	2.866192
H	0.891108	0.479235	2.325880
H	2.449755	1.147280	2.800035
C	0.947302	1.294605	4.329686
H	1.115440	0.298584	4.764888
H	-0.118663	1.537974	4.454363
H	1.525937	2.010020	4.931727
C	-3.044231	-1.578491	0.297535
C	-2.879976	-2.715591	-0.531942
C	-3.233721	-3.973140	-0.028938
H	-3.135942	-4.849198	-0.672183
C	-3.695933	-4.130395	1.271349
H	-3.966085	-5.120407	1.645586
C	-3.777325	-3.018126	2.104797
H	-4.085887	-3.154067	3.142212
C	-3.450234	-1.740523	1.647657
C	-2.370536	-2.586241	-1.954224
H	-1.696981	-1.714239	-1.980242
C	-3.502723	-2.251879	-2.940559
H	-3.929513	-1.281189	-2.648086
H	-3.054483	-2.093066	-3.935863
C	-4.615616	-3.285463	-3.036284
H	-5.105861	-3.441724	-2.063011
H	-4.241375	-4.263069	-3.377662
H	-5.388213	-2.962421	-3.750787
C	-1.521258	-3.770028	-2.430759
H	-2.129545	-4.688109	-2.491047
H	-1.219434	-3.554816	-3.470345
C	-0.280387	-4.021756	-1.586763
H	-0.538368	-4.288315	-0.551007
H	0.343910	-3.114379	-1.535868
H	0.331173	-4.837576	-2.001618
C	-3.397115	-0.565704	2.605035
H	-3.759640	0.328074	2.075729
C	-1.928683	-0.273716	2.978018
H	-1.441735	0.217040	2.117616
H	-1.925601	0.482254	3.779479
C	-1.121679	-1.492120	3.401498
H	-1.604208	-2.036170	4.228263
H	-0.117226	-1.205048	3.738897
H	-1.002816	-2.201667	2.568386
C	-4.264535	-0.713765	3.859972
H	-3.916023	-1.567305	4.465919
H	-4.082572	0.175258	4.487191
C	-5.760115	-0.838328	3.600726
H	-6.320605	-0.919380	4.544698
H	-6.000931	-1.724076	2.994146

H	-6.145043	0.040909	3.062091
C	4.869372	-1.273622	1.584522
C	3.766877	-2.132557	1.858117
C	3.671033	-3.078521	0.799479
C	4.712209	-2.804047	-0.130087
C	5.453298	-1.688206	0.355631
C	5.370568	-0.166194	2.461753
H	5.751859	0.688656	1.878882
H	6.198850	-0.494933	3.115186
H	4.581304	0.220746	3.124814
C	2.920543	-2.132416	3.094252
H	2.927033	-1.155346	3.599468
H	3.275273	-2.873323	3.833731
H	1.870367	-2.383157	2.877305
C	2.693130	-4.212145	0.735403
H	1.703724	-3.930350	1.130213
H	3.033097	-5.083189	1.324923
H	2.539812	-4.569507	-0.294190
C	5.014315	-3.583187	-1.374771
H	4.116978	-4.076754	-1.781860
H	5.756519	-4.382503	-1.197343
H	5.432266	-2.948571	-2.174762
C	6.666168	-1.086352	-0.286843
H	7.601713	-1.562570	0.059251
H	6.762879	-0.011335	-0.062776
H	6.652850	-1.192957	-1.384292

(BDI*-H)Mg(H)MgCp*

141

g	2.641424	-1.015168	0.150275
H	0.869158	-1.109742	0.285795
Mg	-0.333907	-0.249190	-0.772345
N	0.227429	1.733055	-0.518989
N	-2.249598	0.070021	-0.064208
C	-0.665313	2.696675	-0.598747
C	-2.038311	2.458540	-0.313889
H	-2.644390	3.354241	-0.376126
C	-2.788938	1.292836	-0.061148
C	-0.351023	4.152183	-1.049050
C	1.041730	4.316490	-1.661936
H	1.845052	4.117070	-0.950643
H	1.158308	5.354176	-2.010162
H	1.191945	3.654994	-2.526107
C	-1.359893	4.598862	-2.127040
H	-1.386690	3.890181	-2.967479
H	-1.058884	5.581944	-2.520830
H	-2.382716	4.701392	-1.741799
C	-0.483328	5.083237	0.168393
H	-1.490771	5.034208	0.606234
H	-0.290086	6.125811	-0.130968
H	0.236314	4.822066	0.957612
C	-4.327110	1.557639	0.018819
C	-4.760123	2.019535	-1.389925
H	-4.528031	1.252697	-2.143314
H	-4.257643	2.946152	-1.696966
H	-5.847614	2.194280	-1.407916
C	-4.653841	2.670420	1.029180
H	-5.744701	2.810208	1.083484
H	-4.211412	3.637626	0.755495
H	-4.298799	2.415330	2.038202
C	-5.195762	0.344599	0.369295
H	-5.029168	-0.501752	-0.306415
H	-6.252767	0.639223	0.278339
H	-5.037730	-0.011401	1.392009
C	1.577731	1.594091	-0.847617
C	1.839869	0.693689	-1.961783
C	3.187275	0.291273	-2.146451
H	3.432546	-0.379944	-2.968380
C	4.223525	0.772943	-1.338624
H	5.250340	0.467133	-1.541351
C	3.942681	1.610000	-0.262248
H	4.750449	1.935115	0.393630
C	2.618553	1.996928	0.019474
C	0.716786	0.114338	-2.714467
C	-0.145319	1.141517	-3.458620
H	-0.159473	2.087309	-2.906771
H	0.337831	1.395335	-4.422877
C	-1.584762	0.724775	-3.725958
H	-2.152046	0.643894	-2.785459
H	-1.642057	-0.248837	-4.237278
H	-2.109110	1.458904	-4.358027
C	1.055784	-1.099039	-3.575801
H	0.136352	-1.684864	-3.748256
H	1.708798	-1.778612	-2.998254
C	1.688657	-0.837415	-4.947863
H	1.992407	-1.779452	-5.432116
H	2.576740	-0.189517	-4.885218
H	0.979673	-0.338247	-5.626007
C	2.318296	2.688112	1.333935
H	1.354531	3.208322	1.232099
C	3.376129	3.706811	1.776813
H	3.035694	4.153116	2.724162
H	4.308140	3.171695	2.031602
C	3.678925	4.826142	0.791531
H	4.462702	5.491508	1.184927
H	2.788980	5.444002	0.599726
H	4.028095	4.436569	-0.176868
C	2.127942	1.587145	2.392678
H	1.351175	0.900913	2.021461
H	3.062318	0.999912	2.445940
C	1.748938	2.072878	3.783417
H	1.508455	1.224852	4.441223

H	0.863705	2.727299	3.746261
H	2.563151	2.636617	4.262236
C	-2.747465	-1.110870	0.518163
C	-2.867431	-2.272159	-0.284219
C	-3.250783	-3.477146	0.316012
H	-3.365968	-4.368795	-0.302857
C	-3.479499	-3.560979	1.683267
H	-3.775287	-4.509427	2.137063
C	-3.300007	-2.428579	2.475374
H	-3.434733	-2.511028	3.554903
C	-2.930438	-1.201001	1.924914
C	-2.624711	-2.213986	-1.780483
H	-1.920394	-1.385970	-1.984064
C	-3.893091	-1.816189	-2.555709
H	-4.170440	-0.796886	-2.247143
H	-3.632045	-1.744815	-3.624979
C	-5.087832	-2.740699	-2.375021
H	-5.404975	-2.787027	-1.322133
H	-4.866237	-3.769351	-2.699670
H	-5.945466	-2.387620	-2.967790
C	-1.955677	-3.467721	-2.350445
H	-2.634642	-4.334146	-2.287913
H	-1.798416	-3.297289	-3.429074
C	-0.629357	-3.802261	-1.686722
H	-0.759488	-4.062910	-0.626786
H	0.065426	-2.948018	-1.728895
H	-0.130876	-4.645997	-2.185655
C	-2.614523	-0.010869	2.810617
H	-2.996219	0.892494	2.312806
C	-1.090059	0.182097	2.901814
H	-0.723345	0.464275	1.902802
H	-0.896138	1.052691	3.548265
C	-0.318631	-1.027256	3.402050
H	-0.609486	-1.314181	4.424801
H	0.761122	-0.825734	3.409894
H	-0.479850	-1.896579	2.749540
C	-3.263047	-0.045217	4.198018
H	-2.912722	-0.925250	4.762958
H	-2.880128	0.824944	4.757947
C	-4.785534	-0.003975	4.195754
H	-5.186095	-0.020320	5.221072
H	-5.218048	-0.859930	3.656076
H	-5.157864	0.911122	3.709960
C	4.642321	-1.700216	1.138797
C	3.585955	-2.157595	1.995795
C	2.821750	-3.123290	1.277486
C	3.386957	-3.252117	-0.023847
C	4.523396	-2.390033	-0.105611
C	5.757999	-0.797511	1.561374
H	6.256900	-0.329777	0.700408
H	6.532090	-1.347612	2.125369

H	5.405461	0.015344	2.215618
C	3.460972	-1.813101	3.447826
H	3.556739	-0.733153	3.639117
H	4.248203	-2.309639	4.041982
H	2.494595	-2.131662	3.860544
C	1.617771	-3.862288	1.772484
H	1.451449	-3.686754	2.843848
H	1.725902	-4.949950	1.633026
H	0.696237	-3.554903	1.250228
C	2.954274	-4.209089	-1.091538
H	1.980180	-4.656938	-0.855998
H	3.676758	-5.036246	-1.206995
H	2.863101	-3.729530	-2.079812
C	5.485005	-2.363617	-1.250847
H	4.980422	-2.246655	-2.223629
H	6.058931	-3.305132	-1.303796
H	6.217078	-1.549820	-1.154693

(BDI*-H)Mg(H)CaCp* (**3**)

141

Ca	-2.697691	-0.726044	-0.066845
H	-0.633377	-1.274528	-0.069194
Mg	0.489000	-0.214518	0.827491
N	-0.035087	1.767951	0.411074
N	2.376692	0.000015	-0.001429
C	0.887068	2.708376	0.351229
C	2.236048	2.404753	0.029270
H	2.866354	3.283993	-0.024637
C	2.945143	1.197340	-0.147825
C	0.629319	4.212376	0.658181
C	-0.721589	4.481253	1.326013
H	-1.569732	4.238489	0.683459
H	-0.790611	5.551755	1.572657
H	-0.839594	3.915412	2.260398
C	1.707327	4.748961	1.622113
H	1.770085	4.135752	2.532760
H	1.448041	5.776261	1.921294
H	2.707735	4.784153	1.171704
C	0.708814	5.000358	-0.660610
H	1.689436	4.879821	-1.143080
H	0.549434	6.073832	-0.470194
H	-0.057625	4.669505	-1.376332
C	4.481846	1.408910	-0.333173
C	5.002011	1.986755	1.001295
H	4.799480	1.296454	1.833207
H	4.534700	2.948914	1.249131
H	6.091759	2.136981	0.943361
C	4.778500	2.412628	-1.460151
H	5.867631	2.512103	-1.588023
H	4.378653	3.414652	-1.254605
H	4.358496	2.076239	-2.419128

C	5.300191	0.143566	-0.612252
H	5.147903	-0.632001	0.146914
H	6.367617	0.413621	-0.602493
H	5.078545	-0.299930	-1.588016
C	-1.365944	1.715851	0.828624
C	-1.588333	0.950557	2.050641
C	-2.937371	0.633741	2.361830
H	-3.150622	0.063310	3.264923
C	-4.005989	1.066690	1.576033
H	-5.027794	0.832771	1.877264
C	-3.768283	1.768546	0.394075
H	-4.606437	2.059920	-0.239200
C	-2.449649	2.067078	-0.007725
C	-0.446272	0.420359	2.795582
C	0.504645	1.479607	3.362916
H	0.516644	2.360304	2.712582
H	0.097878	1.854983	4.323016
C	1.941661	1.029214	3.582899
H	2.438021	0.815766	2.623539
H	1.996786	0.118629	4.199522
H	2.536414	1.805546	4.089621
C	-0.758950	-0.689499	3.794268
H	0.151980	-1.290829	3.959959
H	-1.472968	-1.397328	3.335616
C	-1.279688	-0.268425	5.173776
H	-1.568771	-1.145538	5.774689
H	-2.154578	0.397064	5.107082
H	-0.510336	0.275553	5.742842
C	-2.214977	2.572148	-1.418350
H	-1.225351	3.050608	-1.448777
C	-3.260546	3.573793	-1.921586
H	-2.966639	3.884386	-2.936279
H	-4.227202	3.054892	-2.049686
C	-3.453453	4.817387	-1.066608
H	-4.231597	5.467607	-1.494520
H	-2.527960	5.408826	-1.003662
H	-3.757814	4.565721	-0.039388
C	-2.144863	1.333822	-2.331780
H	-1.343071	0.679012	-1.947017
H	-3.102080	0.781272	-2.249440
C	-1.877396	1.611858	-3.803288
H	-1.710012	0.674949	-4.353133
H	-0.981822	2.239903	-3.927908
H	-2.721705	2.127502	-4.283056
C	2.811455	-1.243033	-0.497496
C	2.941056	-2.332692	0.397800
C	3.272742	-3.593936	-0.110072
H	3.396924	-4.431099	0.578975
C	3.436587	-3.802673	-1.473305
H	3.694219	-4.793571	-1.853704
C	3.234895	-2.742009	-2.354036

H	3.309916	-2.923580	-3.427232
C	2.914032	-1.463193	-1.897774
C	2.759709	-2.142600	1.891912
H	2.082623	-1.282535	2.052811
C	4.067468	-1.709987	2.578730
H	4.355147	-0.730229	2.167677
H	3.852787	-1.535869	3.646532
C	5.232645	-2.677976	2.436690
H	5.505223	-2.827187	1.380894
H	5.001350	-3.666900	2.862236
H	6.121528	-2.295300	2.961145
C	2.088968	-3.328376	2.592558
H	2.742565	-4.215641	2.561210
H	1.997132	-3.072128	3.661891
C	0.718026	-3.674652	2.030279
H	0.776555	-3.978451	0.975035
H	0.037854	-2.810058	2.077235
H	0.245857	-4.491770	2.595688
C	2.552920	-0.356745	-2.870569
H	2.965332	0.586728	-2.483442
C	1.024912	-0.162357	-2.888772
H	0.722791	0.223336	-1.902590
H	0.793766	0.636444	-3.611640
C	0.222685	-1.413256	-3.206706
H	0.459080	-1.820782	-4.202131
H	-0.857232	-1.204504	-3.187255
H	0.408388	-2.199691	-2.462493
C	3.116531	-0.526531	-4.284781
H	2.724835	-1.449181	-4.744895
H	2.708917	0.294633	-4.898778
C	4.636704	-0.508005	-4.376104
H	4.974677	-0.621473	-5.417676
H	5.092413	-1.318952	-3.788255
H	5.046520	0.440829	-3.997002
C	-5.010480	-1.577186	-0.888353
C	-4.051471	-2.100933	-1.806351
C	-3.236717	-3.037034	-1.106712
C	-3.686144	-3.084716	0.242283
C	-4.788856	-2.194116	0.375822
C	-6.108531	-0.615755	-1.227609
H	-6.455248	-0.060068	-0.343120
H	-6.991456	-1.128036	-1.649895
H	-5.791268	0.129705	-1.974998
C	-3.997753	-1.808120	-3.275704
H	-4.397928	-0.810295	-3.513922
H	-4.594592	-2.532781	-3.857830
H	-2.972127	-1.853046	-3.673407
C	-2.148333	-3.885788	-1.689818
H	-1.833220	-3.520691	-2.676841
H	-2.479037	-4.930984	-1.822171
H	-1.250654	-3.906651	-1.052101

C -3.135074 -3.964942 1.322191
H -2.099270 -4.265502 1.108670
H -3.723960 -4.892789 1.437121
H -3.136366 -3.469073 2.306795
C -5.617956 -2.033138 1.613031
H -5.003937 -1.919757 2.521995
H -6.265991 -2.911535 1.780183
H -6.281179 -1.158061 1.551794

(BDI*-H)Mg(H)SrCp*
141
Sr -2.584656 -0.725314 0.160880
H -0.381226 -1.341019 0.379721
Mg 0.742436 -0.074896 0.953502
N 0.224824 1.831157 0.233329
N 2.536058 -0.094832 -0.086307
C 1.169884 2.702113 -0.064357
C 2.468072 2.282971 -0.460979
H 3.116919 3.110231 -0.722281
C 3.118736 1.031247 -0.498920
C 1.010482 4.248870 0.033289
C -0.265690 4.697496 0.749997
H -1.175678 4.418017 0.216799
H -0.257985 5.794807 0.835742
H -0.336594 4.284847 1.765494
C 2.189398 4.846030 0.829973
H 2.289011 4.368408 1.815172
H 2.012248 5.920870 0.989648
H 3.151511 4.747093 0.311116
C 1.028177 4.842109 -1.386176
H 1.957794 4.592014 -1.917138
H 0.947061 5.939763 -1.336820
H 0.188874 4.472423 -1.992807
C 4.630651 1.134390 -0.878150
C 5.313358 1.910384 0.269504
H 5.179511 1.390566 1.229471
H 4.909629 2.924941 0.384298
H 6.394717 1.989835 0.075096
C 4.828136 1.911599 -2.190486
H 5.899197 1.941122 -2.444379
H 4.475780 2.949921 -2.127155
H 4.299626 1.430829 -3.026435
C 5.373974 -0.199077 -1.010510
H 5.284556 -0.820504 -0.112311
H 6.443424 0.014545 -1.162309
H 5.030324 -0.795528 -1.861452
C -1.070950 1.916940 0.751960
C -1.224456 1.348185 2.085775
C -2.555386 1.124118 2.528065
H -2.717659 0.696436 3.517222
C -3.666123 1.489906 1.766086
H -4.670977 1.341466 2.166361
C -3.495311 2.033684 0.492073
H -4.369628 2.290656 -0.106136
C -2.202707 2.219937 -0.039942
C -0.043039 0.907878 2.828866
C 0.974143 2.009205 3.144865
H 0.945787 2.778463 2.366709
H 0.663053 2.541225 4.065917
C 2.416455 1.549681 3.309239
H 2.826018 1.182521 2.354665
H 2.506503 0.737597 4.047523
H 3.068833 2.371386 3.644863
C -0.300988 -0.015540 4.014815
H 0.611412 -0.603656 4.217620
H -1.054179 -0.771735 3.727600
C -0.720613 0.638639 5.336984
H -0.975969 -0.120267 6.093937
H -1.590994 1.302840 5.218878
H 0.092155 1.252426 5.754523
C -2.058502 2.564139 -1.511843
H -1.071984 3.026599 -1.656835
C -3.128065 3.519365 -2.053283
H -2.908151 3.700890 -3.116799
H -4.106908 3.007798 -2.047020
C -3.241419 4.861000 -1.344364
H -4.049584 5.465960 -1.782886
H -2.310772 5.441007 -1.433326
H -3.458213 4.742449 -0.272002
C -2.047436 1.241431 -2.298828
H -1.233448 0.620706 -1.886355
H -3.009552 0.719404 -2.124249
C -1.836168 1.360726 -3.800438
H -1.727720 0.366562 -4.257273
H -0.924658 1.936492 -4.023885
H -2.682099 1.857773 -4.296571
C 2.869857 -1.425530 -0.397419
C 3.060543 -2.350421 0.658595
C 3.295080 -3.695005 0.350301
H 3.470926 -4.405316 1.159937
C 3.296204 -4.145368 -0.963527
H 3.478266 -5.198954 -1.186655
C 3.028877 -3.244569 -1.992138
H 2.976462 -3.612690 -3.017868
C 2.806185 -1.890647 -1.739114
C 3.045878 -1.897131 2.106535
H 2.390544 -1.008930 2.186462
C 4.422395 -1.377650 2.557952
H 4.665570 -0.495925 1.945811
H 4.328747 -1.008177 3.593019
C 5.559846 -2.384204 2.468528
H 5.704745 -2.736191 1.435774

H 5.377241 -3.269516 3.097306
H 6.505871 -1.932862 2.804567
C 2.458431 -2.925516 3.078876
H 3.106366 -3.815802 3.134496
H 2.490764 -2.480007 4.087978
C 1.031888 -3.342672 2.750598
H 0.966019 -3.825822 1.765102
H 0.360245 -2.471061 2.723711
H 0.636702 -4.042169 3.502675
C 2.388862 -0.948663 -2.852490
H 2.885226 0.018059 -2.682444
C 0.881138 -0.654271 -2.754099
H 0.709782 -0.103636 -1.815930
H 0.620310 0.041388 -3.567382
C -0.023271 -1.875157 -2.793630
H 0.091514 -2.453282 -3.723729
H -1.083702 -1.582548 -2.731965
H 0.180377 -2.545498 -1.946787
C 2.786060 -1.393660 -4.263339
H 2.311115 -2.358186 -4.509440
H 2.346110 -0.669322 -4.970004
C 4.286800 -1.478659 -4.508859
H 4.504566 -1.791894 -5.541522
H 4.772047 -2.198882 -3.833116
H 4.771533 -0.503079 -4.349887
C -5.160772 -1.303081 -0.686360
C -4.313602 -2.010725 -1.589286
C -3.661537 -3.047362 -0.861381
C -4.100399 -2.975612 0.490342
C -5.032092 -1.902870 0.598149
C -6.087774 -0.184342 -1.055064
H -6.327046 0.453460 -0.190366
H -7.049424 -0.556116 -1.451937
H -5.661680 0.470607 -1.832883
C -4.236797 -1.789883 -3.070606
H -4.472288 -0.750477 -3.347688
H -4.953790 -2.429054 -3.616435
H -3.239748 -2.018800 -3.478547
C -2.744518 -4.091923 -1.423111
H -2.357504 -3.807526 -2.411938
H -3.260315 -5.060781 -1.547277
H -1.872212 -4.278715 -0.775467
C -3.700846 -3.912342 1.590792
H -2.661689 -4.261998 1.480297
H -4.336165 -4.816302 1.614171
H -3.786946 -3.444347 2.584521
C -5.796219 -1.537043 1.835160
H -5.161891 -1.521762 2.737774
H -6.611325 -2.253717 2.039328
H -6.262751 -0.544702 1.748049

(BDI*-H)Mg(H)BaCp*
141
Ba -2.500237 -0.766797 0.451951
H -0.093868 -1.295705 0.878109
Mg 1.017676 0.088210 1.040775
N 0.482430 1.858011 0.021561
N 2.679272 -0.212786 -0.175898
C 1.420698 2.613051 -0.516423
C 2.648661 2.059212 -0.970551
H 3.291630 2.791537 -1.444302
C 3.243901 0.785431 -0.856540
C 1.345135 4.164269 -0.656702
C 0.165213 4.801772 0.081638
H -0.803861 4.497667 -0.315975
H 0.233893 5.895858 -0.017938
H 0.170935 4.564935 1.153868
C 2.621524 4.796662 -0.061988
H 2.781800 4.473519 0.976254
H 2.518720 5.892901 -0.064870
H 3.526376 4.550281 -0.632063
C 1.268658 4.536598 -2.147799
H 2.131072 4.147203 -2.707357
H 1.255580 5.632413 -2.260718
H 0.358889 4.142466 -2.623154
C 4.694589 0.730900 -1.434919
C 5.552063 1.671644 -0.560333
H 5.526108 1.361716 0.494558
H 5.207890 2.713169 -0.606815
H 6.600618 1.641510 -0.896656
C 4.734615 1.232592 -2.888281
H 5.763308 1.163187 -3.275176
H 4.414820 2.278822 -2.984869
H 4.090560 0.626461 -3.541803
C 5.371601 -0.643290 -1.397607
H 5.389101 -1.075447 -0.390870
H 6.415487 -0.524185 -1.727037
H 4.895088 -1.371208 -2.061476
C -0.760906 2.096906 0.618844
C -0.818551 1.762077 2.035732
C -2.113609 1.659139 2.608030
H -2.204726 1.403453 3.663811
C -3.272774 1.938554 1.882611
H -4.247111 1.893133 2.375448
C -3.191462 2.280360 0.531253
H -4.103706 2.486815 -0.028482
C -1.942830 2.337014 -0.120810
C 0.411452 1.418718 2.751898
C 1.480210 2.515213 2.785054
H 1.387478 3.151936 1.899984
H 1.281848 3.198664 3.634640
C 2.921435 2.030131 2.874883

H	3.223238	1.508800	1.951612
H	3.067041	1.334623	3.716319
H	3.625261	2.865907	3.016145
C	0.234972	0.726508	4.098836
H	1.154364	0.162683	4.336973
H	-0.545780	-0.050521	4.006238
C	-0.075839	1.614646	5.310396
H	-0.277109	1.009016	6.208802
H	-0.947324	2.265909	5.140332
H	0.771721	2.275473	5.548461
C	-1.904823	2.506611	-1.630632
H	-0.927207	2.932096	-1.895148
C	-2.997586	3.422245	-2.195260
H	-2.864840	3.469808	-3.287287
H	-3.981395	2.943502	-2.047535
C	-3.026753	4.842251	-1.648624
H	-3.865738	5.408361	-2.081236
H	-2.102133	5.385499	-1.894400
H	-3.141830	4.859202	-0.554358
C	-1.963780	1.114314	-2.280169
H	-1.153859	0.513175	-1.835429
H	-2.933601	0.645071	-2.024014
C	-1.795248	1.077624	-3.791473
H	-1.765058	0.039449	-4.152852
H	-0.856730	1.568429	-4.093401
H	-2.623928	1.578382	-4.312504
C	2.928415	-1.594146	-0.261655
C	3.230387	-2.311973	0.922322
C	3.383123	-3.700858	0.854833
H	3.647686	-4.253007	1.758225
C	3.191553	-4.392637	-0.334511
H	3.311066	-5.477813	-0.368524
C	2.815066	-3.690084	-1.476974
H	2.616750	-4.242308	-2.396976
C	2.672863	-2.301939	-1.467589
C	3.417636	-1.590222	2.243790
H	2.772231	-0.691405	2.240617
C	4.840542	-1.023585	2.389264
H	4.991307	-0.287881	1.585201
H	4.891164	-0.453187	3.331981
C	5.957256	-2.056156	2.347938
H	5.948154	-2.619635	1.402345
H	5.874374	-2.785986	3.168233
H	6.940782	-1.570395	2.439178
C	2.982736	-2.397214	3.471379
H	3.635119	-3.276226	3.603860
H	3.163495	-1.766298	4.358643
C	1.524419	-2.832311	3.445457
H	1.313258	-3.493928	2.593018
H	0.855275	-1.964276	3.347806
H	1.251231	-3.363966	4.369763
C	2.157454	-1.564445	-2.688741
H	2.707675	-0.615318	-2.765566
C	0.687408	-1.158564	-2.487584
H	0.652500	-0.439301	-1.654007
H	0.367712	-0.603557	-3.383175
C	-0.275616	-2.302199	-2.210835
H	-0.254760	-3.070585	-2.998850
H	-1.317640	-1.940348	-2.173557
H	-0.042419	-2.792855	-1.253791
C	2.363069	-2.289846	-4.022345
H	1.827655	-3.254163	-4.021881
H	1.866892	-1.686945	-4.802120
C	3.818397	-2.508369	-4.414685
H	3.896116	-3.023450	-5.384494
H	4.356084	-3.115368	-3.670988
H	4.353876	-1.550783	-4.504696
C	-5.273355	-0.938801	-0.641074
C	-4.531182	-1.873030	-1.420364
C	-4.156442	-2.954450	-0.573133
C	-4.660995	-2.684460	0.729463
C	-5.353135	-1.439415	0.686592
C	-5.943642	0.295149	-1.163572
H	-6.116013	1.037787	-0.369625
H	-6.930306	0.071879	-1.607962
H	-5.353255	0.791445	-1.950930
C	-4.326253	-1.805168	-2.904006
H	-4.350296	-0.770344	-3.278386
H	-5.115196	-2.353938	-3.449398
H	-3.366318	-2.243908	-3.219725
C	-3.433275	-4.198500	-0.993790
H	-2.860157	-4.047031	-1.920610
H	-4.130042	-5.034560	-1.184380
H	-2.723515	-4.554983	-0.228337
C	-4.545118	-3.585150	1.922304
H	-3.630815	-4.200283	1.892733
H	-5.391913	-4.291894	1.991559
H	-4.535523	-3.023345	2.871232
C	-6.089251	-0.811411	1.831309
H	-5.547186	-0.908825	2.787637
H	-7.080013	-1.273761	1.989921
H	-6.264550	0.262144	1.664814

$MgCp_2$

21

Mg	-0.000114	0.000016	-0.001165
C	2.020265	0.057720	-1.208144
C	2.018447	0.663511	1.011809
C	-2.019990	0.061396	-1.207978
C	-2.018630	0.660592	1.013658
C	-2.019515	-1.130398	-0.431715
C	2.019671	-1.131489	-0.428015

C -2.018794 -0.759899 0.941485
C 2.018548 -0.756966 0.944012
C -2.019277 1.168347 -0.314912
C 2.019476 1.167151 -0.318236
H 2.036833 2.218569 -0.605227
H 2.034305 1.261414 1.923074
H 2.034421 -1.438980 1.794163
H 2.037189 -2.150751 -0.813929
H 2.038835 0.109718 -2.296761
H -2.037782 0.116696 -2.296460
H -2.036317 2.220665 -0.598589
H -2.035135 1.255530 1.926844
H -2.035398 -1.444314 1.789685
H -2.036787 -2.148524 -0.820608

(BDI*)MgMgCp
126
Mg 0.008911 -0.603611 -0.038899
Mg 1.505896 -2.745308 0.820675
N -1.812443 0.346720 -0.198084
N 1.082742 1.094870 -0.582156
C -1.961250 1.631479 -0.509006
C -0.866136 2.464019 -0.829223
H -1.160871 3.473777 -1.086518
C 0.532131 2.267858 -0.869449
C -3.323530 2.393662 -0.526159
C -4.582038 1.564338 -0.247568
H -4.740395 0.769718 -0.983217
H -5.450388 2.239930 -0.294480
H -4.576059 1.103253 0.745292
C -3.525015 3.045112 -1.907827
H -2.763538 3.799918 -2.143862
H -4.507071 3.541842 -1.943316
H -3.502201 2.286615 -2.704839
C -3.259956 3.481454 0.564050
H -3.108643 3.027649 1.555388
H -4.208711 4.040513 0.590668
H -2.445361 4.198573 0.396582
C 1.315932 3.537245 -1.338453
C 0.846942 3.871158 -2.770485
H 1.052306 3.041213 -3.461824
H 1.385515 4.758420 -3.138888
H -0.229219 4.083503 -2.818778
C 0.992835 4.734351 -0.425170
H -0.073061 4.998567 -0.430403
H 1.556193 5.617262 -0.765152
H 1.284363 4.534807 0.615692
C 2.842969 3.407158 -1.383156
H 3.288719 3.302711 -0.387915
H 3.252654 4.323061 -1.836688
H 3.182703 2.558864 -1.987048
C -2.827848 -0.610897 0.051427
C -3.208820 -0.905178 1.381651
C -4.140424 -1.925841 1.600153
H -4.471701 -2.139952 2.617554
C -4.659216 -2.667925 0.543858
H -5.381232 -3.465152 0.734799
C -4.250056 -2.391795 -0.757431
H -4.653471 -2.983833 -1.580754
C -3.340344 -1.366301 -1.030176
C -2.637157 -0.117609 2.543384
H -2.165634 0.781187 2.115092
C -1.501421 -0.883040 3.250185
H -0.720161 -1.111468 2.501718
H -1.023785 -0.201950 3.971718
C -1.874635 -2.183891 3.942437
H -0.994970 -2.613881 4.445707
H -2.238391 -2.933080 3.223712
H -2.653957 -2.041998 4.708050
C -3.725243 0.377719 3.507657
H -4.517686 0.861049 2.912310
H -4.213249 -0.474748 4.007987
C -3.215791 1.355407 4.557827
H -2.696055 2.207508 4.089522
H -2.507744 0.881650 5.254727
H -4.044139 1.760469 5.158899
C -2.883895 -1.083201 -2.450582
H -2.663579 -0.005237 -2.512987
C -1.559387 -1.807603 -2.764310
H -1.292807 -1.598533 -3.814239
H -0.746819 -1.339949 -2.178065
C -1.547079 -3.304887 -2.503067
H -0.577008 -3.742491 -2.781849
H -2.325197 -3.828186 -3.080005
H -1.712425 -3.522807 -1.437431
C -3.936179 -1.383698 -3.525290
H -3.474093 -1.171280 -4.504383
H -4.166462 -2.462077 -3.544673
C -5.225368 -0.585148 -3.396830
H -5.027698 0.497435 -3.433283
H -5.741216 -0.794344 -2.447623
H -5.923048 -0.822486 -4.214486
C 2.435419 0.788582 -0.325358
C 3.031274 1.200155 0.893727
C 4.330109 0.774274 1.181847
H 4.806873 1.096379 2.108853
C 5.024988 -0.065955 0.316125
H 6.040325 -0.387488 0.558600
C 4.407642 -0.516914 -0.845324
H 4.946725 -1.199861 -1.502453
C 3.109592 -0.117721 -1.182826
C 2.241437 2.029718 1.887986
H 1.540979 2.652019 1.314232

C	3.107693	2.994724	2.705095
H	3.812763	2.437273	3.342945
H	3.735001	3.570526	2.003483
C	2.306096	3.953031	3.575893
H	2.962856	4.690474	4.061809
H	1.760704	3.426164	4.373550
H	1.562688	4.509995	2.982719
C	1.353038	1.121172	2.757168
H	0.671640	1.748509	3.354458
H	0.691894	0.553408	2.081014
C	2.093643	0.143070	3.652208
H	2.739492	-0.523388	3.061679
H	1.387640	-0.491369	4.208252
H	2.726144	0.659133	4.391348
C	2.435122	-0.629481	-2.442650
H	1.359189	-0.726657	-2.205055
C	2.502445	0.395748	-3.586123
H	1.863300	0.048763	-4.414449
H	2.035776	1.323224	-3.227101
C	3.903835	0.689658	-4.099535
H	3.884701	1.472091	-4.873625
H	4.372790	-0.201605	-4.545145
H	4.562975	1.036939	-3.288387
C	2.903709	-2.029795	-2.848676
H	3.979122	-2.025226	-3.089130
H	2.796570	-2.687302	-1.968773
C	2.138543	-2.623146	-4.022518
H	2.338880	-2.086178	-4.962239
H	1.052494	-2.588995	-3.847895
H	2.416793	-3.675892	-4.182171
C	3.593611	-3.918161	0.677096
C	3.593283	-3.213590	1.908729
C	2.564919	-3.756232	2.724171
C	1.927717	-4.795651	1.995410
C	2.563511	-4.895793	0.728360
H	4.276736	-3.746733	-0.154039
H	2.321542	-5.614331	-0.055028
H	1.108836	-5.420280	2.352734
H	2.316781	-3.439177	3.737078
H	4.267721	-2.400391	2.174873

(BDI*)MgCp (**2**)

125

Mg	0.028511	0.367893	-0.969480
N	-1.500517	0.066797	0.413741
N	1.546177	-0.023543	0.410542
C	-1.286594	-0.288087	1.680863
C	0.010350	-0.464249	2.205247
H	0.003532	-0.745971	3.248830
C	1.313015	-0.367940	1.677638
C	-2.394534	-0.577163	2.758374
C	-3.848496	-0.280744	2.368354
H	-4.001151	0.752305	2.040750
H	-4.477257	-0.444107	3.257307
H	-4.222908	-0.934844	1.576713
C	-2.117989	0.255384	4.030342
H	-1.168571	0.000410	4.517848
H	-2.919554	0.069762	4.761440
H	-2.103873	1.333209	3.817095
C	-2.319086	-2.069101	3.138203
H	-2.522987	-2.712915	2.272747
H	-3.075736	-2.293094	3.906569
H	-1.334842	-2.347301	3.538822
C	2.407020	-0.669004	2.766362
C	2.232658	0.341217	3.922505
H	2.362368	1.377205	3.580910
H	2.993341	0.145238	4.694095
H	1.246680	0.272311	4.399482
C	2.199697	-2.084323	3.341105
H	1.214193	-2.213802	3.807673
H	2.962031	-2.279200	4.111305
H	2.308101	-2.854909	2.566275
C	3.873597	-0.582570	2.325433
H	4.151811	-1.365693	1.613181
H	4.504168	-0.708505	3.219273
H	4.130614	0.379935	1.873685
C	-2.766893	0.236846	-0.202603
C	-3.478053	-0.884293	-0.694703
C	-4.687828	-0.665501	-1.361647
H	-5.261643	-1.519355	-1.725163
C	-5.181182	0.619666	-1.562457
H	-6.131966	0.768567	-2.079423
C	-4.449863	1.715611	-1.114953
H	-4.835259	2.719706	-1.295378
C	-3.232931	1.553100	-0.444919
C	-2.948661	-2.295020	-0.509603
H	-2.226550	-2.259781	0.318448
C	-2.132178	-2.759912	-1.729215
H	-1.335521	-2.023148	-1.896548
H	-1.613955	-3.695769	-1.465096
C	-2.906079	-2.953662	-3.023373
H	-3.401528	-2.025127	-3.343879
H	-3.682213	-3.729833	-2.929738
H	-2.231148	-3.262750	-3.836169
C	-4.041769	-3.294192	-0.104424
H	-4.584036	-2.883791	0.763642
H	-4.795208	-3.384363	-0.903402
C	-3.514412	-4.681733	0.236325
H	-4.317166	-5.324992	0.627839
H	-2.722792	-4.635689	1.002029
H	-3.089336	-5.188455	-0.643248
C	-2.420145	2.759365	-0.002546

H	-1.361571	2.506985	-0.199905
C	-2.726488	4.026622	-0.809045
H	-2.769476	3.770540	-1.879556
H	-3.733885	4.398160	-0.559921
C	-1.713076	5.141795	-0.599191
H	-1.928116	6.002361	-1.250369
H	-0.693246	4.796337	-0.830973
H	-1.707969	5.508845	0.438363
C	-2.504685	3.007183	1.512982
H	-2.209832	2.080203	2.018354
H	-1.736834	3.745171	1.797416
C	-3.867129	3.463466	2.013967
H	-4.113657	4.480067	1.671152
H	-3.899635	3.473372	3.114262
H	-4.669935	2.795695	1.662809
C	2.794629	-0.090483	-0.257140
C	3.438761	1.108215	-0.649065
C	4.611433	1.016352	-1.406774
H	5.131159	1.927047	-1.705736
C	5.126174	-0.214888	-1.799462
H	6.040731	-0.262755	-2.395007
C	4.464481	-1.384615	-1.437610
H	4.868900	-2.346343	-1.756600
C	3.298032	-1.349682	-0.668844
C	2.861471	2.461115	-0.270084
H	1.765245	2.367890	-0.373761
C	3.301967	3.589375	-1.209408
H	4.375848	3.797063	-1.072974
H	3.202908	3.243560	-2.250742
C	2.518039	4.880606	-1.025603
H	1.435868	4.707203	-1.136473
H	2.811550	5.633329	-1.772614
H	2.677576	5.325190	-0.031567
C	3.101989	2.806797	1.208270
H	2.494529	3.687710	1.473337
H	2.692796	1.984910	1.807572
C	4.555966	3.051487	1.583595
H	4.670240	3.153943	2.673660
H	4.950546	3.971657	1.125795
H	5.201264	2.220421	1.256959
C	2.582151	-2.632237	-0.284895
H	2.058952	-2.436017	0.660165
C	1.467993	-2.981836	-1.283539
H	0.851458	-3.789019	-0.856841
H	0.793845	-2.115008	-1.353955
C	1.929642	-3.363606	-2.679303
H	1.069695	-3.586305	-3.329300
H	2.568083	-4.261007	-2.668878
H	2.502831	-2.550373	-3.149285
C	3.538961	-3.800306	-0.015149
H	4.093465	-4.065745	-0.929665
H	4.303363	-3.460600	0.704147
C	2.852249	-5.048949	0.522434
H	2.180181	-5.502231	-0.221964
H	2.247272	-4.824952	1.415994
H	3.590082	-5.815005	0.805403
C	-0.746184	1.727557	-2.802699
H	-1.420402	2.566181	-2.654731
C	-1.131813	0.397562	-3.088797
H	-2.156262	0.037440	-3.163775
C	0.051699	-0.369916	-3.259506
H	0.097410	-1.421202	-3.535574
C	1.169436	0.487291	-3.079218
H	2.218096	0.202614	-3.151551
C	0.673598	1.785383	-2.795640
H	1.269891	2.682020	-2.651310

Mg^0

1

Mg	0.000000	0.000000	0.000000

(BDI*)MgCp*

140

Mg	-0.012182	-0.388138	-0.634193
N	1.704021	0.129680	0.493384
N	-1.307263	0.283000	0.848425
C	1.650332	0.678521	1.706227
C	0.435211	0.933905	2.365949
H	0.576219	1.363311	3.346579
C	-0.920308	0.799984	2.017794
C	2.879540	1.092690	2.586061
C	4.266990	0.988975	1.947291
H	4.511333	-0.016849	1.606919
H	5.012437	1.271802	2.706705
H	4.391119	1.667753	1.098871
C	2.883895	0.190479	3.836561
H	1.966900	0.294724	4.431626
H	3.739583	0.449552	4.479765
H	2.980587	-0.869407	3.559723
C	2.725945	2.564257	3.023114
H	2.617416	3.224885	2.150470
H	3.627473	2.878036	3.571496
H	1.865005	2.736228	3.681642
C	-1.857368	1.381608	3.144109
C	-1.489785	0.769427	4.515343
H	-1.603293	-0.322927	4.523639
H	-2.167247	1.180154	5.279214
H	-0.465948	1.000376	4.835559
C	-1.600613	2.899628	3.226604
H	-0.559802	3.131149	3.489699
H	-2.253775	3.343507	3.994306
H	-1.821397	3.394526	2.273904

C	-3.371856	1.185165	2.990694
H	-3.793841	1.727728	2.141565
H	-3.855109	1.570147	3.901986
H	-3.659075	0.135704	2.883512
C	2.882623	-0.088789	-0.268311
C	3.347418	0.942417	-1.122499
C	4.362297	0.636163	-2.035857
H	4.732718	1.412165	-2.708479
C	4.940184	-0.631148	-2.073321
H	5.725991	-0.852766	-2.798983
C	4.550767	-1.597084	-1.148693
H	5.061733	-2.561898	-1.136090
C	3.529674	-1.348816	-0.226914
C	2.859366	2.370760	-0.923265
H	2.442045	2.402239	0.093223
C	1.698895	2.795411	-1.839052
H	0.950216	1.987385	-1.852964
H	1.185139	3.651505	-1.372207
C	2.090959	3.163337	-3.261522
H	1.202087	3.376365	-3.872489
H	2.643832	2.351370	-3.757417
H	2.726728	4.061285	-3.285784
C	4.020212	3.377407	-0.949183
H	4.831906	2.996198	-0.308422
H	4.453714	3.434006	-1.960421
C	3.628157	4.774986	-0.489697
H	3.186330	4.754081	0.519457
H	2.890069	5.240377	-1.160569
H	4.503896	5.440682	-0.453818
C	3.167380	-2.360835	0.849698
H	2.742543	-1.775644	1.678456
C	2.052663	-3.338700	0.416849
H	1.542414	-3.705219	1.324223
H	1.289164	-2.778578	-0.149814
C	2.524221	-4.532992	-0.403132
H	1.671623	-5.114829	-0.776251
H	3.147140	-5.215260	0.192919
H	3.117039	-4.221174	-1.276842
C	4.368343	-3.142803	1.405401
H	3.971728	-3.866809	2.138719
H	4.808658	-3.759407	0.605942
C	5.475805	-2.331182	2.059661
H	5.108975	-1.757615	2.923269
H	5.930593	-1.620010	1.353681
H	6.276976	-2.994272	2.421007
C	-2.660644	0.168320	0.434534
C	-3.320213	1.280144	-0.144137
C	-4.656594	1.126545	-0.528168
H	-5.198135	1.978715	-0.942919
C	-5.317425	-0.086831	-0.362565
H	-6.365937	-0.183249	-0.653640
C	-4.636501	-1.185153	0.157661
H	-5.163055	-2.134282	0.263255
C	-3.295568	-1.093476	0.544003
C	-2.610481	2.615805	-0.293408
H	-1.725946	2.569011	0.357139
C	-3.458050	3.801426	0.189630
H	-4.349913	3.915846	-0.447140
H	-3.846733	3.573749	1.195121
C	-2.696823	5.120419	0.228792
H	-3.316388	5.924277	0.654875
H	-2.381370	5.444858	-0.774130
H	-1.786445	5.041447	0.844874
C	-2.052497	2.832086	-1.716971
H	-1.215021	3.545441	-1.655001
H	-1.610099	1.881304	-2.053924
C	-3.047659	3.335186	-2.752844
H	-2.585293	3.368161	-3.751079
H	-3.396770	4.353751	-2.524699
H	-3.932025	2.687142	-2.826082
C	-2.542481	-2.294182	1.106889
H	-1.513352	-2.232520	0.704791
C	-2.400002	-2.231703	2.636389
H	-1.652453	-2.976613	2.953700
H	-1.962811	-1.262844	2.897322
C	-3.696304	-2.447189	3.404141
H	-3.560137	-2.240701	4.476900
H	-4.061012	-3.481691	3.313390
H	-4.498291	-1.786771	3.037936
C	-3.100607	-3.645302	0.652220
H	-4.152147	-3.748540	0.965916
H	-3.110760	-3.660137	-0.443348
C	-2.320254	-4.844504	1.175814
H	-2.467098	-4.999972	2.255370
H	-1.238517	-4.728075	1.005703
H	-2.634840	-5.768162	0.666811
C	-2.099579	-2.075475	-2.636613
C	-1.874812	-0.748573	-3.079246
C	-0.486737	-0.476996	-3.016935
C	0.172632	-1.674578	-2.501350
C	-0.862119	-2.649625	-2.277435
C	-3.436171	-2.742453	-2.718721
H	-3.380130	-3.819742	-2.507305
H	-3.854471	-2.638713	-3.734712
H	-4.173481	-2.301829	-2.030800
C	-2.939199	0.117989	-3.662983
H	-3.683746	0.425873	-2.912472
H	-3.491238	-0.423808	-4.450160
H	-2.526523	1.023647	-4.124382
C	0.216033	0.528131	-3.879295
H	-0.257832	1.519919	-3.857745
H	0.216483	0.194136	-4.933161

H	1.266210	0.654352	-3.590540
C	1.581018	-2.023160	-2.900543
H	2.277849	-1.180369	-2.800099
H	1.608393	-2.341428	-3.959851
H	1.993946	-2.844745	-2.306650
C	-0.632881	-4.079282	-1.907593
H	-0.358351	-4.217771	-0.851372
H	0.176960	-4.524015	-2.508886
H	-1.534667	-4.683374	-2.079948

$NaPPh_2$

24

P	-0.002764	-1.408789	-0.872189
C	1.370851	-0.264355	-0.456405
C	-1.480409	-0.398229	-0.432128
C	-2.534643	-0.980160	0.305327
H	-2.431740	-2.018097	0.643321
C	1.292220	0.929910	0.311047
H	0.311116	1.337142	0.577455
C	-1.674857	0.925519	-0.883523
H	-0.901509	1.395812	-1.496387
C	2.679689	-0.685741	-0.813263
H	2.788226	-1.588008	-1.423436
C	-3.856848	1.042939	0.167881
H	-4.766748	1.600798	0.401723
C	-3.704956	-0.276489	0.596360
H	-4.501771	-0.762688	1.166384
C	2.436750	1.620661	0.719017
H	2.324157	2.537364	1.305955
C	-2.833230	1.636229	-0.577424
H	-2.946816	2.662274	-0.938757
C	3.813622	0.005791	-0.405506
H	4.799693	-0.359953	-0.706412
C	3.709836	1.164997	0.375245
H	4.602979	1.709216	0.689470
Na	0.495974	-1.246224	1.765795

(BDI*)MgMgPPh_2

139

P	-3.997036	-2.692446	1.559118
Mg	0.219419	-0.051380	0.010745
N	2.257465	-0.401530	0.051074
N	0.408545	1.965487	-0.341591
C	2.478873	-1.796492	0.008393
C	2.102884	-2.598330	1.115588
C	3.161660	0.565773	-0.060556
C	2.886858	-2.405923	-1.206236
C	1.577861	2.572851	-0.520995
C	-0.832535	2.555791	-0.001192
C	2.234580	-3.987175	1.013285
H	1.968681	-4.612140	1.866673
C	-1.011784	3.052950	1.314627
C	2.808668	1.895441	-0.389258
H	3.666855	2.542816	-0.523401
C	-1.925895	2.478030	-0.895747
C	1.727187	4.057938	-0.967703
C	4.682140	0.394640	0.248988
C	1.566085	-1.977322	2.391742
H	1.082951	-1.025858	2.106617
C	5.149486	-1.027269	0.580381
H	4.579572	-1.482060	1.397106
H	6.202552	-0.977016	0.897770
H	5.092534	-1.705338	-0.277638
C	2.985472	-3.798919	-1.259353
H	3.301031	-4.276193	-2.189706
C	-2.293441	0.599960	-2.587767
H	-1.955780	0.281622	-3.586950
H	-1.822021	-0.104920	-1.878563
C	2.685235	-4.590400	-0.155606
H	2.780920	-5.677075	-0.213750
C	0.149212	3.098233	2.290497
H	1.076664	3.078193	1.701459
C	3.157394	-1.598630	-2.464305
H	3.270411	-0.541257	-2.180391
C	-1.726028	2.007899	-2.323084
H	-0.635945	1.927992	-2.469338
C	4.967750	1.262427	1.494253
H	4.760663	2.325818	1.315332
H	6.025169	1.160993	1.785425
H	4.349123	0.942749	2.346171
C	-3.371661	3.395747	0.830725
H	-4.360532	3.728388	1.154324
C	-3.183987	2.907488	-0.457689
H	-4.031719	2.869843	-1.142829
C	2.675977	-1.574551	3.377287
H	3.305108	-0.818253	2.884037
H	2.211370	-1.053793	4.230763
C	1.950634	-1.674238	-3.410818
H	2.189335	-1.134797	-4.341407
H	1.786489	-2.728203	-3.696517
C	0.199395	1.827100	3.160152
H	0.212103	0.952121	2.484855
H	1.165089	1.798839	3.690621
C	-2.288295	3.470672	1.702723
H	-2.439449	3.865790	2.708760
C	0.458313	-2.831746	3.019949
H	-0.212329	-3.153918	2.204578
H	0.872401	-3.761985	3.441341
C	-2.974514	-3.413106	0.194213
C	0.428367	4.866893	-1.024653
H	-0.322736	4.404186	-1.673289
H	0.658625	5.860876	-1.438758

H	-0.027750	5.011987	-0.038460
C	5.546869	0.893252	-0.920966
H	5.336542	0.328631	-1.838775
H	6.610958	0.749988	-0.677197
H	5.401606	1.959694	-1.139690
C	-2.228237	3.052786	-3.333574
H	-3.322245	3.161629	-3.254062
H	-1.817983	4.034591	-3.046091
C	-5.108993	-1.538001	0.641109
C	-5.238856	-0.200669	1.063589
H	-4.624043	0.164279	1.893532
C	3.546840	-2.715769	3.880847
H	2.957346	-3.473217	4.420892
H	4.318053	-2.345870	4.573784
H	4.059821	-3.227243	3.051659
C	-2.929155	-2.962238	-1.145670
H	-3.658815	-2.227553	-1.490069
C	-5.927719	-1.965049	-0.422326
H	-5.871964	-3.004998	-0.754433
C	0.192178	4.398676	3.103558
H	0.143229	5.243153	2.395476
H	-0.705973	4.494920	3.734915
C	2.294298	4.015422	-2.401911
H	3.272601	3.517432	-2.446473
H	2.409833	5.039375	-2.791464
H	1.609558	3.472915	-3.072228
C	-2.000565	-4.367792	0.577482
H	-2.011344	-4.749949	1.603016
C	2.694731	4.822518	-0.048313
H	2.341154	4.812466	0.992832
H	2.755328	5.873492	-0.370755
H	3.715324	4.417116	-0.062186
C	-6.920138	0.236544	-0.618924
H	-7.614120	0.921385	-1.111943
C	-0.354892	-2.114715	4.085371
H	-1.177698	-2.747498	4.449327
H	-0.806100	-1.193750	3.680107
H	0.254563	-1.823746	4.954401
C	0.671808	-1.099929	-2.827720
H	-0.155130	-1.143818	-3.548038
H	0.810408	-0.039789	-2.554552
H	0.342403	-1.675951	-1.944107
C	-6.136451	0.673195	0.448696
H	-6.208260	1.705628	0.798792
C	-1.958598	-3.426589	-2.036780
H	-1.958190	-3.050188	-3.063046
C	-3.801097	0.438175	-2.484182
H	-4.111164	-0.554139	-2.842707
H	-4.344855	1.178776	-3.091016
H	-4.155349	0.529555	-1.448151
C	-0.943386	1.661696	4.148827
H	-1.907788	1.542658	3.631168
H	-1.032373	2.527621	4.823250
H	-0.792918	0.773992	4.779399
C	4.457739	-2.020934	-3.166103
H	5.259799	-2.073439	-2.411573
H	4.353596	-3.046370	-3.558640
C	-6.808746	-1.087971	-1.051204
H	-7.424890	-1.444552	-1.881052
C	-1.019265	-4.804256	-0.307253
H	-0.266924	-5.518723	0.033354
C	4.890764	-1.097880	-4.298495
H	4.184171	-1.114176	-5.141710
H	5.874439	-1.394235	-4.693457
H	4.970707	-0.052575	-3.959045
C	1.432364	4.535227	3.976641
H	1.451400	3.787510	4.784145
H	1.478193	5.528125	4.449391
H	2.354433	4.403458	3.387017
C	-0.986953	-4.336783	-1.624993
H	-0.209073	-4.675698	-2.312013
C	-1.849453	2.751304	-4.776828
H	-2.338064	1.838850	-5.151167
H	-0.760929	2.609685	-4.880916
H	-2.142654	3.575871	-5.444511
Mg	-2.048454	-1.176241	1.052114

(BDI*)MgPPh$_2$ (**4**)
138

P	0.561064	2.243452	1.414356
Mg	0.014569	0.247718	0.016161
N	-1.861884	-0.608515	0.078974
N	0.949375	-1.379818	-0.787885
C	-2.823373	0.359457	0.458338
C	-2.902028	0.773952	1.810667
C	-2.114337	-1.815889	-0.412867
C	-3.583224	1.012731	-0.544703
C	0.263736	-2.426331	-1.260399
C	2.346962	-1.403099	-0.543718
C	-3.833577	1.761324	2.149467
H	-3.927007	2.071685	3.190694
C	2.820397	-2.123629	0.579994
C	-1.122982	-2.574953	-1.077086
H	-1.506898	-3.499065	-1.490377
C	3.230110	-0.651875	-1.348904
C	0.887801	-3.594397	-2.091667
C	-3.469791	-2.584227	-0.278886
C	-2.000339	0.170283	2.871318
H	-1.038003	-0.045715	2.378366
C	-4.614240	-1.840228	0.418643
H	-4.338252	-1.468809	1.409622
H	-5.449770	-2.545208	0.549223

H	-4.985683	-0.989170	-0.161554
C	-4.477241	2.014123	-0.153836
H	-5.072523	2.525173	-0.912641
C	2.511537	1.646237	-2.079451
H	1.904127	2.159245	-2.841526
H	1.905792	1.675981	-1.154658
C	-4.626793	2.371394	1.182168
H	-5.343052	3.145089	1.467703
C	1.843427	-2.799255	1.522299
H	0.933153	-3.027522	0.950162
C	-3.454590	0.658791	-2.019329
H	-3.011188	-0.345822	-2.091691
C	2.696472	0.173928	-2.499576
H	1.687682	-0.214580	-2.716130
C	-3.184132	-3.851942	0.556499
H	-2.452585	-4.513751	0.074712
H	-4.117433	-4.419314	0.696709
H	-2.793660	-3.594106	1.551521
C	5.084668	-1.460692	-0.003659
H	6.158601	-1.500744	0.192820
C	4.598256	-0.707599	-1.067831
H	5.298717	-0.160555	-1.700304
C	-2.509741	-1.189936	3.377291
H	-2.553136	-1.873489	2.517397
H	-1.750211	-1.618205	4.051757
C	-2.496867	1.612384	-2.749210
H	-2.559086	1.414934	-3.830962
H	-2.833414	2.651614	-2.599012
C	1.400998	-1.812888	2.624698
H	1.077564	-0.873221	2.140667
H	0.495575	-2.204880	3.114803
C	4.197793	-2.147322	0.822267
H	4.585143	-2.716609	1.668792
C	-1.684346	1.152706	4.005845
H	-1.506220	2.144070	3.560831
H	-2.554155	1.255804	4.676168
C	-0.082491	3.533640	0.278681
C	2.386866	-3.505694	-2.401350
H	2.647727	-2.576096	-2.918479
H	2.641614	-4.339397	-3.074198
H	3.021945	-3.590441	-1.512773
C	-3.992581	-3.013366	-1.661169
H	-4.183605	-2.143172	-2.301904
H	-4.944898	-3.551931	-1.538682
H	-3.303329	-3.681455	-2.194772
C	3.514206	0.012361	-3.787770
H	4.531822	0.411636	-3.649110
H	3.646056	-1.064781	-3.983231
C	2.368873	2.510994	1.458308
C	3.259107	1.425044	1.510196
H	2.886597	0.407396	1.382321
C	-3.857137	-1.158195	4.083571
H	-3.816416	-0.575025	5.016182
H	-4.188284	-2.174557	4.346616
H	-4.636434	-0.706695	3.449602
C	0.676725	4.228110	-0.680161
H	1.753037	4.065739	-0.734874
C	2.906569	3.804351	1.605298
H	2.237309	4.668211	1.602269
C	2.349081	-4.135770	2.074785
H	2.713392	-4.742305	1.228399
H	3.225889	-3.982787	2.724504
C	0.175114	-3.596440	-3.461868
H	-0.905266	-3.774285	-3.382952
H	0.602008	-4.385727	-4.100238
H	0.319672	-2.632291	-3.973700
C	-1.467883	3.782347	0.321126
H	-2.081875	3.266379	1.061753
C	0.652075	-4.936339	-1.377364
H	1.145227	-4.949419	-0.395269
H	1.078648	-5.755312	-1.977320
H	-0.412724	-5.156436	-1.222245
C	5.146355	2.908208	1.819815
H	6.219717	3.062338	1.954148
C	-0.452569	0.768318	4.808913
H	-0.284542	1.472082	5.638053
H	0.438729	0.802729	4.163694
H	-0.526541	-0.241535	5.242227
C	-1.044618	1.495063	-2.321483
H	-0.391946	2.144376	-2.918892
H	-0.681326	0.458389	-2.430096
H	-0.932722	1.857455	-1.285377
C	4.626207	1.619308	1.703168
H	5.286286	0.750369	1.737494
C	0.072153	5.109541	-1.574227
H	0.687930	5.630196	-2.312999
C	3.793731	2.426079	-1.836583
H	3.571160	3.462700	-1.546020
H	4.425843	2.468853	-2.737456
H	4.383177	1.991080	-1.018085
C	2.454959	-1.482990	3.669244
H	3.371262	-1.084316	3.209854
H	2.731758	-2.369139	4.260993
H	2.084136	-0.722048	4.369641
C	-4.822004	0.595706	-2.720398
H	-5.513874	0.018805	-2.084473
H	-5.253449	1.608500	-2.786289
C	4.276336	4.000064	1.765269
H	4.666844	5.016550	1.864592
C	-2.072590	4.654273	-0.581746
H	-3.152556	4.812237	-0.527121
C	-4.790892	-0.017201	-4.115188

H	-4.216207	0.594403	-4.826595
H	-5.808631	-0.116965	-4.522193
H	-4.337606	-1.021323	-4.107134
C	1.284077	-4.910089	2.839440
H	0.968497	-4.381097	3.751798
H	1.652354	-5.900928	3.145927
H	0.384451	-5.063009	2.221048
C	-1.308081	5.323456	-1.539376
H	-1.779825	6.011625	-2.244973
C	2.873912	0.673495	-5.000474
H	2.825497	1.768021	-4.894502
H	1.843723	0.311557	-5.153083
H	3.442101	0.458038	-5.918234

(BDI*)MgMgOAr·THF (**5**)
201

C	4.947329	-5.045032	-1.350532
C	4.280650	-4.828092	0.001165
C	6.862448	-1.861187	0.778636
C	5.580277	-2.038016	2.879273
C	2.487020	-4.221606	2.314887
C	5.906675	-1.096293	1.712662
C	3.487467	-3.519921	0.086757
C	6.646370	0.111343	2.326138
C	1.941773	-3.924878	3.563286
C	2.969759	-3.212421	1.477200
C	1.300619	-4.544296	-0.855951
C	2.335290	-3.433787	-0.935560
C	1.817955	-2.596749	3.958712
C	3.350189	-0.375602	5.822216
C	2.933373	-1.870775	1.936445
C	4.699517	-0.544567	0.882125
C	2.284710	-1.551422	3.151314
C	6.293812	0.852921	-2.781816
C	5.107323	0.340706	-0.136515
C	3.187272	0.395607	4.520714
C	2.087731	-0.109950	3.568924
C	2.950260	-0.337689	-4.179146
C	0.676637	-2.306972	-4.372082
C	4.418317	1.093158	-1.116844
C	6.274917	2.822415	-1.246805
C	5.381135	1.873768	-2.069581
C	2.904204	0.308459	-5.555495
C	-0.988411	-2.684075	1.910331
C	-1.882513	-3.382158	2.928051
C	0.663386	0.142203	4.083746
C	-2.770050	-4.032368	-1.409570
C	-3.910480	-4.949805	-1.855525
C	1.663839	-0.178330	-3.353439
C	-2.040125	-3.476200	-2.633946
C	0.452257	-0.845870	-4.010415
C	4.701738	2.710228	-3.159269
C	-2.565797	-2.219148	3.649089
C	-3.338596	-2.863523	-0.606673
C	0.280025	1.613336	4.107905
C	3.341369	3.061714	1.972598
C	2.221213	1.886518	-1.964744
C	-4.889524	-4.144995	-2.716079
C	1.427490	1.272734	-2.967289
C	-3.024691	-2.699934	-3.516407
C	-4.159506	-3.626544	-3.958039
C	-2.752477	-1.217127	2.524980
C	2.220861	3.285989	0.969981
C	2.736494	3.805369	-0.390179
C	-4.317804	-2.018179	-1.444496
C	-5.443492	-2.970847	-1.898366
C	1.965010	3.220831	-1.557644
C	0.438431	2.040300	-3.592601
C	-3.603824	-1.526397	-2.724508
C	-4.830279	-0.835196	-0.621583
C	2.813505	5.334826	-0.369537
C	3.558674	5.964146	-1.538095
C	0.973268	3.943760	-2.221497
C	0.217726	3.366686	-3.237751
C	-3.970500	0.248816	-0.268810
C	-6.163998	-0.808207	-0.197203
C	-2.587335	2.508409	1.593777
C	-8.907255	-1.021235	0.660159
C	-4.539824	1.391769	0.377009
C	-6.710229	0.245592	0.532967
C	-1.761301	3.790956	1.719200
C	-1.183776	4.152634	0.348527
C	-8.166403	0.400577	2.561813
C	-5.875495	1.340510	0.775876
C	-3.729239	2.676118	0.566999
C	-8.158132	0.262045	1.030105
C	-3.142321	3.125203	-0.793085
C	-2.319727	4.404933	-0.644436
C	-2.655347	4.926405	2.221653
C	-4.600241	3.842552	1.083745
C	-8.904167	1.453154	0.405620
C	-3.795299	5.139347	1.222795
C	-3.211903	5.538503	-0.135062
H	5.583067	-5.943361	-1.341640
H	5.047156	-4.817364	0.794540
H	7.716365	-2.247089	1.357302
H	5.583988	-4.187764	-1.623784
H	3.633593	-5.691102	0.228061
H	4.208953	-5.174389	-2.156643
H	6.518369	-2.249292	3.415730
H	6.356797	-2.720729	0.315804
H	5.163679	-2.996853	2.553345

H	2.543625	-5.261812	1.989693
H	7.486905	-0.242863	2.943137
H	7.260904	-1.232421	-0.028674
H	1.587069	-4.728428	4.212936
H	4.881140	-1.599172	3.598573
H	1.732788	-5.528886	-1.092452
H	4.182699	-2.713938	-0.187946
H	7.050636	0.794319	1.568098
H	3.643750	-1.421015	5.642942
H	2.761282	-3.388167	-1.950836
H	5.974929	0.695292	2.973231
H	4.127766	0.084279	6.451125
H	0.858402	-4.607551	0.149565
H	6.903034	0.272042	-2.076850
H	1.337684	-2.362457	4.910249
H	6.181667	0.464721	-0.186646
H	0.482262	-4.366094	-1.568106
H	2.419847	-0.389156	6.411715
H	6.978430	1.384959	-3.460725
H	5.711496	0.140417	-3.382569
H	4.140387	0.392440	3.970136
H	1.816373	-2.466831	-0.793167
H	3.187740	-1.409596	-4.273360
H	6.927573	2.284616	-0.546105
H	1.447489	-2.423885	-5.148504
H	0.994346	-2.894947	-3.498105
H	3.882971	0.245398	-6.055442
H	-1.300349	-4.028816	3.599025
H	3.773285	0.089234	-3.593860
H	0.040434	-2.548309	2.265751
H	-2.060024	-4.599570	-0.782332
H	2.987528	1.455047	4.748379
H	-0.959651	-3.202492	0.941427
H	6.920189	3.399597	-1.927226
H	-3.513848	-5.806170	-2.427512
H	0.528263	-0.297560	5.086273
H	-0.245761	-2.761194	-4.759478
H	2.167117	-0.177508	-6.213517
H	-1.573061	-4.295290	-3.206671
H	2.196321	0.494239	2.652638
H	1.849376	-0.732804	-2.412954
H	-4.429430	-5.363371	-0.973789
H	-2.631791	-4.006675	2.418221
H	5.680802	3.539119	-0.662509
H	-0.031545	-0.390565	3.417014
H	-1.225866	-2.808161	-2.305639
H	2.629902	1.373200	-5.487881
H	5.483490	3.130808	-3.810537
H	4.000590	2.242199	1.645725
H	-1.904425	-1.802586	4.425032
H	4.028261	2.120998	-3.789877

H	0.163793	-0.301095	-4.923581
H	-3.865929	-3.232534	0.289537
H	-3.765341	-4.472505	-4.547635
H	-2.526081	-2.216984	-0.258029
H	0.906847	2.201852	4.795169
H	-0.407754	-0.754637	-3.324929
H	2.950930	2.809917	2.967851
H	-3.517053	-2.499502	4.122711
H	4.126028	3.546954	-2.753987
H	-5.728758	-4.792031	-3.022368
H	3.963682	3.964150	2.083652
H	3.767451	3.437103	-0.481274
H	0.382262	2.051960	3.103389
H	-2.496205	-2.310544	-4.402849
H	-0.766238	1.749290	4.420741
H	1.645703	2.351615	0.831388
H	-5.975189	-3.374262	-1.021007
H	-2.800970	-0.169207	2.847261
H	-3.639949	-1.427813	1.907069
H	-4.859912	-3.077473	-4.609588
H	-0.179622	1.591722	-4.370222
H	-2.814220	-0.816118	-2.448609
H	4.606100	5.626789	-1.571110
H	1.481274	3.998286	1.368713
H	-6.184796	-2.412845	-2.494120
H	3.306723	5.630016	0.572058
H	-4.331425	-0.972281	-3.340968
H	3.098584	5.703952	-2.503336
H	-6.802219	-1.650201	-0.452251
H	-1.922787	1.682582	1.296355
H	-8.426082	-1.911746	1.093157
H	3.567466	7.062046	-1.458662
H	1.793726	5.750609	-0.299793
H	0.770569	4.973842	-1.925727
H	-0.559316	3.945682	-3.741229
H	-0.543797	3.335514	-0.021619
H	-3.018810	2.229760	2.570395
H	-7.641465	-0.446141	3.031156
H	-0.939117	3.624903	2.433086
H	-8.962591	-1.160274	-0.430301
H	-2.523672	2.319735	-1.209686
H	-9.938882	-0.979220	1.043144
H	-0.542760	5.046843	0.433645
H	-9.198218	0.423976	2.949985
H	-7.664645	1.324619	2.885934
H	-6.297756	2.200324	1.294464
H	-3.978365	3.285203	-1.494396
H	-3.061378	4.676819	3.216997
H	-1.894782	4.674238	-1.624772
H	-5.032063	3.584295	2.065014
H	-2.067579	5.853409	2.336260

H	-8.906511	1.376787	-0.692589
H	-9.950454	1.487208	0.751931
H	-5.444639	4.002159	0.393467
H	-8.432129	2.410736	0.671315
H	-2.634639	6.474778	-0.040227
H	-4.024584	5.731890	-0.855562
H	-4.465238	5.935687	1.588853
Mg	1.831524	-0.002952	0.046292
Mg	-0.954667	-0.167446	0.130339
N	3.418192	-0.837857	1.104263
N	3.096318	1.081110	-1.200219
O	-1.567353	-1.374484	1.724850
O	-2.679594	0.208810	-0.537015

(BDI*)MgMgOAr
188

C	5.353480	-4.353507	-2.542580
C	4.376194	-4.585869	-1.398052
C	6.691386	-2.144372	0.783976
C	5.011882	-3.004492	2.377220
C	2.036352	-4.719140	0.456201
C	5.512983	-1.732798	1.683436
C	3.588557	-3.333300	-1.001178
C	6.024677	-0.786929	2.790528
C	1.138188	-4.848406	1.513912
C	2.719661	-3.523408	0.226793
C	1.750325	-3.698783	-2.784557
C	2.750082	-2.742654	-2.155073
C	0.887603	-3.758453	2.339271
C	2.078763	-2.596289	5.200069
C	2.524130	-2.447220	1.131173
C	4.445318	-0.926953	0.880965
C	1.561017	-2.543906	2.162522
C	6.167034	1.148863	-2.304998
C	4.971025	0.195426	0.207813
C	2.155700	-1.352248	4.327533
C	1.268557	-1.364940	3.068256
C	2.482327	0.917972	-4.100853
C	-0.295777	-0.652379	-3.809493
C	4.391211	1.231227	-0.562997
C	6.480469	2.671737	-0.334868
C	5.447324	2.093715	-1.319115
C	2.457326	1.970062	-5.199492
C	-0.227478	-1.213595	3.377505
C	-2.399053	-4.133862	0.521660
C	-3.333956	-5.314519	0.247754
C	1.408871	1.078174	-3.011085
C	-1.459913	-3.931057	-0.670845
C	-0.021690	0.810379	-3.498458
C	4.892915	3.261920	-2.141731
C	-3.232503	-2.870494	0.732378
C	-0.614391	0.191973	3.813644
C	2.983773	2.112292	3.060325
C	2.355604	2.510468	-1.126279
C	-4.160789	-5.016022	-1.006646
C	1.520624	2.389628	-2.260500
C	-2.280283	-3.668609	-1.938738
C	-3.214710	-4.853079	-2.200370
C	2.142933	2.860032	2.038128
C	2.984498	3.676220	1.030406
C	-4.055357	-2.521053	-0.530412
C	-4.973925	-3.730248	-0.800194
C	2.303044	3.679675	-0.323148
C	0.779743	3.506220	-2.668217
C	-3.119435	-2.403014	-1.756481
C	-4.826210	-1.221507	-0.303400
C	3.304057	5.060941	1.598632
C	4.300525	5.876512	0.786725
C	1.543859	4.762552	-0.765300
C	0.814711	4.696003	-1.950898
C	-4.125417	-0.014931	-0.041912
C	-6.224865	-1.191649	-0.320770
C	-3.211541	2.314144	1.779047
C	-9.096815	-1.361420	-0.421066
C	-4.846828	1.185257	0.218230
C	-6.959895	-0.029210	-0.082521
C	-2.384924	3.571573	2.050669
C	-1.512829	3.888476	0.830399
C	-9.008170	0.454075	1.276640
C	-6.239839	1.140645	0.188979
C	-4.093533	2.480136	0.518550
C	-8.491111	0.008296	-0.101498
C	-3.219864	2.886247	-0.692009
C	-2.402739	4.142203	-0.390065
C	-3.335602	4.740892	2.322841
C	-5.029152	3.673129	0.801290
C	-8.964363	1.005367	-1.172562
C	-4.231916	4.951473	1.098183
C	-3.353724	5.306998	-0.105207
H	5.969747	-5.246490	-2.727006
H	4.924383	-4.940801	-0.508816
H	7.417554	-2.724371	1.374206
H	6.036302	-3.516367	-2.323694
H	3.692676	-5.406651	-1.669029
H	4.834550	-4.113915	-3.483277
H	5.824745	-3.394211	3.009341
H	6.354776	-2.779840	-0.047363
H	4.733019	-3.792968	1.669155
H	2.195751	-5.565268	-0.214217
H	6.783934	-1.301379	3.400393
H	7.227275	-1.285809	0.357324
H	0.610460	-5.791075	1.673608

H	4.147685	-2.821325	3.024731
H	2.247985	-4.570669	-3.236282
H	4.336076	-2.563848	-0.765729
H	6.474513	0.127445	2.380740
H	2.411577	-3.493202	4.656170
H	3.431129	-2.344317	-2.924921
H	5.203316	-0.484679	3.457400
H	2.717698	-2.487560	6.089728
H	1.030396	-4.069596	-2.041327
H	6.672000	0.319455	-1.792149
H	0.153573	-3.850281	3.141508
H	6.050794	0.260333	0.265188
H	1.177983	-3.202436	-3.580540
H	1.053925	-2.787174	5.555225
H	6.921664	1.711656	-2.876716
H	5.455605	0.712724	-3.021978
H	3.196824	-1.192372	4.006510
H	2.208896	-1.861336	-1.765886
H	2.386261	-0.087806	-4.542492
H	7.085279	1.896506	0.154574
H	0.353508	-1.034795	-4.611679
H	-0.124652	-1.278227	-2.918202
H	3.257877	1.789397	-5.932987
H	3.464397	0.927918	-3.603346
H	-1.801114	-4.330316	1.425945
H	1.894996	-0.464739	4.927236
H	7.172388	3.335509	-0.875766
H	-2.753243	-6.242839	0.108098
H	-0.544003	-1.943316	4.140485
H	-1.338591	-0.805709	-4.123321
H	1.503315	1.967656	-5.749454
H	-0.822682	-4.820684	-0.804165
H	1.560251	-0.454242	2.514717
H	1.631712	0.274624	-2.284928
H	-3.998612	-5.476533	1.113283
H	5.994477	3.264546	0.452912
H	-0.797603	-1.483497	2.470605
H	-0.770507	-3.088877	-0.469742
H	2.600014	2.982337	-4.791000
H	5.724683	3.709409	-2.707480
H	3.609810	1.352803	2.565711
H	4.132514	2.945851	-2.864750
H	-0.254356	1.430085	-4.379580
H	-3.928237	-3.004104	1.577589
H	-2.633310	-5.778830	-2.352908
H	-2.582041	-2.025995	0.998935
H	-0.102770	0.494674	4.739946
H	-0.721267	1.149528	-2.713602
H	2.355997	1.603815	3.805769
H	4.453317	4.048204	-1.519240
H	-4.857216	-5.849784	-1.198134

H	3.657823	2.791765	3.605103
H	3.941376	3.144090	0.919059
H	-0.349194	0.929490	3.036590
H	-1.600052	-3.534318	-2.796610
H	-1.696919	0.273621	3.989742
H	1.497423	2.145127	1.494590
H	-5.667296	-3.869271	0.045755
H	-3.792078	-4.678543	-3.124054
H	0.156184	3.437576	-3.560894
H	-2.469343	-1.525278	-1.647857
H	5.269950	5.361264	0.709530
H	1.427371	3.537057	2.534613
H	-5.591796	-3.537431	-1.693073
H	3.697731	4.921681	2.619508
H	-3.736361	-2.220025	-2.652393
H	3.942587	6.055349	-0.238479
H	-6.758581	-2.116472	-0.526476
H	-2.554331	1.442896	1.663788
H	-8.814544	-2.116493	0.328492
H	4.483932	6.857479	1.251001
H	2.366176	5.628128	1.728951
H	1.507325	5.674595	-0.167685
H	0.241525	5.560870	-2.291347
H	-0.817681	3.053990	0.620139
H	-3.866769	2.093438	2.638501
H	-8.680027	-0.246519	2.059979
H	-1.741944	3.400532	2.931348
H	-8.778631	-1.726183	-1.409692
H	-2.553984	2.057649	-0.969557
H	-10.195940	-1.294005	-0.428324
H	-0.879218	4.768648	1.030117
H	-10.110005	0.493838	1.288415
H	-8.633675	1.453010	1.545455
H	-6.795775	2.057424	0.385823
H	-3.880789	3.056714	-1.558380
H	-3.949982	4.525084	3.213341
H	-1.767915	4.377367	-1.257909
H	-5.683183	3.439453	1.657965
H	-2.766515	5.661790	2.538392
H	-8.603812	0.705831	-2.168758
H	-10.065443	1.054108	-1.204052
H	-5.686656	3.846896	-0.066732
H	-8.589472	2.020520	-0.974386
H	-2.784183	6.230205	0.099520
H	-3.981494	5.504634	-0.990662
H	-4.940258	5.773561	1.296431
Mg	1.727793	0.048368	0.109641
Mg	-0.984536	-0.001549	0.036270
N	3.144288	-1.205679	0.875809
N	3.075533	1.385313	-0.663839
O	-2.807449	-0.000022	-0.032922

6. References

[S1] S. Meiries, G. Le Duc, A. Chartoire, A. Collado, K. Speck, K. S. A. Arachchige, A. M. Z. Slawin, S. P. Nolan, *Chem. Eur. J.* **2013**, *19*, 17358–17368.

[S2] H. Lehmkuhl, K. Mehler, R. Benn, A. Rufiiiska, C. Krüger, *Chem. Ber.* **1986**, *119*, 1054–1069.

[S3] E. Hay, P. Hitchcock, M. Lappert, R. K. Audesh, *J. Organomet. Chem.* **1987**, *1-2*, 1-12.

[S4] T. X. Gentner, B. Rösch, G. Ballmann, J. Langer, H. Elsen, S. Harder, *Angew. Chem. Int. Ed.* **2019**, *58*, 607–611.

[S5] B. Rösch, T. X. Gentner, J. Eyselein, J. Langer, H. Elsen, S. Harder, *Nature* **2021**, *592*, 717–721.

[S6] P. S. Tanner, D. J. Burkey, T. P. Hanusa, *Polyhedron* **1995**, *14*, 331–333.

[S7] A. W. Duff, P. B. Hitchcock, M. F. Lappert, R. G. Taylor, *J. Organomet. Chem.* **1985**, *293*, 271–283.

[S8] T. Watanabe, Y. Ishida, T. Matsuo, H. Kawaguchi, *Dalton Trans.* **2010**, *39*, 484–491.

[S9] V. S. Parmar, G. K. Gransbury, G. F. S. Whitehead, D. P. Mills, R. E. P. Winpenny, *Chem. Commun.* **2021**, *57*, 9208–9211.

[S10] T. M. Rayder, E. H. Adillon, J. A. Byers, C.-K. Tsung, *Chem* **2020**, *6*, 1742–1754.

[S11] a) Rigaku Oxford Diffraction, **2020**, CrysAlisPro Software system, version 1.171.40.84a, Rigaku Corporation, Wroclaw, Poland (compound (BDI*)MgMgCp*); b) Rigaku Oxford Diffraction, **2021**, CrysAlisPro Software system, version 1.171.41.113a, Rigaku Corporation, Wroclaw, Poland (compound [(BDI*-H)Mg(H)CaCp*]); c) Rigaku Oxford Diffraction, **2022**, CrysAlisPro Software system, version 1.171.42.72a, Rigaku Corporation, Wroclaw, Poland (compound [(BDI)MgCp]); d) Rigaku Oxford Diffraction, **2024**, CrysAlisPro Software system, version 1.171.43.106a, Rigaku Corporation, Wroclaw, Poland (all other compounds).

[S12] R. C. Clark, J. S. Reid, *Acta Crystallogr., Sect. A: Found. Crystallogr.* **1995**, *51*, 887-897.

[S13] O. V. Dolomanov, L. J. Bourhis, R.J. Gildea, J. A. K. Howard, H. Puschmann, *J. Appl. Cryst.* **2009**, *42*, 339–341.

[S14] G. M. Sheldrick, *Acta Crystallogr., Sect. A: Found. Adv.* **2015**, *71*, 3–8.

[S15] G. M. Sheldrick, *Acta Crystallogr., Sect. C: Struct. Chem.* **2015**, *71*, 3–8.

[S16] P. van der Sluis, A. L. Spek, *Acta Crystallogr., Sect. A: Found. Crystallogr.* **1990**, *46*, 194–201.

[S17] A. Thorn, B. Dittrich and G. M. Sheldrick, *Acta Crystallogr., Sect. A: Found. Crystallogr.* **2012**, *68*, 448–451.

[S18] M. J. Frisch, G. W. Trucks, H. B. Schlegel, G. E. Scuseria, M. A. Robb, J. R. Cheeseman, G. Scalmani, V. Barone, G. A. Petersson, H. Nakatsuji, X. Li, M. Caricato, A. V. Marenich, J. Bloino, B. G. Janesko, R. Gomperts, B. Mennucci, H. P. Hratchian, J. V. Ortiz, A. F. Izmaylov, J. L. Sonnenberg, D. Williams-Young, F. Ding, F. Lipparini, F. Egidi, J. Goings, B. Peng, A. Petrone, T. Henderson, D. Ranasinghe, V. G. Zakrzewski, J. Gao, N. Rega, G. Zheng, W. Liang, M. Hada, M. Ehara, K. Toyota, R. Fukuda, J. Hasegawa, M. Ishida, T. Nakajima, Y. Honda, O. Kitao, H. Nakai, T. Vreven, K. Throssell, J. A. Montgomery, J. E. Peralta, F. Ogliaro, M. J. Bearpark, J. J. Heyd, E. N. Brothers, K. N. Kudin, V. N. Staroverov, T. A. Keith, R. Kobayashi, J. Normand, K. Raghavachari, A. P. Rendell, J. C. Burant, S. S. Iyengar, J. Tomasi, M. Cossi, J. M. Millam, M. Klene, C. Adamo, R. Cammi, J. W. Ochterski, R. L. Martin, K. Morokuma, O. Farkas, J. B. Foresman, D. J. Fox, *Gaussian 16 Rev. A.03*, Wallingford CT, **2016**.

[S19] A. D. Becke, *J. Chem. Phys.* **1993**, *98*, 5648–5652.

[S20] J. P. Perdew, J. A. Chevary, S. H. Vosko, K. A. Jackson, M. R. Pederson, D. J. Singh, C. Fiolhais, *Phys. Rev. B* **1993**, *48*, 4978–4978.

[S21] F. Weigend, *Phys. Chem. Chem. Phys.* **2006**, *8*, 1057–1065.

[S22] F. Weigend, R. Ahlrichs, *Phys. Chem. Chem. Phys.* **2005**, *7*, 3297.

[S23] S. Grimme, S. Ehrlich, L. Goerigk, *J. Comput. Chem.* **2011**, *32*, 1456–1465.

[S24] E. D. Glendening, J. K. Badenhoop, A. E. Reed, J. E. Carpenter, J. A. Bohmann, C. M. Morales, P. Karafiloglou, C. R. Landis, F. Weinhold, *NBO 7.0*, Theoretical Chemistry Institute, University of Wisconsin, Madison, **2018**.

[S25] N. J. R. van Eikema Hommes, *Molecule*, Erlangen, **2018**.

[S26] R. F. W. Bader, *Chem. Rev*. **1991**, *91*, 893–928.

[S27] T. A. Keith, AIMAll (Version 17.01.25), TK Gristmill Software, Overland Park KS USA, **2017**.
